# Maternal Transfer and Long-Term Population Effects of PCBs in Baltic Grey Seals Using a New Toxicokinetic–Toxicodynamic Population Model

**DOI:** 10.1007/s00244-022-00962-3

**Published:** 2022-10-15

**Authors:** Karl Mauritsson, Jean-Pierre Desforges, Karin C. Harding

**Affiliations:** 1 Division of Biology and Bioinformatics, University of Skövde, Skövde, Sweden; 2 Department of Environmental Studies and Sciences, University of Winnipeg, Winnipeg, MB Canada; 3 Department of Biological and Environmental Sciences, University of Gothenburg, Gothenburg, Sweden

## Abstract

Empirical evidence has shown that historical exposure of polychlorinated biphenyls (PCBs) to Baltic grey seals not only severely affected individual fitness, but also population growth rates and most likely caused the retarded recovery rate of the depleted population for decades. We constructed a new model which we term a toxicokinetic–toxicodynamic (TKTD) population model to quantify these effects. The toxicokinetic sub-model describes in detail the bioaccumulation, elimination and vertical transfer from mother to offspring of PCBs and is linked to a toxicodynamic model for estimation of PCB-related damage, hazard and stress impacts on fertility and survival rates. Both sub-models were linked to a Leslie matrix population model to calculate changes in population growth rate and age structure, given different rates of PCB exposure. Toxicodynamic model parameters related to reproductive organ lesions were calibrated using published historical data on observed pregnancy rates in Baltic grey seal females. Compared to empirical data, the TKTD population model described well the age-specific bioaccumulation pattern of PCBs in Baltic grey seals, and thus, the toxicokinetic parameters, deduced from the literature, are believed to be reliable. The model also captured well the general effects of PCBs on historical population growth rates. The model showed that reduced fertility due to increased PCB exposure causes decreased vertical transfer from mother to offspring and in turn increased biomagnification in non-breeding females. The developed TKTD model can be used to perform population viability analyses of Baltic grey seals with multiple stressors, also including by-catches and different hunting regimes. The model can also be extended to other marine mammals and other contaminants by adjustments of model parameters and thus provides a test bed in silico for new substances.

**Supplementary Information:**

The online version contains supplementary material available at 10.1007/s00244-022-00962-3.

During the past century, human activities have dramatically affected the ecological balance in the Baltic Sea. Recent assessments of biodiversity report inadequate status in all levels of the food web including indications of decreased nutritional status in fish and mammals (Kauhala et al. 2014; Hansson et al. 2017; HELCOM 2018b; Sonne et al. 2020). The semi-enclosed Baltic Sea is surrounded by nine highly industrialized countries and acts as a sink for municipal and industrial discharges and land run-off. Hazardous substances have been identified as one of the seven distinct threats to the Baltic Sea (HELCOM 2018b). The most harmful substances are those that are persistent, toxic and accumulate in organisms. These include among others persistent organic pollutants (POPs), and one of the POP classes of major concern is polychlorinated biphenyls (PCBs). Based on measurements of sediment accumulation, the PCB deposition in the Baltic Sea gradually increased during the 1940–1960s, reached peak values in the 1970s, substantially decreased during the late 1970s and 1980s and then continued to decline (Eckhéll et al. 2000). Despite the decline, concentrations of PCB in fish are still too high to be acceptable as food for humans according to the Swedish Food Administration (Bjurlid et al. 2018). The issue is even worse for marine mammals because they are long-lived top predators with large depots of blubber that can store fat-soluble chemicals over decades and recycle them across generations via vertical transfer to offspring during gestation and lactation (Bignert et al. 1998; Klanjscek et al. 2007; Desforges et al. 2012).

Four species of marine mammals occur in the Baltic Sea, whereof the grey seal (*Halichoerus grypus*) is the most abundant. The Baltic grey seal population approached 100,000 individuals in the early 1900s, but bounty hunting caused a decline to about 20,000 animals in the 1940s. When hunting ceased in 1974, the population did not increase as expected, and in the 1970s, the size of the Baltic grey seal population was estimated to only 3000 animals (Harding and Härkönen 1999). A likely explanation is a number of pathological changes, including reproductive failure, termed the Baltic Seal Disease Complex (BSDC) related to PCB exposure (Bergman and Olsson 1986; Bäcklin et al. 2003; Bredhult et al. 2008). Besides PCBs, POPs found in Baltic seals also include dioxins and furans, polychlorinated diphenyl ethers, toxaphenes and chlordanes, among many others (Nyman et al. 2002). PCB and DDT are considered as the most important factors behind BSDC, but they are probably not the only significant ones (Bergman 2007). Despite lower PCB levels in grey seals and their prey today than in the past, population health assessments have found that indicators of reproductive and nutritional status remain below identified threshold values, likely due to combined effects from multiple stressors like pollutants, climate change and reduced food availability (Bäcklin et al. 2011; Kauhala et al. 2012, 2014; HELCOM 2016; Silva et al. 2020; Sonne et al. 2020). Along with decreasing PCB levels during the last decades, the Baltic grey seal population has increased. The population size in 2020 was estimated to about 50 000 animals, but still the population growth rate is suppressed compared to healthy populations (HaV and SMHI 2022, Sköld 2022). The growing population has intensified conflicts between seals and commercial fisheries by resource competition and damages to catch and fishing gear (Tverin et al. 2019), and in 2001 hunting quotas have been increased up to 3000 seals (Bäcklin et al. 2011), without any risk assessment or population viability analysis. However, the hunt has not reached the annual quotas yet (Sköld 2022). To better understand long-term population effects from POPs and energetic stress, better models are urgently required.

Although the most conspicuous adverse effects of PCBs on Baltic grey seals, such as uterine occlusions, have declined during recent decades, PCBs may still affect the population through indirect effects such as impaired immunity which in turn influence risk of infection by pathogens and parasites (Bäcklin et al. 2011). The current main pressures to Baltic grey seals are hunting, by-catches in fishery, contaminants and climate change (HELCOM 2018b). More than 2000 grey seals are caught incidentally each year in the Baltic fisheries (Vanhatalo et al. 2014). Long-term climate change is predicted to cause shorter and warmer winters with decreasing sea ice coverage (Sundqvist et al. 2012). This will probably reduce the breeding success of Baltic grey seals, since pups born on land have a significantly higher mortality (Jüssi et al. 2008). It is of uttermost importance to develop risk assessment models that consider the sum of many smaller stressors and adverse effects on fitness in order to avoid another wave of overexploitation (Cervin et al. 2020; Silva et al. 2021).

The growth rate of a population is governed by its age-specific fecundity and survival rates. The long-term maximum growth rate of Baltic grey seals is limited by several factors. Females have at most one pup each year, of which 48% are female (HELCOM 2016). The first parturition occurs on average at an age of 5.5 years and not all females bear a pup each year, especially not young females. Some ovulating females do not complete pregnancy. Further, mortality of adults limits the population growth and senescence and pathological changes in reproductive organs decrease pregnancy rates. Environmental stress, such as parasites, pollution and limitations in food or breeding sites, reduce average fecundity and survival rates. Healthy, undisturbed populations of grey seals have an expected growth rate of 10% each year (Harding et al. 2007), but observed growth rates are significantly lower (HaV and SMHI 2022; Sköld 2022).

In order to better understand the temporal dynamics of persistent and toxic chemicals like PCBs in marine long-lived predators like the grey seal, it is necessary to develop appropriate modelling approaches. Models need to capture fine and long scale dynamics that apply over an animal’s life span. Since PCBs accumulate in a seal’s body during its whole life span, some health effects may arise only after many years. For instance, PCBs and other lipophilic POPs may have limited adverse effects on the health of an individual as long as they are bound in the blubber of the animal, but when blubber energy is mobilized to fuel various physiological processes during periods of food stress, pollutants are released to the blood and can circulate to sensitive tissues and organs and increase the risk of effects on fertility and survival (Lydersen et al. 2002; Klanjscek et al. 2007). Further, grey seals have some capacity to metabolise PCBs, using xenobiotic metabolising enzymes such as CYP1A (Nyman 2000). Studies of contaminant effects in marine mammals have focused largely on the molecular and individual levels, though some analyses on the population level exist (Pavlova et al. 2016; Hall et al. 2018; Silva et al. 2020). A reason for the limited number of population analyses is probably lack of long-term monitoring studies and data on contaminant levels and associated health effects. To characterize and predict contaminant levels and effects in populations of marine mammals over time, and thus to assess the effectiveness of past, current, or planned management and mitigation efforts, it is necessary to develop a mechanistic understanding of the processes of bioaccumulation (at different temporal scales) and intergenerational transfer as well as to understand how contaminants impose hazard or stress to individuals and how this relates to population dynamics.

Here a population model is developed and applied to predict the temporal dynamics of PCBs and its associated influence on vital rates, population growth, and extinction risk of Baltic grey seals over the past century. The model links dietary PCB exposure to internal concentrations, characterizes vertical transfer from mother to offspring, and relates dynamic internal concentrations to adverse effects on fertility and survival, then integrates this toxicokinetic and toxicodynamic (TKTD) model into an age-structured Leslie matrix model of temporal population dynamics. We focus on PCBs as a model contaminant because of the availability of empirical data, including time trends for PCB levels in Baltic fish and seals as well as frequencies of PCB-related reproductive defects in Baltic grey seal females. Despite the focus on Baltic grey seals, the model framework is broadly applicable to other marine mammals and bioaccumulative contaminants through informed changes to model parameters. Thereby, the model can be a useful tool in future viability assessments, targeting bioaccumulating contaminants in populations of marine mammals.

## Methods

### Model Overview

**Fig. 1:**
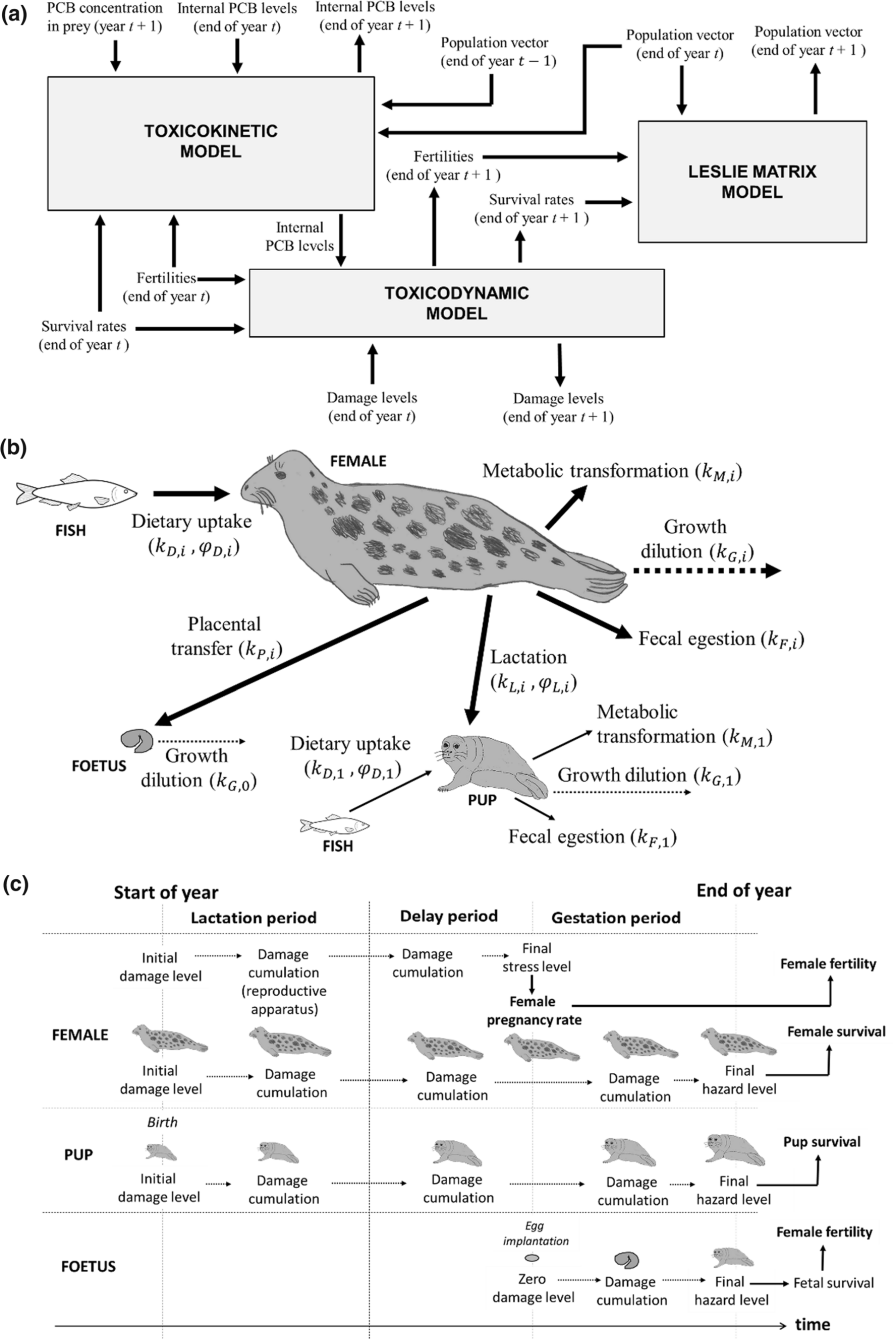
**a** Overview of the TKTD population model. Text boxes represent sub-models, performing calculations for year $t+1$. Free texts are inputs and outputs, with arrows showing relations to sub-models. **b** Bioaccumulation model showing the major routes for uptake, transfer and elimination of PCBs in grey seals, including associated rate constants (${k}_{\mathrm{D},i},{k}_{\mathrm{M},i},{k}_{\mathrm{F},i},{k}_{\mathrm{L},i},{k}_{\mathrm{P},i},{k}_{\mathrm{G},i}$) and assimilation efficiencies (${\varphi }_{\mathrm{D},i},{\varphi }_{\mathrm{L},i}$). **c** Effects of PCBs on survival and fertility considered in the toxicodynamic model as consequences of damage cumulation in females, pups and foetuses during a year

**Fig. 2:**
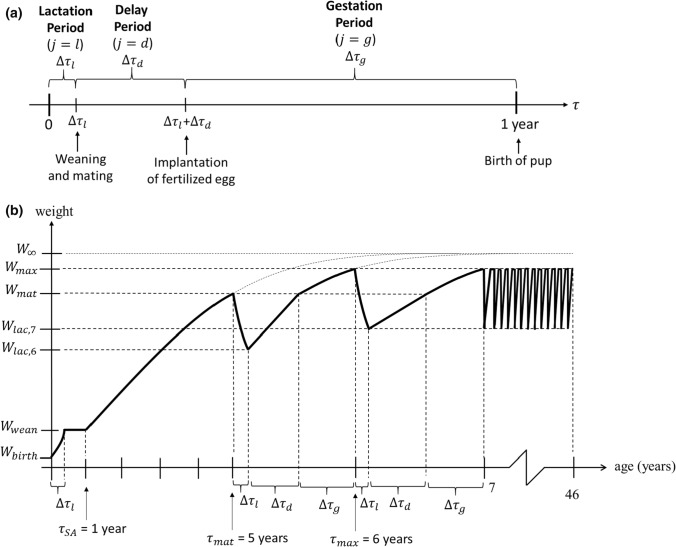
**a** Division of a year into three periods, representing the reproduction cycle of grey seal females. $\Delta {\tau }_{l}=18 \mathrm{days}, \Delta {\tau }_{d}=100 \mathrm{days}$, $\Delta {\tau }_{g}=247 \mathrm{days}$. **b** Body weight as a function of age according to growth model. Pups increase their weight exponentially during lactation and keep it constant during the remainder of the year. Sub-adults follow von Bertalanffy growth function until maturation. Mature females lose weight exponentially during lactation, regain it linearly during the delay period and grow according to von Bertalanffy during gestation. The same yearly pattern for mature females is then repeated

Our matrix model, called the TKTD population model*,* for analysing adverse effects of PCBs on Baltic grey seals was developed to link toxicokinetics (TK) of dietary and vertically transferred PCB accumulation to toxicodynamics (TD) of adverse effects on reproduction and survival to ultimately predict changes to population demographics and growth rates (Fig. 1a). The TKTD model is used to modify survival and fecundity parameters of an age-structured Leslie matrix model for an ideal Baltic grey seal population due to internal PCB concentrations following DEBtox and threshold damage model (TDM) principles of damage, hazard, stress and recovery. While the model runs on an annual basis for population dynamics, each year is divided into three periods (*lactation, delay* and *gestation*), representing different life history phases for a reproducing grey seal female (Fig. 2a). The year starts with the *lactation period* (lasting 18 days), during which pups are nursed by their mothers. After weaning, females mate and the *delay period* starts (lasting 100 days), after which the fertilized egg is implanted. During the *gestation period* (lasting 247 days), the foetus develops in the uterus (SI-Table 2). The year ends with the birth of new pups. Since all deaths are assumed to occur just before the birth events, all pups that are born have mothers that nurse them. Different population-level TD effects of PCBs occur after specific time periods (e.g. some fertility effects are estimated after the delay period).

The model is used to perform stepwise annual calculations of PCB concentrations, which are converted to adverse effects through calculation of damage, hazard and stress levels (see below for details), which ultimately determine fertilities, survival rates and number of individuals in different age classes. The TKTD population model consists of the following three sub-models (Fig. 1a): (1) Toxicokinetic model: bioaccumulation model for dietary uptake, elimination and vertical transfer through placenta (during gestation) and breast milk (during lactation); (2) Toxicodynamic model: TDM for damage and recovery as response to internal toxicant levels, obtained from the toxicokinetic model; cumulative damage causes hazard/stress to foetuses, pups and females, resulting in reduced fertilities and survival rates; and (3) Leslie matrix model: age-structured matrix model for population growth, with baseline ideal fertility and survival rates adjusted according to the toxicodynamic model of PCB effects.

A brief description of the model framework is provided here, and a complete detailed description and equation formulation is provided in the Supplemental Information (SI). Environmental variables and initial conditions are first established. These include initial seal PCB concentrations, initial damage levels, initial population vector and annual prey PCB concentrations (SI-Table 1, SI-Fig. 8). The initial population is calculated from the stable age distribution for a population with ideal fertilities and survival rates (SI-Table 4), based on an initial female population size of 8820 individuals in the year 1961 (Harding and Härkönen 1999). New state variables are calculated for one year at a time, based on values of state variables the previous years. For each year, the following calculations are performed, and the procedure is repeated until the final year: (1) calculation of PCB concentration in foetuses, pups and females as a result of bioaccumulation and vertical transfer from females to offspring; (2) calculation of cumulated damage in foetuses, pups and females, based on internal PCB concentrations; (3) calculation of hazard/stress in foetuses, pups and females, based on cumulated damages; (4) calculation of reduced fertilities, based on foetal hazard and female stress (accounting for damage to the reproductive apparatus); (5) calculation of reduced survival rates, based on hazard in pups and females; and (6) calculation of new population size and structure, based on reduced fertilities, reduced survival rates, and previous year’s population data. It is assumed that all deaths and births occur at the end of the year, though damage cumulates during the year.

The TKTD population model was implemented as a set of functions and script files in the numerical software MATLAB® (Mathworks Inc., Natick, MA, USA), and all analyses were performed with these.

### Toxicokinetic Model

The toxicokinetic model describes PCB bioaccumulation in offspring and females through dietary uptake and elimination (metabolic transformation and faecal egestion) as well as vertical transfer from mother to embryo through the placenta during gestation and from mother to pup through breast milk during lactation (Fig. 1b). During lactation, neither the mother nor the pup consumes prey, thus the primary exchange of PCBs occurs through vertical transfer via the milk (including environmental losses). During the *delay period*, both females and pups feed on prey. During the *gestation period*, females and pups continue to feed, but females also transfer PCB to foetuses through the placenta.

#### Growth Model

Dilution change of PCB concentration due to increased body size as individuals grow (*growth dilution*) is captured in the TK model through a generalized sub model describing animal growth (Fig. 2b). Body size is also an important variable since it affects rates of feeding, vertical transfer and PCB elimination. Foetuses and pups are assumed to grow exponentially during the gestation and lactation periods. After weaning, grey seal pups typically lose weight during some months due to reduced nutritional intake, but when they become more skilled hunters they start to regain weight (Kauhala et al. 2017). For simplicity, it is here assumed that pups keep the weaning weight until they reach sub adult age (1 year). Grey seal females typically reach asymptotic body size at age six, about the time when they become sexually mature. When they nurse pups, they lose a considerable amount of weight, but this is regained when they feed during the remaining part of the year. Our model assumes a von Bertalanffy growth function to describe body growth of grey seal females from weaning to sexual maturity. Once females reach mature body length, they start to breed. We assume that growth dilution rates are approximately constant within each period of a year (delay, gestation, lactation).

Since primiparous females (first-time breeders) are typically smaller than multiparous females (experienced breeders) (Lang and Iverson 2009), the mature body weight ${W}_{\mathrm{mat}}$ is lower than the maximum body weight ${W}_{\mathrm{max}}$, reached by multiparous females just before they start the lactation period. Since females lose significant weight during lactation, it is assumed that their weight decreases exponentially throughout the period, from the initial body weight (${W}_{\mathrm{mat}}$ or ${W}_{\mathrm{max}}$) to the lactation body weight (${W}_{\mathrm{lac},6}$ or ${W}_{\mathrm{lac},7}$). During the delay period, mature females are assumed to linearly regain the weight they lost during lactation. During the succeeding gestation period, they continue to grow according to von Bertalanffy and reach the maximum body weight ${W}_{\mathrm{max}}$ at the end of the year. The succeeding years, females are assumed to repeat the pattern of exponential decline (lactation period), linear regain (delay period) and von Bertalanffy growth (gestation period). Values of parameters used to describe body growth were chosen based on published data for Baltic grey seals (SI-Table 2).

#### Bioaccumulation Model

Bioaccumulation in females (age class 2 to 46) includes dietary uptake, removal (metabolic transformation and faecal elimination) and vertical transfer to offspring (Fig. 1b). The rate of change of total amount of PCBs in a female is expressed as the rate by which PCBs are assimilated (assumed to be proportional to the concentration in the prey) subtracted by the rate by which PCBs are eliminated (assumed to be proportional to the concentration in the body). Thus, the concentration of PCBs in females of age class *i* during time period *j* (${c}_{i,i}^{j}$) can be approximated using the following first-order ordinary differential equation:

$$ \frac{{{\text{d}}c_{i,i}^{j} }}{{{\text{d}}\tau }} = k_{{{\text{D}},i}}^{j} \overline{c}_{{{\text{D}},i}} - \tilde{k}_{{{\text{E}},i}}^{j} c_{i,i}^{j} ,\quad i = 2, \ldots , 46,\quad j = l,d, g $$

where ${k}_{\mathrm{D},i}^{j}$ is the PCB dietary uptake rate in females of different age classes and time periods, ${\overline{\mathrm{c}} }_{\mathrm{D},i}$ is the mean dietary PCB concentration and ${\widetilde{k}}_{\mathrm{E},i}^{j}$ is the effective PCB elimination rate (accounting for removal, vertical transfer and growth dilution). The time period *j* enters three different states during a year; lactation (*l*), delay (*d*) and gestation (*g*). The repeated notation of index *i* in female concentrations (${c}_{i,i}^{j}$) is applied for technical reasons, enabling compact general descriptions of all age classes and foetuses. Dietary uptake rates are a function of the prey consumption rate (kg/year) and the diet assimilation efficiency. We follow the general principles of DEB theory such that food uptake is proportional to the body surface area and a fixed prey consumption factor based on empirically derived maximum yearly food intake. We assume that mean PCB concentration in prey is constant throughout the year. The diet includes fish of different species and each seal age class consumes a diet of specific prey composition. According to Hansson et al. (2017), 80% of the Baltic fish biomass is constituted by sprat (*Sprattus sprattus*), herring (*Clupea harengus*) and cod (*Gadus morhua*). It was assumed that Baltic grey seals feed exclusively on these three species. Prey preference indices, describing the preference of prey items by age class, were calculated from published mean fractions of total prey biomass found in analyses of gut content in Baltic grey seals (Lundström et al. 2010; Tverin et al. 2019) (SI-Table 3). Dietary PCBs are thus a function of the mean weight fraction of each prey item and their respective PCB levels. Elimination of PCBs is accounted for through fixed rate constants for removal, placental transfer, lactational transfer and growth dilution. All rate constants are age-specific, though some of them have similar or same values between age classes. We use estimated removal rate constants from Hickie et al. (2005) for total PCBs in ringed seals after scaling for differences in body size between species and age classes, assuming that removal rate constants scale like mass-specific metabolic rates according to Kleiber’s law (Kleiber 1962) (SI-Table 6).

Foetuses assimilate PCBs only through vertical transfer via the placenta (Fig. 1b). It is assumed that all PCBs that are eliminated by a mother through placental transfer are assimilated by her foetus, which has no capacity to eliminate them. The concentration of PCB in the foetus (${c}_{0,i}^{g}$) is therefore a function of the PCB concentration in the mother of age class *i* during the gestation period (*j* = *g*) (${c}_{i,i}^{g}$), the body weight ratio between mother and fetus (${\omega }_{0,i}$), the placental transfer rate constant (${k}_{P,i}$), and fetal growth dilution (${k}_{G,0}$), governed by the following differential equation:

$$ \frac{{{\text{d}}c_{0,i}^{g} }}{{{\text{d}}\tau }} = k_{{{\text{P}},i}} \omega_{0,i} c_{i,i}^{g} - k_{{{\text{G}},0}} c_{0,i}^{g} ,\quad i = 2, \ldots , 46 $$

The accumulation of PCBs in pups (*i* = 1) is calculated similar to that of older age classes, but with no placental transfer and a positive contribution from vertical transfer through lactation (Fig. 1b). In other words, the PCB concentration in pups (${c}_{1,i}^{j}$) is a function of maternal PCB concentrations (${c}_{i,i}^{j}$), the body weight ratio between mother and pup (${\omega }_{1,i}$), the lactation PCB transfer rate (${k}_{\mathrm{L}1,i}^{j}$), the annual PCB dietary uptake rate of pups (${k}_{\mathrm{D},1}^{j}$), the mean dietary PCB concentration (${\overline{c} }_{\mathrm{D},1}$) and the effective PCB elimination rate of the pup (${\widetilde{k}}_{\mathrm{E},1}^{j}$), governed by the following differential equation:

$$ \frac{{{\text{d}}c_{1,i}^{j} }}{{{\text{d}}\tau }} = k_{{{\text{L}}1,i}}^{j} \omega_{1,i} c_{i,i}^{j} + k_{{{\text{D}},1}}^{j} \overline{c}_{{{\text{D}},1}} - \tilde{k}_{{{\text{E}},1}}^{j} c_{1,i}^{j} , \quad i = 2, \ldots , 46,\quad j = l,d, g $$

#### Conversion of PCB Concentrations

The TK model estimates total body concentrations of PCBs for seals and fish, while empirical data are reported on a lipid weight basis. We thus convert calculated whole-body PCBs to lipid concentrations assuming all PCBs in the body of a seal is bound in fat tissue. Thus, the concentration in lipids is calculated as the concentration in the body divided by the body fat index (total lipid weight/total body weight). As a simplification, it is assumed that the mean body fat index is 30% for all age classes at field sampling in autumn when seals are at their fattest (Iverson 2002; Kauhala et al. 2017). The prey lipid PCB concentration is calculated in the same way as seals. All numerical values of PCB concentration refer to sum-PCB (total concentration of all PCB congeners).

### Toxicodynamic Model

The toxicodynamic model describes how PCB body loads of foetuses, pups, and females cause cumulation of damage, hazard and stress, and how these are translated into reduced survival and fertility (Fig. 1c, SI-Figs. 6, 7). The toxicodynamics are based on the threshold damage model (TDM), introduced by Ashauer et al. (2007b). TDM is based on DEBtox theory but adds cumulative damage effects and ability to recover from damage. Internal contaminants induce *damage,* which accounts for physiological disturbances from all kinds of biochemical and physiological processes involved in toxicity. *Hazard* is probability to die during a time interval based on the cumulative amount of accrued damage. Recovery is included as a physiological ability to repair damages caused by internal contaminants, for instance cell repair mechanisms and physiological adaptations.

The current TD model follows the general principles of DEBtox in assuming that the toxic effects of PCBs on grey seals are considered through the lens of particular physiological modes of action (pMoAs) affecting fundamental physiological processes like somatic maintenance or maturation. While our model is not based on DEB theory in the description of energetics and life history traits, we interpret internal PCB concentration effects using the lens of DEBtox pMoAs with a hazard model that represents damage to reproductive organs and mortality of foetuses, pups and females. In this context, we assume that decreased survival results from increased costs of somatic maintenance (e.g. repair of lesions) due to cumulative damage causing hazard in pups, juveniles and females. Decreased reproduction results from increased foetal hazard (foetal mortality) and female reproductive stress. Assuming a hazard model (Billoir et al. 2007), we consider *reproductive stress* as an indirect effect on reproduction via increased costs for maintenance of the reproductive apparatus due to cumulated damage (e.g. reproductive organ lesions). Other indirect toxicodynamic effects on reproduction, such as decreased feeding rate and increased growth costs, are neglected. Notice that three different kinds of damage are considered: (1) damage to pups and females (causing hazard that decreases survival); (2) damage to reproductive apparatus (causing stress that reduces fertility); and (3) damage to foetuses (causing hazard that kills them and reduces the fertility of their mothers). Since foetuses and pups do not invest energy in reproduction, damage to reproductive organs begins to cumulate when seals reach the age of 2.

Our TD model follows the TDM approach to calculate damage and recovery (Ashauer et al. 2007b). The cumulation of all damage types is described by the same governing equations, which only differ in values of model parameters (SI-Table 7). According to TDM, the PCB-induced damage ${d}_{k,i}^{j}$ increases at a rate that is linear with the internal PCB concentration ${c}_{k,i}^{j}$, whereas the recovery rate is proportional to the current damage:

$$ \frac{{{\text{d}}d_{k,i}^{j} }}{{{\text{d}}\tau }} = \sigma_{k} c_{k,i}^{j} - r_{k} d_{k,i}^{j} ,\quad k = 0, 1, i, \quad i = 2, \ldots , 46,\quad j = l, d, g $$

here ${\sigma }_{k}$ is the killing/stress rate constant and ${r}_{k}$ is the recovery rate constant. Hazard ${h}_{k,i}^{j}$ (or stress) is obtained if the damage level exceeds an age-specific threshold ${d}_{T,k}$:

$$ h_{k,i}^{j} = \left[ {d_{k,i}^{j} - d_{{{\text{T}},k}} } \right]_{ + } ,\quad \left[ x \right]_{ + } = \max \left( {0,x} \right) $$

Fertilities are affected by PCB exposure in two different ways, reduction due to reproductive stress and reduction due to foetal hazard. It is here assumed that the state of females at the end of the delay period (just before gestation starts) determines fertility reduction due to reproductive stress (e.g. reproductive lesions) and that the state of foetuses at the end of the gestation period determines fertility reduction due to foetal hazard. Survival rates of pups and females ${P}_{i}(t)$ are decreased by hazard values ${\widehat{h}}_{i}^{g}(t)$ at the end of the year (*t*) through a reduction factor that is multiplied to the baseline survival value ${P}_{i}^{0}$:

$$ P_{i} \left( t \right) = P_{i}^{0} e^{{ - \hat{h}_{i}^{g} \left( t \right)}} , \quad i = 1, \ldots , 46 $$

Similarly, fertility impacts are also implemented as a reduction factor (hazard/stress) multiplied to the baseline age-specific fertility value. The three sets of TD parameters include *reproductive stress* parameters, *foetal hazard* parameters, and hazard parameters related to *survival* of pups and adults. Each parameter set includes three type of constants: *stress/killing rate constants*, *damage threshold levels* and *recovery rate constants* (SI-Table 7). With respect to the many empirical observations of reproductive organ lesions in Baltic grey seals during the 1970s and the 1980s, stress to the reproductive apparatus is probably the most crucial path in which PCBs affect vital rates. The reproductive stress parameters were estimated by a calibration procedure where pregnancy rates predicted by the TKTD population model were compared to reported pregnancy rates (Roos et al. 2012). For the other toxicodynamic parameters, values were chosen based on parameters estimated in Desforges et al. (2017) for their model on PCB exposure and effects in captive fed mink. Since the mink model did not account for cumulated damage and recovery, we had to estimate these TDM model parameters using exposure times for the mink dataset as well as several simplifying assumptions described in the SI. Ultimately, estimated mink TDM parameters were converted to grey seal TDM parameters assuming that contaminant sensitivity (no effect concentrations and damage threshold levels) among species scales with specific metabolic rates (Baas and Kooijman 2015) (SI-Table 7). We assumed that tolerance parameters are the same for different mammals exposed to a specified contaminant, and thus killing rate constants also remain unchanged when exposure times are similar.

### Leslie Matrix Model

The Leslie matrix model follows Harding et al. (2007) to implement a full age-structured model for Baltic grey seals using annual time steps and 46 age classes. Since an insufficient number of males is unlikely to restrict growth of a seal population (Harding et al. 2007), only the size of the female population is considered. Individuals of age class $i=1$ (with an age of 0–1 year) are referred to as *pups*, whereas individuals of age class $i=2, \dots , 46$ are referred to as *females*. Ideal vital rates (${F}_{i}^{0}$ and ${P}_{i}^{0}$) for a population with maximal possible reproduction, derived by Harding et al. (2007), were used as baseline values in our model, representing fertilities and survival rates in the absence of PCB exposure (SI-Table 4). These vital rates result in a predicted maximum possible population growth rate of $\lambda =1.10$.

Unlike Harding et al. (2007), the Leslie matrix elements in our model are not constants. Fertilities (${F}_{i}$) and survival rates (${P}_{i}$) are linked to hazard/stress levels resulting from cumulated damage levels as a result of the history of internal PCB concentrations, which in turn depend on the history of PCB concentrations in the prey. It is assumed that all deaths occur at the end of a year and immediately after, all pups are born. Since only females that survive to the end of a year give birth to new pups, the first row of the Leslie matrix contains products of survival rates and fertilities, also a difference from Harding et al. (2007). Since the population size is accounted for just after breeding, the Leslie matrix model may be characterized as a *post-breeding model*:

$$ \begin{aligned} & {\mathbf{n}}\left( {t + 1} \right) = {\mathbf{A}}\left( {t + 1} \right){\mathbf{n}}\left( t \right), \\ & {\mathbf{A}}\left( t \right) = \left[ {\begin{array}{*{20}c} {P_{1} \left( t \right)F_{1} \left( t \right)} & {\quad P_{2} \left( t \right)F_{2} \left( t \right)} & {\quad \cdots } & {\quad P_{45} \left( t \right)F_{45} \left( t \right)} & {\quad 0} \\ {P_{1} \left( t \right)} & {\quad 0} & {\quad \cdots } & {\quad 0} & {\quad 0} \\ 0 & {\quad P_{2} \left( t \right)} & {\quad \cdots } & {\quad 0} & {\quad 0} \\ \vdots & {\quad \vdots } & {\quad \ddots } & {\quad \vdots } & {\quad \vdots } \\ 0 & {\quad 0} & {\quad \cdots } & {\quad P_{45} \left( t \right)} & {\quad 0} \\ \end{array} } \right] \\ \end{aligned} $$

The population vector $\mathbf{n}(t)$ includes the number of individuals in different age classes at the end of year $t$. The Leslie matrix $\mathbf{A}(t)$ includes fertilities and survival rates at the end of year $t$.

#### Density Dependence

Recent observations of reduced pregnancy rates in Baltic grey seals have been explained as a consequence of the population approaching carrying capacity (Kauhala et al. 2014) and observations of thinner blubber layers have been explained by food limitation (HELCOM 2018a). Fertilities and survival rates are thus assumed to be affected by population density. We set the carrying capacity to 100 000, since the Baltic grey seal population likely approached this size in the early 1900s (Harding and Härkönen 1999). It was assumed that adult survival and fertility are only dependent on the number of adults, not on the number of pups. Since pups are more sensitive to harsh conditions than older animals, it was also assumed that pup survival is more affected by population density than other age classes. Density-dependent changes in age-specific survival and fertility were calculated from population size and age class specific density effect factors (SI-Table 5). The adult density effect factor was parameterized such that the total population (females and males) approached carrying capacity under ideal conditions (no adverse effects from contaminants).

### Model Simulations

#### Steady-State Simulations

If prey PCB concentrations are constant over time, a stable state will finally be reached where PCB, damage, hazard and stress levels are stationary. If density dependence is neglected, also fertilities and survival rates stabilize at constant values and the population grows (or declines) at constant rate. Under these conditions, biomagnification factors and stable population growth rate can be defined. Age-specific biomagnification factors (based on lipid concentrations) were calculated as the ultimate concentration ratios in seals and their prey. The stable population growth rate is calculated as the dominant eigenvalue of the Leslie matrix. For steady-state simulations we tested the effect of different levels of constant sum-PCB lipid concentrations in prey (0–10 mg/kg) and run the TKTD population model for 100 years. Endpoints of interest were simulated temporal changes in mean sum-PCB lipid concentrations and population size for Baltic grey seals.

#### Sensitivity Analysis

To describe the impact of parameter uncertainty on model outputs we run a global sensitivity analysis (change one parameter at a time). A sensitivity analysis investigates how uncertainty in input parameters causes uncertainty in population growth and can be used to identify parameters that are critical for population viability (Lacy et al. 2018). Given that Harding et al. (2007) performed a detailed sensitivity analysis of their grey seal Leslie matrix model for Leslie matrix parameters, we did not perform a similar analysis here. Instead, we focus the sensitivity analysis to TK and TD parameters, assuming steady-state conditions. This analysis was performed for three of the TK model parameters: the lactational transfer rate constants ${k}_{\mathrm{L},i}$, the placental transfer rate constants ${k}_{\mathrm{P},i}$ and the removal rate constants ${k}_{\mathrm{R},i}$ (accounting for the combination of metabolic transformation and fecal egestion). Ratios between lactational transfer rate constants of different age classes were held fixed under perturbations, whereas the placental transfer rate constants were the same for all fertile age classes. The removal rate constant ${k}_{\mathrm{R},i}$ were dependent on age class through body mass. Sensitivity was also assessed against three TD model parameters across life stages: killing/stress rate constants (${\sigma }_{0}$, ${\sigma }_{i}$, ${\widetilde{\sigma }}_{i}$), recovery rate constants (${r}_{0}$, ${r}_{i}$, ${\widetilde{r}}_{i}$) and damage threshold levels (${d}_{\mathrm{T},0}$, ${d}_{\mathrm{T},i}$, ${\widetilde{d}}_{\mathrm{T},i}$). A constant PCB lipid concentration in prey, generating positive stable growth rate over time, was used as input and the toxicokinetic rate constants were varied one at time, whereas other parameters were held constant. Stable population growth rate was plotted as a function of the perturbed relative value of investigated model parameters, defined as the perturbed value (${p}_{\mathrm{pert}}$) divided by the value adopted in the model (${p}_{\mathrm{mod}}$): ${p}_{\mathrm{rel}} ={p}_{\mathrm{pert}}/{p}_{\mathrm{mod}}$.

#### Realistic Simulations of Temporal Dietary PCBs

Segerstedt (2019) compiled data from previous studies of PCB concentrations in grey seals and their primary prey items (cod, sprat, and herring) from all regions of the Baltic Sea during 1966–2015, converted all concentrations to a lipid weight basis and clustered data into time intervals of 5 years. We linearly interpolated prey PCBs concentrations over each five-year period to obtain yearly PCB concentrations, used as input to the toxicokinetic model (Fig. 4a; SI-Fig. 8). The TKTD population model was used to run simulations for the time period 1966–2015, where data on PCB levels in prey and seals were available. A five-year pre-simulation period (1961–1965) was included to initiate realistic values of PCB and damage levels. Since PCB levels in Baltic fish were low in the early 1960s (Bignert et al. 1998), all PCB, damage, and hazard/stress levels were put to zero at the start of year 1961. Prey concentrations were assumed to increase linearly from zero (at 1961) to the reported levels 1966. The total population size year 1961 has been estimated to 17 639 seals, including males and females (Harding and Härkönen 1999). The initial population size in the simulation (accounting for females exclusively) was put to the half of that amount (8820 seals). Simulation outputs for temporal PCB levels in seals and population size were compared to empirical data.

## Results

### PCB and Population Dynamics with Constant Exposure

**Fig. 3:**
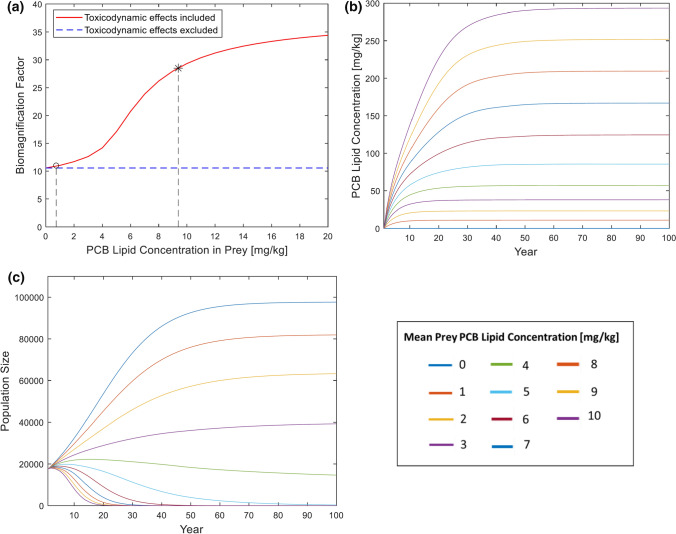
Steady-state model results. **a** Mean biomagnification factors (from prey to seals) predicted by the TKTD population model under steady-state conditions for different levels of constant mean sum-PCB lipid concentration in prey. Different results are obtained depending on whether toxicodynamic effects (adverse effects of PCB on fertilities and survival rates) are included. Indicated are the historical peak level of 9.4 mg/kg ($*$) and the 2015 level of 0.73 mg/kg (o). **b** Mean sum-PCB lipid concentration in seals for different scenarios of constant sum-PCB lipid concentrations in prey (ranging from 0 to 10 mg/kg) for a 100-year simulation period. PCB concentrations in seals are zero at the start of the first year. **c** Population size in seals for different scenarios of constant sum-PCB lipid concentrations in prey (ranging from 0 to 10 mg/kg) for a 100-year simulation period

Simulating time-constant prey PCB concentrations in our population TKTD model results in steady-state dynamics for PCB and population metrics over time (Fig. 3). The mean biomagnification of PCBs from fish to seals (all age classes included) increased with prey PCB concentrations up to a plateau of approximately 35 as prey levels reached 20 mg/kg lw (Fig. 3a). Assuming only TK processes (no TD effects), the biomagnification factor remains constant at 11 independent of prey PCB concentrations (Fig. 3a). Using the observed range of mean prey sum-PCB levels since the 1960s (0–10 mg/kg lw), steady-state mean sum-PCB levels for seals of all age classes over a 100-year simulation period were predicted (Fig. 3b). Here, PCB levels in seals increase over time for each constant diet exposure scenario and reach steady-state levels after 10 to 40 years. In accordance with the biomagnification results, higher prey PCBs resulted in higher seal PCB levels and longer times to reach steady state. While population sizes approach different stable levels for each constant diet exposure, the population growth rate decreases over time and approaches zero due to PCB impacts on fertility and survival (Fig. 3c). In the absence of PCBs, the population approaches its carrying capacity of 100,000 animals and increasing exposure to dietary PCBs reduces relative population size and growth rates, with a constant dietary sum-PCB level of approximately 4 mg/kg lw representing a critical threshold in which the growth rate is negative and the population size starts to decline over time (Fig. 3c).

### PCB and Population Dynamics with Time Varying Exposure

**Fig. 4:**
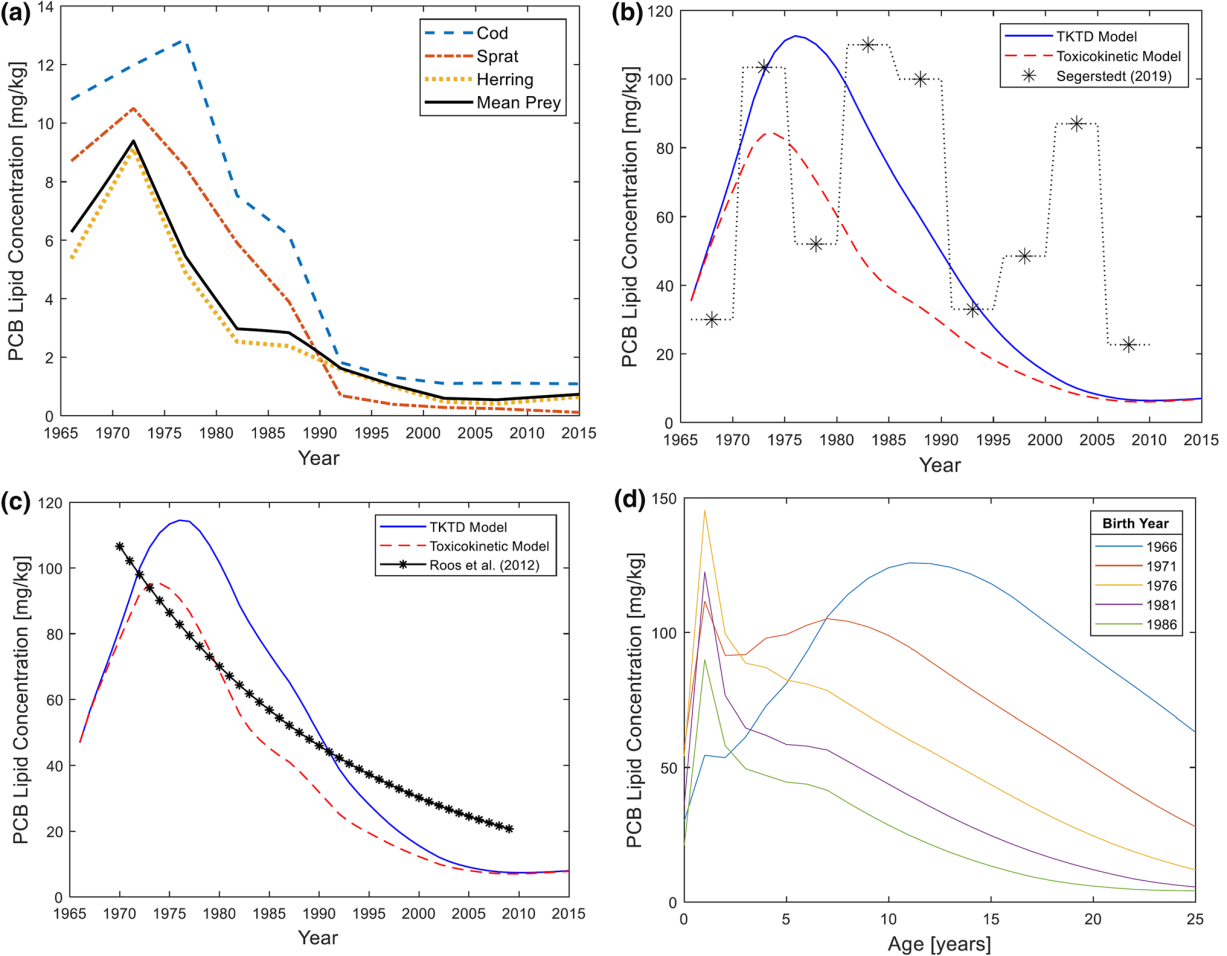
Time-varying dietary exposure model results for PCB dynamics. **a** Temporal sample mean sum-PCB lipid concentrations in different prey species from the Baltic Sea during 1966–2015, according to data compiled by Segerstedt (2019). **b** Mean sum-PCB lipid concentrations in female Baltic grey seals (*i* = 1–46) between 1966 and 2015. Shown are model predictions and published data (Segerstedt 2019). **c** Mean sum-PCB lipid concentrations in juvenile Baltic grey seals (*i* = 1, 2, 3) according to model predictions and published data (Roos et al. 2012). **d** Mean age-specific sum-PCB lipid concentrations in different cohorts of female Baltic grey seals between 1966 and 2015

We used historical fish PCB data on three important prey items for Baltic grey seals (Fig. 4a) to model temporal PCB dynamics in grey seal females across their full life cycle (Fig. 4b–d). The predicted lipid concentrations of PCBs in female seals are higher when TD processes are included (full model) as compared to a model with only TK process (Fig. 4b–c). Model predicted PCB levels followed observed temporal patterns for all seals (Fig. 4b) and especially for juveniles (aged 0–3 years) (Fig. 4c). Modelled PCBs in seals of all age classes followed observed declines in prey levels over time, though empirical data for seals fluctuated to much greater degree across years at short and long timespans (Fig. 4b). The peak PCB level in seals neared 120 mg/kg lw around 1976, which was several years later than peak PCB levels in sprat and herring but similar to cod (Fig. 4a–c). Following lifetime PCB profiles of seals born at different years revealed contrasting patterns over time (Fig. 4d). Consistent across almost all cohorts is the large vertical transfer of PCBs from mother to pup during lactation that results in peak lifetime PCB levels in pups. This is followed by a period of growth dilution when PCB levels decline as the animal grows rapidly during juvenile years and finally by decreasing concentrations in adults over time because of recurring vertical transfers (Fig. 4d). The 1966 cohort is unique as it was exposed to the greatest historical levels of PCBs and consequently had the greatest PCB impacts on reproduction, which was captured by the increasing PCB levels in young adult females that could not reproduce and offload their PCB burdens (Fig. 4d).

**Fig. 5:**
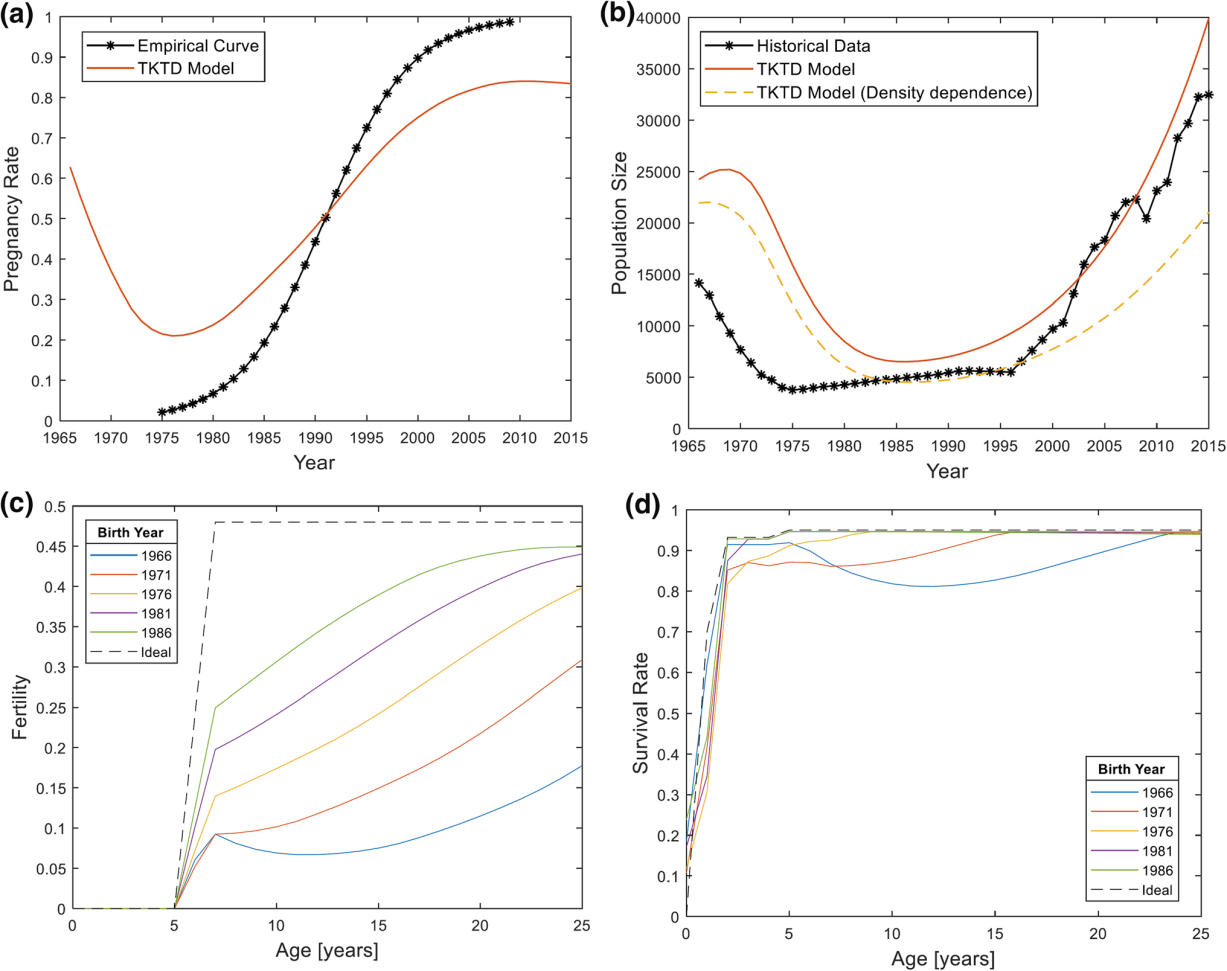
Time varying dietary exposure model results for population dynamics between 1966 and 2015. **a** Pregnancy rates from Roos et al. (2012) and mean pregnancy rates for females of ages 5–46 years according to the TKTD population model. **b** Population size according to historical data (HaV and SMHI 2022) and the TKTD population model. **c** Age-specific fertility rate in different cohorts of female Baltic grey seals between 1966 and 2015. **d** Age-specific survival rate in different cohorts of female Baltic grey seals between 1966 and 2015

Empirical data on pregnancy rates in Baltic grey seals showed near zero values in the 1970s and returned to near 100% in 2015 (Fig. 5a). Model predictions for pregnancy rates followed the temporal increase in pregnancy rates observed in the empirical data, though the model overpredicted pregnancy rates in earlier years of the dataset and underpredicted them in later years (Fig. 5a). Translating PCB effects to population dynamics, the TKTD model accurately predicted temporal changes in the total population size, albeit with a time-lag in the population decline in the 1960s and 1970s (Fig. 5b). Comparing cohorts, the model showed that fertility rates were lowest in the first and most highly exposed cohort of 1966 and improved thereafter (Fig. 5c). In all cohorts, fertility increased with age, with the exception of the 1966 cohort, where fertility dropped between age 7 and 11 before increasing again. The 1986 cohort eventually reached the maximum fertility rate of an exposed population in its mature females. Survival rates were predicted to be quite low in all cohorts for the youngest age classes and increased substantially in juveniles and adults (Fig. 5d). Not surprisingly, survival was lowest in early cohorts that were exposed to the highest historical levels of PCBs through their diet.

### Time to Removal Analysis

**Fig. 6:**
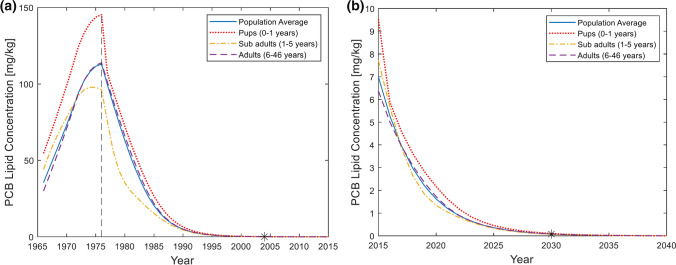
Scenarios with initial historical prey PCB exposure levels, followed by zero prey PCB exposure. PCB levels are average sum-PCB lipid concentrations for all age classes according to the TKTD population model. **a** PCB exposure stops at the peak sum-PCB concentration in seals (113 mg/kg) at year 1976 (indicated by vertical line). **b** PCB exposure stops at year 2015 (with a sum-PCB concentration of 7 mg/kg in seals)

We modelled PCB dynamics in Baltic grey seals under theoretical scenarios of complete PCB elimination in the environment to assess the importance of vertical intergenerational transfer on long-term contaminant trends (Fig. 6). Assuming PCB exposure stopped at peak levels in 1976 when sum-PCB concentration in seals reached on average 113 mg/kg (according to model), simulations revealed that concentrations in the seal population reached below 0.1 mg/kg (near zero) after 28 years, corresponding to a yearly decline of 23% (Fig. 6a). In a similar exercise, assuming that PCB exposure stopped at the 2015 levels of 7 mg/kg (according to model), simulations found that PCB concentrations reached near zero after 15 years, corresponding to a yearly decline of 26% (Fig. 6b).

### Sensitivity Analysis

**Fig. 7:**
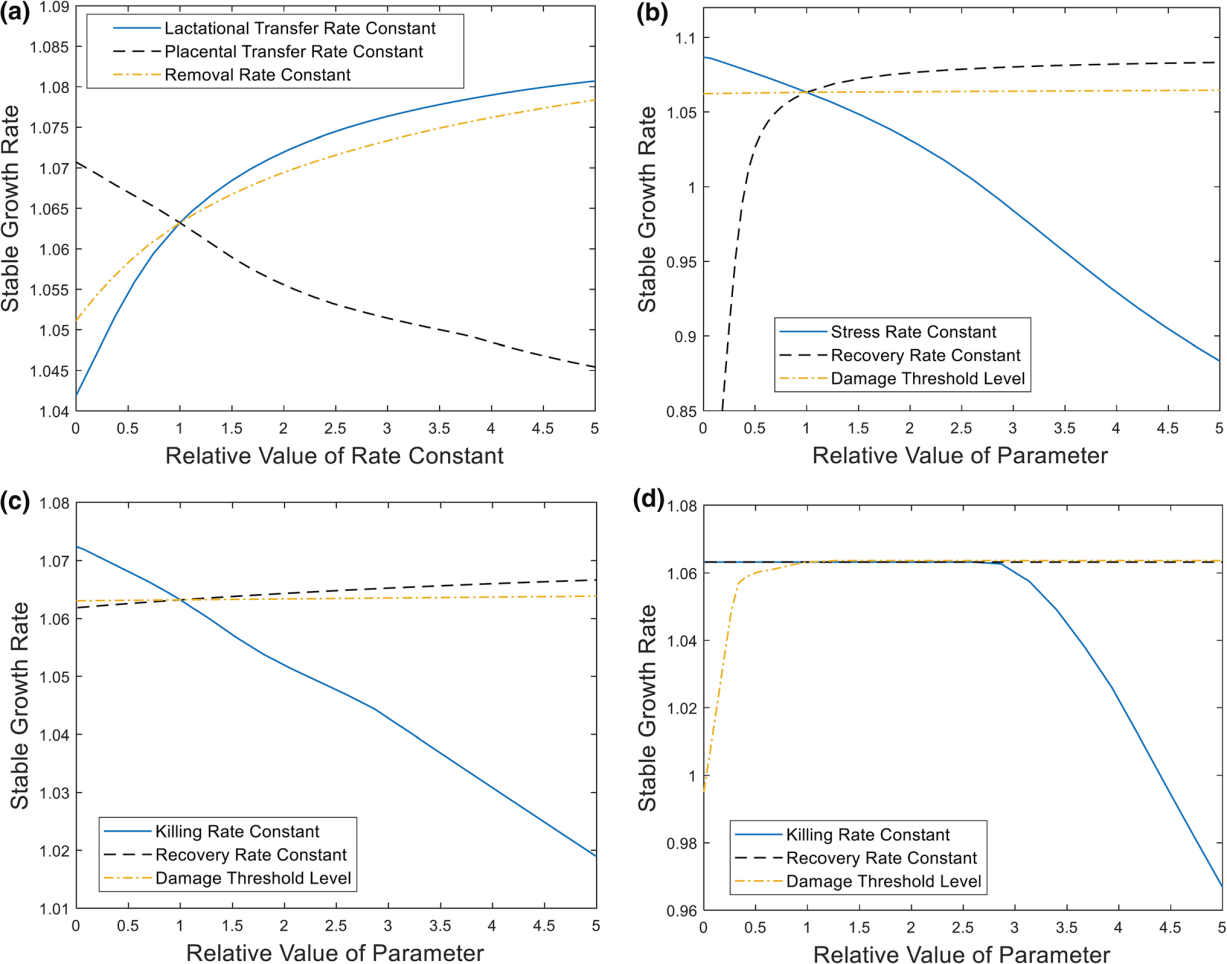
Sensitivity analysis of TKTD parameters on the stable population growth rate after long-term exposure to constant mean prey sum-PCB concentration (2 mg/kg lw). **a** Effect of TK parameters. **b** Effect of TD parameters related to reproductive stress. **c** Effect of TD parameters related to foetal survival. **d** Effect of TD parameters related to pup and female survival (*i* ≥ 1)

A sensitivity analysis was carried out to evaluate the impact of TK and TD variable uncertainty on the stable population growth rate ($\lambda$) of Baltic grey seals (Fig. 7). For the TK parameters, the sensitivity analysis showed that $\lambda$ decreased with increased placental PCB transfer from female to embryo during gestation, whereas it increased with increased milk PCB transfer from female to pup during lactation (Fig. 7a). The $\lambda$ also increased with increased PCB removal (Fig. 7a).

The effect of TD parameter variability was estimated for female reproductive stress parameters (Fig. 7b), foetal survival parameters (Fig. 7c), and pup/female survival (Fig. 7d). For reproductive stress, $\lambda$ was maximal $(\lambda =1.09$) when the stress rate constant was zero and decreased with increased values of the stress rate constant. $\lambda$ initially increased rapidly with increased recovery rate constants, but then approached maximum growth, corresponding to a state where cumulated damage was immediately recovered. $\lambda$ increased very slowly with increasing damage threshold levels until the max $\lambda$ was reached. For fetal survival, $\lambda$ decreased with increased killing rate constants and increased slowly with both increased recovery rate constants and increased damage threshold levels. For survival in all other age classes, $\lambda$ was initially unaffected by increased killing rate constants since the damage threshold level was not exceeded, though eventually decreased with greater killing rate constants. $\lambda$ initially increased rapidly with increased damage threshold levels, but when cumulated damage exceeded threshold levels in all age classes, the increase reached a plateau. Finally, $\lambda$ was unaffected by changes in the recovery rate constant since the damage threshold level was not exceeded.

## Discussion

We developed a toxicokinetic model for dietary uptake, elimination, and vertical transfer of PCBs by grey seals. This toxicokinetic model was in turn linked to a toxicodynamic model in order to estimate the resulting adverse effects on grey seal fertility and survival rates. Finally, the toxicodynamic model was linked to an age-structured Leslie matrix population model with the aim to estimate population responses from different pollutant exposures. This three-step process is a toxicokinetic–toxicodynamic population model and we term it a “TKTD population model”. Most models for adverse effects of toxicants on populations are based on empirically obtained dose–response relationships, instead of some underlying theory for toxicity (such as DEBtox or TDM). The new model is the first to combine mechanistic descriptions of both toxicokinetics and toxicodynamics and link them to effects at the population level for a marine mammal, applying the Leslie matrix approach which facilitates computation compared to earlier work on modelling effects from PCB exposure in marine mammals by individual based models (Hall et al. 2006a, Hall et al. 2006b, Hall 2013a, b, Desforges et al. 2018). The TKTD population model also captures vertical toxicant transfer between age classes, which enables detailed predictions of toxicant concentrations in animals of different age classes over long time periods, avoiding the computer-power-consuming computations of an individual-based model (IBM).

We explored how different constant exposure concentrations affect population growth rate. Steady-state analyses can be used to theoretically analyse how large contaminant concentrations a population can resist over time. According to the model, the Baltic grey seal population eventually crashes if the mean prey sum-PCB lipid concentration is 4 mg/kg or more. This level was found in Baltic fish in the early eighties (Fig. 4a). The level peaked at 9.4 mg/kg in the early seventies and if this level had remained, the population is predicted to have gone extinct 20 years later.

### Sensitivity Analysis

Our sensitivity analysis of model parameters showed that the population growth rate increased with increased vertical PCB transfer from mothers to pups during lactation (Fig. 7a). Increased vertical transfer decreases female PCB body burdens and increases female survival and fertility, while it increases pup PCB body burdens and decreases pup survival. The result is a net positive effect on population growth. On the contrary, population growth rate decreased with increased placental PCB transfer from mothers to foetuses during gestation (Fig. 7a). This result is due to the fact that placental transfer has a minor role in female detoxification, whereas foetuses are small and sensitive to PCB burden. Obviously, population growth rate increased with increased PCB removal (metabolic transformation and faecal egestion) (Fig. 7a). The model does not, however, account for the fact that some metabolites may be more toxic than the parent substance (Eisler and Belisle 1996).

Among the toxicokinetic rate constants considered in the sensitivity analysis (${k}_{\mathrm{L},i}, {k}_{\mathrm{P},i},{k}_{\mathrm{R},i}$), the model was most sensitive to changes in lactational transfer rate constants (Fig. 7a), but estimations of vertical transfer rate constants are based on detailed empirical data for grey seals (Lang et al. 2011; Berghe et al. 2012) and are probably quite valid. The estimation of removal rate constants is more uncertain. The sum-PCB removal rate constant for arctic ringed seals (Hickie et al. 2005) was adopted for 60 kg grey seals; ${k}_{\mathrm{R},\mathrm{RS}}=0.17/\mathrm{year}$. In their bioaccumulation model, Hickie et al. (2005) calculated estimations of removal rate constants for different PCB congeners, ranging from 0.03/year to 2.5/year. Changes of ${k}_{\mathrm{R},\mathrm{RS}}$ within this span (corresponding to relative values $0.18\le {k}_{\mathrm{rel}}\le 15$) yield non-negligible effects on stable growth rate (Fig. 7a). With ${k}_{\mathrm{rel}}=5$, stable growth rate is $\lambda =1.078$, to be compared with $\lambda =1.063$ for ${k}_{\mathrm{rel}}=1$. However, since good agreement was obtained between model predictions and observed PCB levels in Baltic grey seal juveniles (Fig. 4c), the estimation of removal rate constants is likely valid. The sensitivity analysis showed that lactational transfer rate constants ${k}_{\mathrm{L},i}$, placental transfer rate constants ${k}_{\mathrm{P},i}$ and removal rate constants ${k}_{\mathrm{R},i}$ are all key parameters for capturing the toxicokinetics. To describe the toxicodynamics, realistic values of the stress/killing rate constants are essential (Fig. 7b–d).

### Toxicokinetic Model Outputs

Predicted seal/prey PCB concentration ratios in year 2001 (ranging from 15 to 19), were lower than the PCB biomagnification factor (59 ± 40), calculated by Routti et al. (2005). The model also predicted lower PCB concentrations in seals during 1966–2015 compared to data (Fig. 4b). These findings are expected, since the TKTD population model only includes females, whereas Routti et al. (2005) and Segerstedt (2019) based their results on data for both sexes. Males accumulate PCBs throughout their lifetime, whereas female PCB levels stabilize over time due to vertical transfer. Hence, studies including males generally report higher mean PCB concentrations. Better agreement between model and data is expected for PCB concentrations in juveniles (0–3 years old), which have not yet transferred PCBs to offspring. Sex difference between juveniles of the same age is primarily a matter of body weight and similar PCB concentrations can be expected. Accordingly, temporal PCB concentrations predicted by the model agree well with empirical data for juveniles (Fig. 4c).

The TKTD population model links toxicokinetics to toxicodynamics, accounting for adverse effects of PCB on fertility and survival rates. An age structure different from that of a healthy population is then obtained. Since toxicokinetics differ between age classes and vertical transfer from mother to offspring is an important mechanism, toxicokinetics on the population level is altered by the toxicodynamics. Some model outputs were compared with and without toxicodynamic influence, including biomagnification factors (Fig. 3a) and PCB concentrations (Fig. 4b–c). When toxicodynamic effects were included, higher PCB accumulation was predicted. Moreover, the biomagnification factor increased with prey PCB concentration. These results are primarily explained by changes in vertical transfer. Fertility rates decrease with increasing PCB exposure. Since PCB transfer from females to offspring then decreases, bioaccumulation in females increases. Since pup survival is considerably lower than adult survival (SI-Table 4), PCBs are more effectively eliminated from the population (through vertical transfer) when fertilities are high.

### Toxicodynamic Model Outputs

Predicted population sizes started to decline later than the observed historical trend (Fig. 5b). This is expected since the historic population size was initially greatly reduced by hunting and by-catches (Harding and Härkönen 1999), effects not included in the TKTD population model. Inclusion of density dependence yields predictions with minimum levels closer to historical data, but also slower recovery during later years, where curves depart from the historical trend. Values on density parameters affecting adult survival rates may have been put too high in relation to density parameters affecting fertilities and pup survival. Accurate relative adjustments of density parameters, without changing the carrying capacity, would probably give a more realistic population recovery and a bottom population size slightly above historical data. However, with inclusion of hunting and by-catches, better agreement is expected.

### Model Assumptions and Limitations

#### Population-Based Model

The TKTD population model is a population-based model (PBM). More details could have been included if an individual-based model (IBM) had been adopted, but the simplicity of a PBM allows easier interpretation of model outputs and much faster computation. It is also possible to extend the TKTD population model with more details, such as using a multi-compartment model to describe toxicokinetics.

In PBMs based on mechanisms at the individual level, the *pooling effect* occurs (Klanjscek et al. 2006). Properties are expressed as mean values for different age classes, but a real population has individual variation within each age class. Some individuals may have considerably higher toxicant and hazard/stress levels than model predictions. In IBMs, individuals with a high level of cumulated hazard/stress die or become sterile before they have acquired enough energy to reproduce. In PBMs, negative effects on fertility are averaged over many individuals and the energy for reproduction is pooled from all adults without losses. Hence, fertility is overestimated and extinction risks may be underestimated in population viability analyses.

#### Measurement of PCB Body Burden

Since PCBs mainly accumulate in the blubber of marine mammals, it was assumed that blubber concentration could be used as an index for total body burden. A simple approach for conversion between total body concentration and lipid concentration was adopted. This may not always be accurate since lipophilic contaminants are slowly excreted, whereas blubber thickness can change fast. An emaciated animal with a thin blubber layer may have a very high blubber PCB concentration, although the total body burden is similar to that of an animal in normal nutritional condition (Bergman 2007). However, conversion of concentrations was only used for comparison with empirical data, primarily collected in the autumn when blubber layers are thick.

The composition of fish species in the Baltic Sea have changed throughout time (HELCOM 2018b). The simulations performed here are based on only three prey species and fixed prey preference indices for each age class. However, it might be of interest to model temporal changes in prey composition since different fish species are associated with different contaminant loads.

#### Removal Rate Constants

Kleiber’s law for allometric scaling of metabolic rate (Kleiber 1962) was used to scale removal rate constants (accounting for metabolic transformation and faecal egestion) from ringed seals to grey seals and between age classes of grey seals. Since grey seals are larger than ringed seals, they have a lower mass-specific metabolic rate and should thus have a lower capacity to eliminate PCBs per unit of body mass. This is empirically supported by some publications. From collected samples, Routti et al. (2005) found that bioaccumulation was higher in Baltic grey seals than in Baltic ringed seals, indicating differences in the metabolic system. Routti et al. (2005) also showed that biomagnification of PCBs in Baltic seals was very high compared to Arctic seals. They suggested that Baltic seals have higher capacity to metabolize PCBs due to increased expression of the metabolizing enzymes, a response to the heavy contaminant burden in the Baltic Sea (Routti et al. 2005). On the contrary, other studies indicate that the induction capacity of the important xenobiotic metabolising enzyme CYP1A is better in grey seals than in ringed seals (Nyman 2000). Furthermore, there are indications that grey seals have a specific mechanism for metabolism of the very toxic congener IUPAC 118 (Roots and Talvari 1997). Obviously, the adopted method of scaling removal rate constants between seal species may be further elaborated with appropriate empirical evidence.

#### Toxicodynamic Model

The adopted toxicodynamic model is based on the threshold damage model (TDM), including damage cumulation, damage threshold levels, and recovery. Uterine occlusions in Baltic grey seal females occurred at earliest in 7-year-old females, whereas uterine leiomyoma usually appeared after 15 years of age (Bergman 2007). Leiomyoma prevalence increased with lifetime exposure (Bredhult et al. 2008). These findings support the application of our model, where damage cumulates over time and adverse effects occur when threshold levels are reached. Furthermore, grey seal females can probably recover from sterility caused by PCB exposure when PCB concentrations decrease, at least to some extent. PCBs also cause adverse effects on the immune system of grey seals, reducing survival by increased maintenance costs and reduced available energy for dealing with stressors, such as hook worms and other pathogens (Klanjscek et al. 2007). These immune effects were not included in the current model design.

#### Parameterization of the Toxicodynamic Model

Reproductive stress parameters were calibrated by fitting predicted pregnancy rates to observed pregnancy rates in Baltic grey seals (Fig. 5a). However, the only negative effect on pregnancy rates in the model is PCB exposure, whereas observed pregnancy rates may have been affected also by other contaminants (like DDT) or other factors, possibly in interaction with each other. Despite parameter adjustments, the model for negative effect of reproductive stress on fertility could not generate the fast reduction in pregnancy rate seen in empirical data between mid-60 s and mid-70 s.

No DEBtox or TDM parameters for grey seals affected by PCBs are currently available in the literature, but a DEB model for mink, accounting for adverse effects of PCBs on fecundity and kit survival, has previously been developed (Desforges et al. 2017). Since there is a strong correlation between mass-specific metabolic rate and sensitivity to a toxicant (Baas and Kooijman 2015), we consider it wise to apply Kleiber’s law to perform allometric scaling of some TDM parameters. We suggest ways to translate DEBtox parameters between species and relate them to TDM parameters in SI. The parameter values adopted for the TKTD population model should be considered as a starting point for future model refinements.

### Possible Model Extensions

#### Multi-Compartment Model

In the model, total body PCB concentration governs cumulation of damage. In real animals, PCBs are primarily stored in the blubber and toxic effects are primarily governed by the concentration in the blood (Klanjscek et al. 2007). In the model, blubber thickness is a specified fraction of the body weight, whereas blubber thickness changes during the year and is density dependent. An age-specific model for annual variation of blubber weight is a simple addition that can be immediately implemented to the current model without modification of equations. It is also possible to describe the toxicokinetics by a *multi-compartment model* that differentiates bioaccumulation in different tissues and link them through diffusion rates. One alternative is a *two-compartment model*, dividing body mass into *structure* and *reserve* (blubber), where structure follows von Bertalanffy growth and reserve is built up and consumed throughout the year. PCB is stored in reserve when reserve is built up and transferred to structure when reserve is consumed. Damage cumulates when the structural PCB concentration is high, which is more in line with adverse effects of PCBs being linked to blood concentrations (Klanjscek et al. 2007). The model may account for larger reserve build-up during years of abundant prey. It may be interesting to investigate how temporal fluxes in fish abundance result in varying blubber thickness and translate into PCB-induced damage different years.

#### Combination of Risk Factors

Besides PCBs there may be other contaminants with adverse effects on fertility and survival rates, possibly in interaction. DEBtox models and TDMs allow inclusion of combined effects of multiple toxicants. The cumulated damage from the mixture of all toxicants may be calculated as the sum of the internal damages from all toxicants separately (Ashauer et al. 2007a). However, this effect is only additive. There are also ways of accounting for interaction between different compounds, such as using nonlinear stress functions in DEBtox models (Kooijman 2000). Hunting, by-catches and decreasing ice coverage (due to climate change) may also be relevant aspects to consider in population viability analyses of Baltic grey seals. The population is currently faced with a sharp increase in hunting quotas and also vulnerable to declining ice fields due to global warming in addition to prevailing high levels of POPs. It will be important to elaborate scenarios with all these stressors in combination (Cervin et al. 2020; Silva et al. 2021) in order to avoid a new era of overexploitation.

#### Application to Other Marine Mammals

The developed model can be extended to other marine mammals than seals and other lipophilic contaminants than PCBs by adjustments of parameter values. Key parameters that have to be identified are at least three toxicokinetic parameters; a lactational transfer rate constant ${k}_{\mathrm{L}}$, a placental transfer rate constant ${k}_{\mathrm{P}}$ and a removal rate constant ${k}_{\mathrm{R}}$. Parameters ${k}_{\mathrm{L}}$ and ${k}_{\mathrm{P}}$ depend on species-specific nutrient transfer rates between female and offspring and the lipophilicity of the contaminant, whereas ${k}_{\mathrm{R}}$ depends on the species’ metabolism and toxicant properties. To capture the toxicodynamics, stress/killing rate constants are essential to estimate. These depend on the physical modes of action (reproductive stress, foetal survival and pup/female survival) for the current species and toxicant. Similar values of killing/stress rate constants may, however, be used for similar species exposed to the same toxicant, whereas damage threshold levels may be allometrically scaled between species (see SI). Other parameters, critical to the species of interest, are some life history parameters; fertilities and survival rates for a healthy population, lactation and gestation period length ($\Delta {\tau }_{l}$ and $\Delta {\tau }_{g}$), sexual maturation age and adult body mass. Adult food consumption rate is required as well. Allometric scaling may be applied to derive parameter values for different age classes based on body mass and von Bertalanffy growth. A simplified model containing a few essential parameter estimates may be rather easily acquired by combining species and contaminant knowledge with data transformation from other species. Despite large simplifications, the model may be very useful.

## Supplementary Information

Below is the link to the electronic supplementary material.

Supplementary file1 (PDF 1211 KB)
