# Supplementary material for: Maternal Transfer and Long-Term Population Effects of PCBs in Baltic Grey Seals Using a New Toxicokinetic–Toxicodynamic Population Model
Source: Arch Environ Contam Toxicol. 2022 Oct 15;83(4):376–94. doi: 10.1007/s00244-022-00962-3 (PMC9637078; doi:10.1007/s00244-022-00962-3)
Supplement: Supplementary file 1 — Supplementary file1 (PDF 1211 KB) [file 244_2022_962_MOESM1_ESM.pdf]

Supplemental Information for

Maternal transfer and long-term population effects of PCBs in Baltic grey seals using a new toxicokinetic-toxicodynamic population model

Karl Mauritsson, Jean-Pierre Desforges, Karin C. Harding

Table of Contents

1. Model Overview and Calculation Scheme 1
2. Toxicokinetic Model 4
  2.1 Growth model 5
  2.2 Bioaccumulation model 7
    2.2.1. Bioaccumulation in females 7
    2.2.2. Bioaccumulation in Fetuses 11
    2.2.3. Bioaccumulation in Pups 12
3. Toxicodynamic Model 14
  3.1 Cumulation of Damage, Hazard and Stress 16
  3.2 Fertilities and Survival Rates 17
4. Population Model 18
  4.1 Leslie matrix model 18
  4.2 Density dependence 18
5. Model Variables and Parameters 19
  5.1 Baltic grey seal biology and ecology 20
    5.1.1. Grey seal growth and life history parameters 20
    5.1.2. Grey seal diet variables and parameters 21
    5.1.3. Conversion of PCB concentrations 22
    5.1.4. Grey seal population parameters 23
  5.2 Toxicokinetics 24
    5.2.1. Assimilation efficiencies 24
    5.2.2 Vertical transfer rate constants 24
    5.2.3. Removal rate constants 25
  5.3 Toxicodynamics 26
    5.3.1 Calibration of Reproductive Stress Parameters 26
    5.3.2. Estimation of Toxicodynamic Model Parameters Related to Survival 28
    5.3.3. Transformation of DEBtox parameters 29
6. Model Simulations 31
  6.1 Steady state 31
  6.2 Sensitivity analysis 31
References 32

1. Model Overview and Calculation Scheme

Our matrix model, called the TKTD population model, analyses adverse effects of PCBs on Baltic grey seals by linking toxicokinetics (TK) of dietary and vertically transferred PCB accumulation to toxicodynamics (TD) of adverse effects on reproduction and survival that ultimately predict changes to population demographics and growth rates. Survival and fecundity parameters of an age-structured Leslie matrix model for an ideal Baltic grey seal population are modified due to internal PCB concentrations following DEBtox and TDM principles of damage, hazard, stress, and recovery.

While the model runs on an annual basis for the population dynamics, each year is divided into three periods (*lactation, delay* and *gestation*), representing different lifehistory phases for a reproducing grey seal female (Fig. 1). The year starts with the *lactation period* (lasting for 18 days), during which pups are nursed by their mothers. After weaning, females mate and the *delay period* starts (lasting for 100 days), after which the fertilized egg is implanted. During the *gestation period* (lasting for 247 days), the fetus develops in the uterus. The year ends with the birth of new pups. Since all deaths are assumed to occur just before the birth events, all pups that are born have mothers that nurse them. Different TD effects of PCBs occur after specific time periods (e.g., some fertility effects are estimated after the delay period).

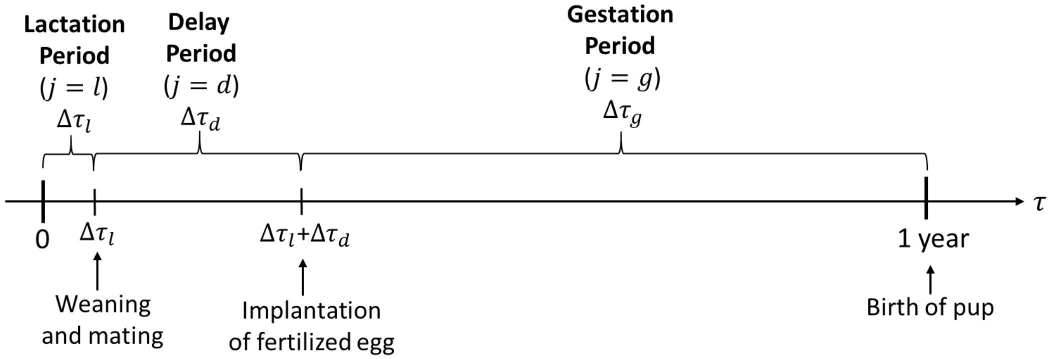

Fig. 1 Division of a year into three periods, representing the reproduction cycle of grey seal females. $\Delta\tau_l = 18$ days, $\Delta\tau_d = 100$ days, $\Delta\tau_g = 247$ days.

The TKTD population model consist of three interconnected sub models (Fig. 2). The toxicokinetic (TK) model of PCB accumulation follows the total body PCB concentrations in seals of different age classes at the end of each time period of a year. The toxicodynamic (TD) model follows damage, hazard and reproductive stress in seals of different age classes at the end of each time period of a year. The Leslie matrix model follows number of individuals, survival rate and fertility in each age class at the end of each year. The TKTD population model was implemented as a set of function and script files in the numerical software MATLAB® (MathWorks Inc., Natick, MA, USA) and all analyses were performed with these.

Before any calculations can be performed, a number of initial conditions have to be specified. These are initial PCB concentrations, initial damage levels and initial population vector (Table 1). Also, PCB concentrations in different prey are specified for each year of the simulation (Fig. 8). Thereafter, PCB concentrations and damage levels in fetuses, pups and females at the end of the different time periods within a year are successively calculated. Hazard/stress levels are calculated based on obtained damage levels and fertilities and survival rates are updated based on these. A new population vector is finally calculated, including the number of individuals in different age classes at the end of the year. Outputs from calculations for one year are used as inputs to calculations for the next year. The procedure is repeated for one year at a time and may be continued for as many years as desired (Fig. 2).

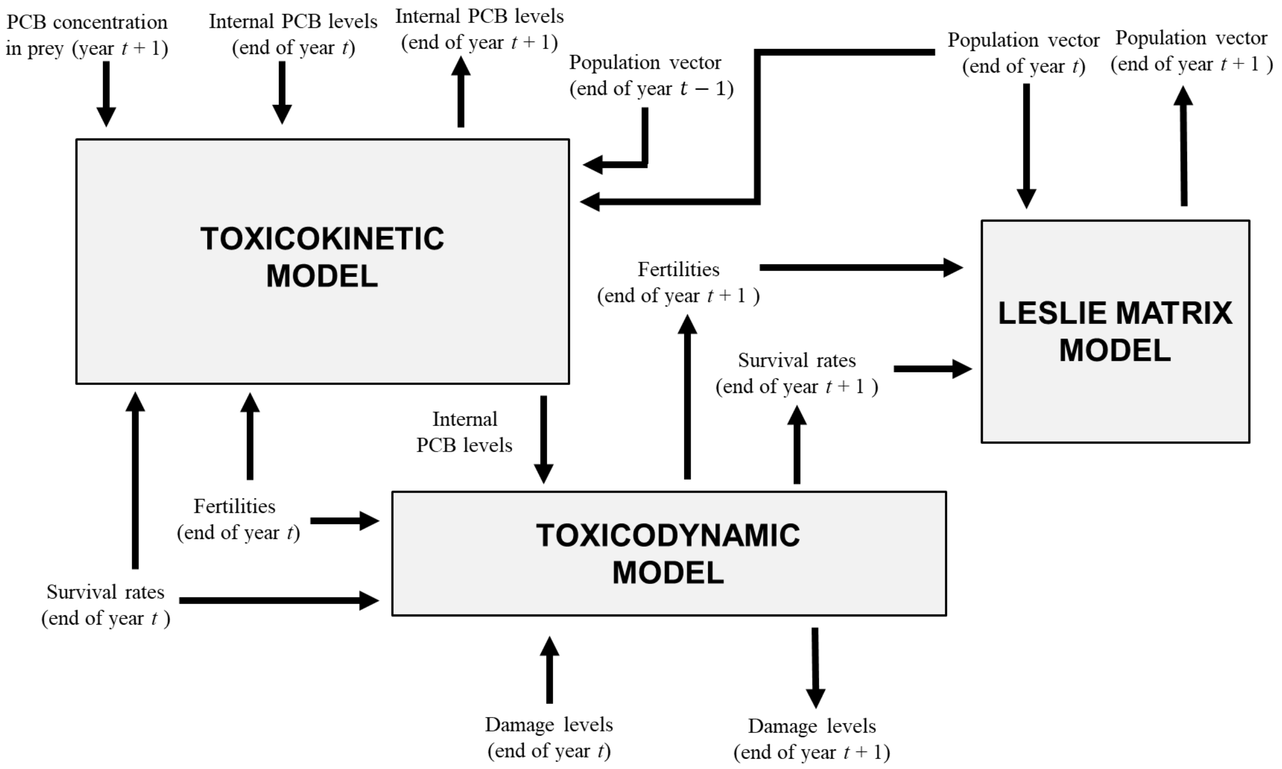

Fig. 2 Overview of the TKTD population model. Text boxes represent sub-models, performing calculations for year $t + 1$. Free texts are inputs and outputs, with arrows showing relations to sub-models.

For each year, the following calculations are performed:

1. Calculation of **PCB levels** in pups and females at the end of the **lactation period**, as a result of vertical transfer from females to pups, based on PCB levels in fetuses, pups and females at the end of previous year. Transfer levels are dependent on fertilities, determining the mean number of pups per female.

2. Calculation of cumulated **damage levels** in pups and females at the end of the **lactation period**, based on PCB and damage levels in fetuses, pups and females at the end of previous year.

3. Calculation of accumulated **PCB levels** in pups and females at the end of the **delay period**, based on PCB levels at the end of the lactation period. PCB is assimilated through dietary uptake and eliminated through metabolic transformation and fecal egestion.

4. Calculation of cumulated **damage levels** in pups and females at the end of the **delay period**, based on PCB and damage levels in pups and females at the end of the lactation period.

5. Calculation of **reproductive stress levels** in females at the end of the **delay period**, based on reproductive damage levels at the end of the lactation period.

6. Calculation of reduced **fertilities** at the end of the **delay period**, based on ideal fertilities and female reproductive stress levels at the end of the delay period.

7. Calculation of **PCB levels** in fetuses, pups and females at the end of the **gestation period**, as a result of bioaccumulation and placental transfer from females to fetuses, based on PCB levels in pups and females at the end of the delay period. Transfer levels are dependent on fertilities at the end of the delay period, determining the mean number of produced fetuses per female.

8. Calculation of cumulated damage **levels** in fetuses, pups and females at the end of the **gestation period**, based on PCB and damage levels in pups and females at the end of the delay period.

9. Calculation of hazard/stress levels in fetuses, pups and females at the end of the **gestation period**, based on damage levels in pups and females at the end of the delay period.

10. Calculation of reduced **survival rates** at the **end of the year**, based on ideal survival rates and hazard levels in pups and females at the end of the gestation period.

11. Calculation of reduced **fertilities** at the **end of the year**, based on fertilities at the end of the delay period (reduced due to reproductive stress) and fetal hazard levels at the end of the gestation period.

12. Calculation of new **population vector** (accounting for population growth during one year), based on updated fertilities and survival rates, and previous year's population vector.

In order to describe the temporal dynamics of variables for fetuses, pups and females of different age classes at different times τ within a year, we use the notation $c_{k,i}^{j}(\tau)$. The first letter represents the variable of interest, here being seal PCB concentration in mg/kg. The upper index j indicates the time period (l = lactation, d = delay, g = gestation). The first lower index k indicates if it is a fetus ($k = 0$), a pup ($k = 1$) or a female ($k = i$), where $i = 2, \dots, 46$ indicates the age class. Individuals of age class $i = 1$ (with an age of 0-1 year) are here referred to as *pups*, whereas individuals of age class $i = 2, \dots, 46$ are referred to as *females*. For fetuses and pups, the second lower index indicates the age class of the mother. Hence, $c_{0,i}^{j}(\tau)$, $c_{1,i}^{j}(\tau)$ and $c_{i,i}^{j}(\tau)$ are PCB concentrations at time τ during time period j for fetuses with mothers of age class i, pups with mothers of age class i and females of age class i, respectively. The temporal dynamics is described by differential equations. By solving these, state variables at the end of time periods within a year are calculated. These are used to perform step-wise calculations from one time period to the next and also from one year to the next, using obtained variable values as initial conditions for the next calculation. In order to capture variable states at the end of different time periods j for specified years t, we also introduce the notations $\hat{c}_{0,\mathrm{i}}^{j}(t)$, $\hat{c}_{1,\mathrm{i}}^{j}(t)$ and $\hat{c}_{i}^{j}(t)$, where a hat over a variable name indicates reference to the end of a time period. For example, $\hat{c}_{1,i}^{l}(t)$ are PCB concentrations at the end of the lactation period (l) of year t in pups with mothers of age class i. The same conventions are used also for other variables (damages, hazards and stresses). Furthermore, we use the notation where a dot above letters indicates a rate with respect to time and a straight overline indicates an average value. The state variables of the TKTD model are listed in Table 1.

2. Toxicokinetic Model

The toxicokinetic model describes PCB bioaccumulation in pups and females through dietary uptake and elimination as well as vertical transfer from mother to fetus through the placenta during gestation and from mother to pup through breast milk during lactation (Fig. 3).

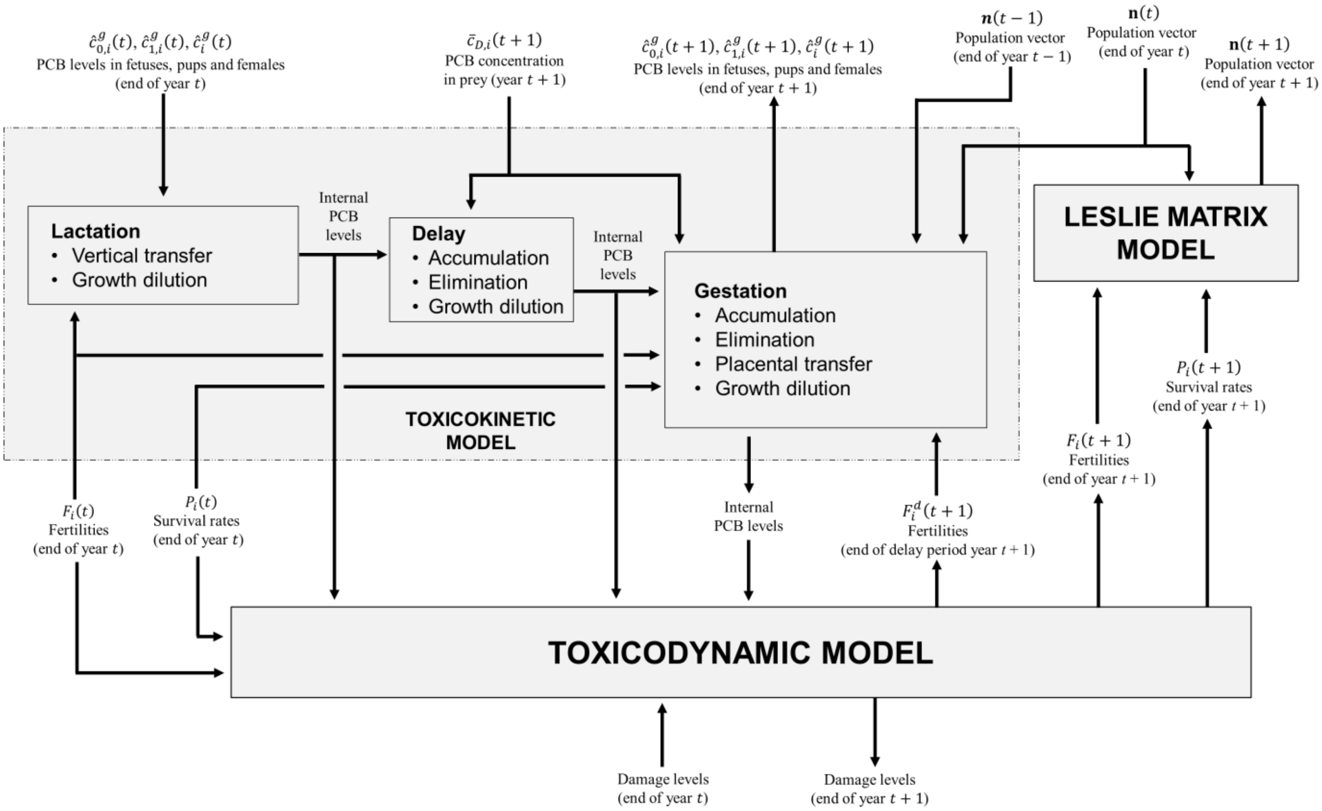

Fig. 3 The toxicokinetic model and its connections to other sub models. Text boxes represent sub modules. Free texts are inputs and outputs, with arrows showing relations to sub modules. The flowchart illustrates computations performed for year $t+1$, based on inputs from previous year t.

During the *lactation period*, PCB is transferred from females to pups through the breast milk. Neither females nor pups consume prey during this period. Hence, no PCB is accumulated from the environment, but it is redistributed from females to pups through vertical transfer, including some losses to the environment. PCB concentrations also change due to growth dilution, especially in the fast growing pups. During the *delay period*, both females and pups feed on prey. Hence, PCB concentrations change due to dietary uptake, elimination (fecal egestion and metabolic transformation) and growth dilution. During the *gestation period*, females and pups continue to feed, but females also transfer PCB to fetuses through the placenta. Hence, concentration changes in pups are governed by dietary uptake, elimination, and growth dilution, whereas concentration changes in females are governed by dietary uptake, elimination, growth dilution and vertical transfer. Furthermore, the PCB concentration increases in fetuses due to vertical transfer, which affects the PCB concentration in new-born pups at the start of the next year.

2.1 Growth model

In order to calculate growth dilution rates and keep track of body weights (affecting rates of feeding, vertical transfer and PCB elimination), a sub model for body growth was established (Fig. 4).

Fetuses are assumed to growth exponentially during the gestation period (Peters 1983). Hence, the body weight of a fetus at time τ during a year is obtained as:

$$W_0(\tau) = W_{00} e^{g_0(\tau-(\Delta\tau_l+\Delta\tau_d))}, \qquad \Delta\tau_l + \Delta\tau_d \leq \tau \leq 1 \text{ yr} \tag{1}$$

Here, W_{00} is the initial fetal weight (the weight of a fertilized egg) and g_0 is an exponential growth rate constant. At the end of the gestation period, the fetus has reached the birth weight W_{birth}. Hence, the initial fetal weight W_{00} is calculated as:

$$W_{00} = W_{birth} e^{-g_0 \Delta\tau_g} \tag{2}$$

For simplicity it is assumed that new-born pups continue to grow exponentially with the same growth rate as fetuses during the lactation period. The body weight of a pup at time τ during the lactation period is obtained as:

$$W_1(\tau) = W_{00} e^{g_0(\Delta\tau_g+\tau)}, \quad 0 \leq \tau < \Delta\tau_l \tag{3}$$

At the end of the lactation period, the pup has reached the weaning weight; $W_1(\Delta\tau_l) = W_{wean}$. Hence, the exponential growth rate constant g_0 is calculated as:

$$g_0 = ln(W_{wean}/W_{birth})/\Delta\tau_l \tag{4}$$

After weaning, grey seal pups typically lose weight during some months due to reduced nutritional intake (Boyd 1984), but when they become more skilled hunters they start to regain weight. For simplicity, it is here assumed that pups keep the weaning weight until they reach sub adult age $\tau_{SA} = 1$ yr.

Grey seal females are typically full-grown at an age of six years. When they nurse pups (during the lactation period) they lose a considerable amount of weight, but this is regained when they feed during the remaining part of the year. Under constant environmental conditions, dynamic energy budget (DEB) theory predicts body growth according to the von Bertalanffy growth function (Kooijman 2000), in which the body weight $W(\tau)$ at age τ is obtained from the equation:

$$\left(\frac{W(\tau)}{W_\infty}\right)^{1/4} = 1 - \left[1 - \left(\frac{W_0}{W_\infty}\right)^{1/4}\right] e^{-\gamma(\tau-\tau_0)} \tag{5}$$

Here, W_∞ is the asymptotic body weight, W_0 is the initial body weight (the body weight at $\tau = \tau_0$) and γ is a growth rate constant. The von Bertalanffy growth function is here adopted to describe body growth of grey seal females from weaning ($\tau = \Delta\tau_l$) up to the age of sexual maturation ($\tau_{mat} = 5$ yr). At that age, females reach the mature body weight W_{mat} and start to breed. The body weight of non-mature females ($i = 2 - 5$) at time τ during the growth period of a year is obtained as:

$$\left(\frac{W_i(\tau)}{W_\infty}\right)^{1/4} = 1 - \left[1 - \left(\frac{W_{wean}}{W_\infty}\right)^{1/4}\right] e^{-\gamma(i-2+\tau)} \quad i = 2, \ldots, 5 \quad 0 \leq \tau \leq 1 \text{ yr} \tag{6}$$

The weaning body weight W_{wean}, the mature body weight W_{mat}, the post-lactation body weights ($W_{lac,6}$ and $W_{lac,7}$) and the maximum body weight W_{max} are specified in Table 2, based on data for full-grown Baltic grey seal females (Härkönen 2016). Females lose significant weight during lactation and it is assumed that their weight decrease exponentially throughout the lactation period, from the initial body weight (W_{mat} or W_{max}) to the post-lactation body weight; $W_{lac,6}$ or $W_{lac,7}$ for primiparous females (first-time breeders) and multiparous females (experienced breeders), respectively:

$$\left.\begin{array}{l} W_6(\tau) = W_{mat} e^{-g_L\tau} \\ W_i(\tau) = W_{max} e^{-g_L\tau} \quad (i = 7, \ldots, 46) \end{array}\right\} 0 \leq \tau < \Delta\tau_l \tag{7}$$

Here, g_L is the exponential body weight decline rate constant, calculated as:

$$g_L = ln\left(W_{max}/W_{lac,7}\right)/\Delta\tau_l \quad (8)$$

After lactation, during the delay period, mature females are assumed to linearly regain the weight they lost during lactation:

$$\left.\begin{array}{l} W_6(\tau) = W_{lac,6} + k_6(\tau - \Delta\tau_l) \\ W_i(\tau) = W_{lac,7} + k_7(\tau - \Delta\tau_l) \quad (i = 7, \ldots, 46) \end{array}\right\} \quad \Delta\tau_l \le \tau < \Delta\tau_l + \Delta\tau_d \quad (9)$$

The regain rate constants (k_6 and k_7) are calculated as:

$$k_6 = \left(W_{mat}/W_{lac,6}\right)/\Delta\tau_d \quad , \quad k_7 = \left(W_{max}/W_{lac,7}\right)/\Delta\tau_d \quad (10)$$

After the delay period, mature females continue to grow according to von Bertalanffy throughout the gestation period and reach the maximum body weight W_{max} at the end of the year:

$$\left(\frac{W_i(\tau)}{W_\infty}\right)^{1/4} = 1 - \left[1 - \left(\frac{W_{wean}}{W_\infty}\right)^{1/4}\right] e^{-\gamma[4+\tau-(\Delta\tau_l+\Delta\tau_d)]} \quad i = 6, \ldots, 46 \quad \Delta\tau_l + \Delta\tau_d \le \tau \le 1 \text{ yr} \quad (11)$$

In succeeding years, females are assumed to repeat the pattern of exponential decline (lactation period), linear regain (delay period) and von Bertalanffy growth (gestation period), see Fig. 4.

The asymptotic body weight W_∞ and the von Bertalanffy growth rate constant γ were calculated from Eq. (5) with $W_0 = W_{wean}$, $\tau_0 = 1$ yr and insertion of known data; $[\tau_{mat}, W_{mat}]$ and $[\tau_{max}, W_{max}]$ (Table 2).

The mean body weight $\overline{W}_i^j$ during time period j is calculated as the integrated mean value over the period ($\tau_0^j \le \tau \le \tau_0^j + \Delta\tau_j$):

$$\overline{W}_i^j = \frac{1}{\Delta\tau_j}\int_{\tau_0^j}^{\tau_0^j+\Delta\tau_j} W_i(\tau)\mathrm{d}\tau \quad (12)$$

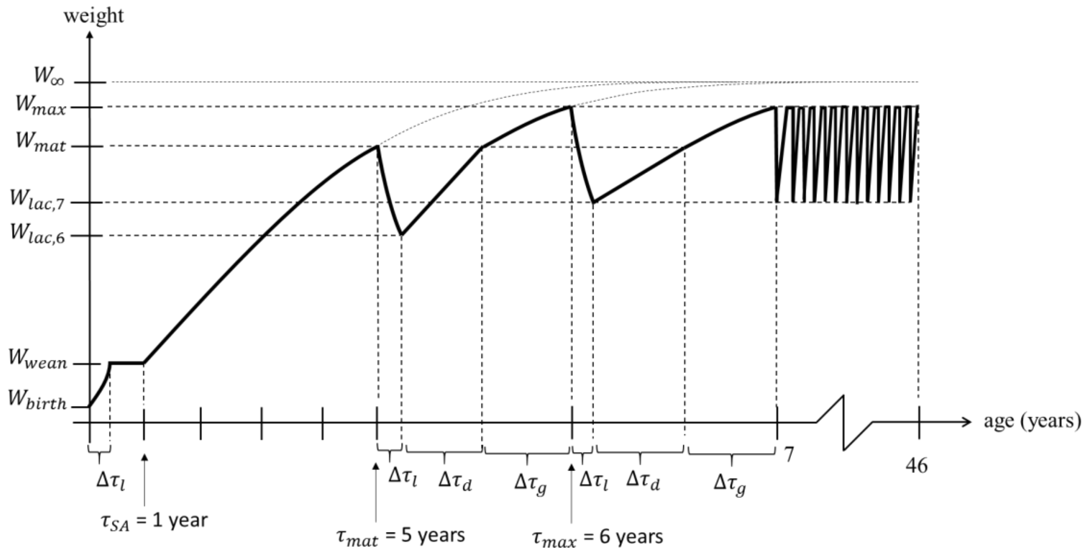

Fig. 4 Body weight as a function of age according to growth model. Pups increase their weight exponentially during lactation and keep it constant during the remainder of the year. Sub-adults follow von Bertalanffy growth function until maturation. Mature females lose weight exponentially during lactation, regain it linearly during the delay period and grow according to von Bertalanffy during gestation. The same yearly pattern for mature females is then repeated.

2.2 Bioaccumulation model

The major routes for uptake, transfer and elimination of PCB in females, pups and fetuses are illustrated in Fig. 5. The corresponding toxicokinetic equations are now treated for each groups separately.

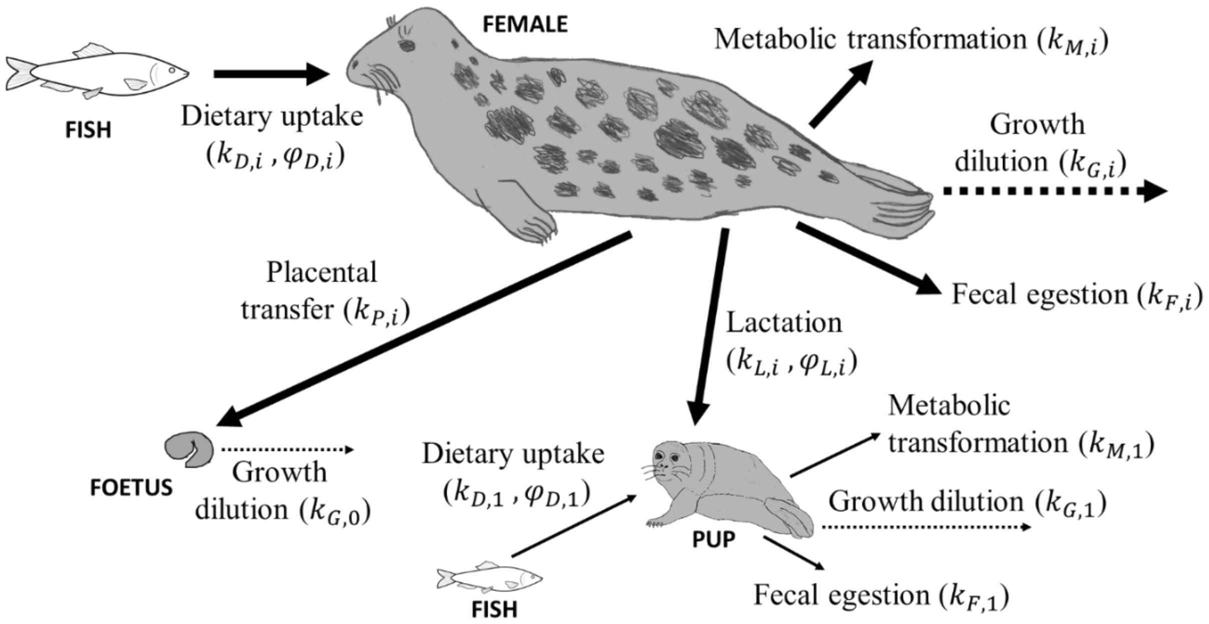

Fig. 5 Major routes for uptake, transfer and elimination of PCB in grey seals, including associated rate constants ($k_{D,i}, k_{M,i}, k_{F,i}, k_{L,i}, k_{P,i}, k_{G,i}$) and assimilation efficiencies ($\varphi_{D,i}, \varphi_{L,i}$).

2.2.1. Bioaccumulation in females

Bioaccumulation in females older than one year ($i = 2, \dots, 46$) includes dietary uptake, elimination and vertical transfer to offspring (Fig. 5). The toxicokinetics is described by a physiologically based equation similar to the one-compartment model, commonly used in DEBtox models, but extended to account also for growth dilution and vertical transfer. The rate of change of the total amount of PCB in a female is the rate by which PCB is assimilated (assumed to be proportional to the concentration in the prey) subtracted by the rate by which PCB is eliminated (assumed to be proportional to the concentration in the body):

$$\frac{\mathrm{d}}{\mathrm{d}\tau}\left(W_i c_{i,i}^j\right) = k_{D,i}^j W_i c_{D,i} - k_{E,i}^j W_i c_{i,i}^j \ , \qquad i = 2, \dots, 46 \quad , \ j = l, d, g \ , \qquad 0 \le \tau \le \Delta\tau_j \qquad (13)$$

Here, $c_{i,i}^j(\tau)$ is the total body PCB concentration (mg/kg) at time τ (yr) in a female of age class i during time period j, $c_{D,i}$ is the mean PCB concentration in the diet (mg/kg) and $W_i(\tau)$ is the female body weight (kg). The repeated notation of index i in female concentrations ($c_{i,i}^j$) is applied for technical reasons, enabling a compact general description of all age classes and fetuses. The dietary uptake rates (yr^{-1}) for females (and pups) are defined as:

$$k_{D,i}^j = \begin{cases} 0 \ , & j = l \\ \varphi_D \dot{W}_{D,i}/W_i \ , & j = d, g \end{cases} \quad , \qquad i = 1, \dots, 46 \qquad (14)$$

Here, $\dot{W}_{D,i}$ is the prey consumption rate (kg/yr). The fraction of consumed PCB that is assimilated by the seal is assumed to equal the diet assimilation efficiency φ_D, the fraction of ingested prey that is assimilated into energy. The assumption is motivated by studies indicating that the assimilation of hydrophobic substances is closely linked to digestion of associated food (Bodiguel et al. 2009). Dietary uptake rates are zero when $j = l$ since neither females nor pups feed during lactation. As a simplification, it is assumed that also non-mature females ($i = 2, \dots, 5$) fast during the lactation period, though they have no pups to nurse. The simplification will only have minor effects since the lactation period is very short (18 days) compared to a whole year. All seals are assumed to feed throughout the whole delay period, neglecting the two weeks of fasting during molting. According to DEB theory, the feeding rate of an animal in a constant environment is proportional to the surface area of the structural body volume (Kooijman 2000). By assuming this, isometric growth and body weight proportional to body volume, the prey consumption rate $\dot{W}_D$ for a seal of weight W is expressed as:

$$\dot{W}_D = \alpha_D W^{2/3} \tag{15}$$

The prey consumption factor α_D is a constant, estimated from empirical data of prey consumption in Baltic grey seals.

Mature females lactate and hence they lose and regain weight during a year. Due to their rapid weight regain after lactation, they can be expected to peek their daily prey consumption during the delay period, though they have higher weight during other periods. In a DEB context, the reserve (blubber) mass is reduced during lactation, but the structural body mass is not. The weight W in Eq. (15) is best considered as a mean body weight for females of a certain body length (which do not reduce during fasting). As a simplification, it is here assumed that the prey consumption rate for seals of age class i can be described by Eq. (15) with W as the maximum body weight of a year, i.e. the body weight at the end of the year ($\tau = 1$ yr). The age-specific dietary uptake rate constants for females (and pups) are approximated as:

$$k_{D,i}^{j} \approx \begin{cases} 0 & , \quad j = l \\ \alpha_D \varphi_D [W_i(1\text{ yr})]^{2/3} / \bar{W}_i^{j} & , \quad j = d, g \end{cases} \quad , \quad i = 1, \dots, 46 \tag{16}$$

Here, $\bar{W}_i^{j}$ is the mean body weight of individuals in age class i during time period j.

The female PCB elimination rates (yr^{-1}) accounting for all elimination routes, are defined as:

$$k_{E,i}^{j} = \begin{cases} k_{L,i} & , \quad j = l \\ k_{R,i} & , \quad j = d \\ k_{R,i} + k_{P,i} & , \quad j = g \end{cases} \quad , \quad i = 2, \dots, 46 \tag{17}$$

Elimination through vertical transfer is accounted for via the lactational transfer rate constant $k_{L,i}$ and the placental transfer rate constant $k_{P,i}$, respectively. There are two separate routes for PCB removal through fecal egestion. The first route is accounted for via the diet assimilation efficiency (φ_D), with elimination proportional to the concentration in the prey. The second route is accounted for via the fecal elimination rate constant ($k_{F,i}$), with elimination proportional to the concentration in the body. However, the contribution from the second route is probably minor compared to the first one (Bodiguel et al. 2009). For simplicity, the removal rate constant $k_{R,i}$ accounts for the combined effect of metabolic transformation and fecal elimination:

$$k_{R,i} = k_{M,i} + k_{F,i} \quad , \quad i = 1, \dots, 46 \tag{18}$$

Here, $k_{M,i}$ is the metabolic transformation rate constant and $k_{F,i}$ is the fecal elimination rate constant. The vertical transfer rate constants ($k_{L,i}$ and $k_{P,i}$) are considered as mean values for each period. This simplification neglects that vertical transfer rates increase with the weight of the offspring (fetus or pup), which grows and assimilates increasing amounts of nutrients, resulting in vertical transfer rates that increase with time.

Notice that all rate constants are considered as be age-specific, though they may have same or similar values between age classes. For the sake of generality, Eq. (13) accounts for all assimilation and elimination routes, including vertical transfer from female to offspring, though all these will not occur at the same time. When applying Eq. (13) to different time periods of a year, different rate constants appear according to Eqs. (16) and (17).

The toxicokinetic equation (Eq. (13)) can be rewritten as the following differential equation for the PCB concentration $c_{i,i}^{j}(\tau)$ at time τ in a female of age class i during time period j:

$$\frac{\mathrm{d}c_{i,i}^{j}}{\mathrm{d}\tau} + \left(k_{E,i}^{j} + k_{G,i}^{j}\right)c_{i,i}^{j} = k_{D,i}^{j}c_{D,i} \quad , \quad i = 2, \dots, 46 \ , \ j = l, d, g \tag{19}$$

The *growth dilution rate* ($k_{G,i}^{j}$), the relative increase in body weight per unit time, was here introduced:

$$k_{G,i}^{j} = \frac{1}{W_i}\frac{\mathrm{d}W_i}{\mathrm{d}\tau} \tag{20}$$

It is assumed that the mean PCB concentration in the prey is constant throughout each year; $c_{D,i}(\tau) = \bar{c}_{D,i}(t)$, where $\bar{c}_{D,i}(t)$ is the mean PCB concentration in the diet of seals in age class i during year t. Moreover, it is assumed that the growth dilution rate is approximately constant within each period of a year. The growth dilution rate ($k_{G,i}^{j}$) for seals of age class i during a time period j ($\tau_0^j \le \tau \le \tau_0^j + \Delta\tau_j$) is approximated as the mean growth dilution rate during that period:

$$k_{G,i}^{j} \approx \frac{1}{\Delta\tau_j}\int_{\tau_0^j}^{\tau_0^j+\Delta\tau_j} k_{G,i}(\tau)\mathrm{d}\tau = \frac{1}{\Delta\tau_j}\left(\ln\left[W_i\left(\tau_0^j + \Delta\tau_j\right)\right] - \ln\left[W_i\left(\tau_0^j\right)\right]\right) \tag{21}$$

During the lactation period, body weights of pups and females are described by exponential functions (Eqs. (3) and (7)). The growth dilutions rates then simplify into the exponential growth rate constants:

$$k_{G,1}^{l} = g_0 \quad , \quad k_{G,i}^{l} = -g_{\mathrm{L}} \qquad i = 6, \dots, 46 \tag{22}$$

During the rest of the year, pups have constant body weight and hence no growth dilution:

$$k_{G,1}^{j} = 0 \quad , \quad j = d, g \tag{23}$$

The effective elimination rate constant for females (and pups) during a time period j within a year is defined as:

$$\tilde{k}_{E,i}^{j} = k_{E,i}^{j} + k_{G,i}^{j} \quad , \qquad i = 1, \dots, 46 \quad , \quad j = l, d, g \tag{24}$$

Now, the toxicokinetic equation (Eq. (19)) can be approximated as the following first-order ordinary differential equation (ODE):

$$\frac{\mathrm{d}c_{i,i}^{j}}{\mathrm{d}\tau} + \tilde{k}_{E,i}^{j}c_{i,i}^{j} = k_{D,i}^{j}\bar{c}_{D,i} \quad , \qquad i = 2, \dots, 46 \quad , \quad j = l, d, g \tag{25}$$

The solution to Eq. (25) is:

$$c_{i,i}^{j}(\tau) = \kappa_{D,i}^{j}\bar{c}_{D,i} + C_i^j e^{-\tilde{k}_{E,i}^{j}\tau} \quad , \qquad \begin{cases} \kappa_{D,i}^{j} = k_{D,i}^{j}/\tilde{k}_{E,i}^{j} \\ C_i^j = c_{i,i}^{j}(0) - \kappa_{D,i}^{j}\bar{c}_{D,i} \end{cases} \tag{26}$$

Equation (26) expresses the PCB concentration in a female of age class i at time τ during period j within a year. The first term accounts for dietary uptake (proportional to dietary PCB concentration, characterized by constant $\kappa_{D,i}^{j}$) and the second term accounts for elimination (exponential decrease, characterized by elimination rate $\tilde{k}_{E,i}^{j}$). Equation (26) is used to obtain PCB concentrations in females at the end of different time periods j, based on initial PCB concentrations $c_{i,i}^{j}(0)$ at the start of the periods ($\tau = 0$). Within a year, the concentration obtained in an age class at the end of a period, is used as initial value the next period. Between years, the concentration obtained in an age class (i) at the end of a year (t), is used as initial value in next age class ($i + 1$) at the start of next year ($t + 1$).

The PCB concentration in females of age class $i+1$ at the end of the **lactation period** ($j=l,\ \tau=\Delta\tau_l$) during year $t+1$ is obtained from Eq.(26) as:

$$\hat{c}_{i+1}^{l}(t+1)=b_{i+1,i}^{l}(t+1)\hat{c}_{i}^{g}(t)\,,\qquad b_{i+1,i}^{l}(t)=e^{-\bar{k}_{E,i+1}^{l}(t)\Delta\tau_l}\quad,\quad i=1,\dots,45 \tag{27}$$

Here, $\hat{c}_i^g(t)$ is the PCB concentration in females of age class i at the end of year t. The mean elimination rate (accounting for lactational transfer and growth dilution) during the lactation period year t is:

$$\bar{k}_{E,i}^{l}(t)=\bar{k}_{L,i}^{l}(t)+\bar{k}_{G,i}^{l}\quad,\qquad i=2,\dots,46 \tag{28}$$

The mean lactational transfer rate during the lactation period year $t+1$ is calculated as:

$$\bar{k}_{L,i+1}^{l}(t+1)=2F_i(t)k_{L,i+1}\quad,\qquad i=1,\dots,45 \tag{29}$$

Here, $F_i(t)$ is the fertility of females in age class i at the end of year t. Notice that the factor $2F_i(t)$ is the mean number of pups that each female (of age class $i+1$) gave birth to at the end of previous year (t). The factor is included to account for absence of lactation in females without pups.

The PCB concentration in females of age class $i+1$ at the end of the **delay period** ($j=d,\ \tau=\Delta\tau_d$) during year $t+1$ is obtained from Eq. (26) as:

$$\begin{matrix}\hat{c}_i^d(t+1)=b_{i,i}^d\hat{c}_i^l(t+1)+\phi_i^d\bar{c}_{D,i}(t+1)\\ i=2,\dots,46\end{matrix}\quad,\quad\begin{cases}b_{i,i}^d=e^{-\bar{k}_{E,i}^d\Delta\tau_d}\\ \phi_i^d=\kappa_{D,i}^d\left(1-b_{i,i}^d\right)\end{cases} \tag{30}$$

The PCB concentration in females of age class $i+1$ at the end of the **gestation period** ($j=g,\ \tau=\Delta\tau_g$) year $t+1$ is obtained from Eq. (26) as:

$$\begin{matrix}\hat{c}_i^g(t+1)=b_{i,i}^g(t+1)\hat{c}_i^d(t+1)+\phi_i^g(t+1)\bar{c}_{D,i}(t+1)\\ i=2,\dots,46\end{matrix}\quad,\quad\begin{cases}b_{i,i}^g(t)=e^{-\bar{k}_{E,i}^g(t)\Delta\tau_g}\\ \phi_i^g(t)=\bar{\kappa}_{D,i}^g[1-b_{i,i}^g(t)]\\ \bar{\kappa}_{D,i}^g=k_{D,i}^g/\bar{k}_{E,i}^g\end{cases} \tag{31}$$

The mean elimination rate (accounting for removal, placental transfer and growth dilution) during the gestation period is:

$$\bar{k}_{E,i}^{g}(t)=k_{R,i}+\bar{k}_{P,i}(t)+k_{G,i}^{g}\quad,\qquad i=2,\dots,46 \tag{32}$$

The mean placental transfer rate (for females of age class $i+1$) during the gestation period (year $t+1$) is:

$$\bar{k}_{P,i}(t)=2F_i^d(t)k_{P,i}\quad,\qquad i=2,\dots,46 \tag{33}$$

Here, $F_i^d(t)$ is the fertility for females of age class i at the end of the delay period year t, reduced from ideal fertility due to reproductive stress (obtained from the toxicodynamic model). The factor $2F_i^d(t)$ is an estimation of the mean number of fetuses that each female (of age class i) produces during year t. The factor is included to account for absence of placental transfer in non-pregnant females. Equations (27), (30) and (31) are used to successively calculate PCB concentrations in females at the end of the lactation period, the delay period and the gestation period for one year at a time.

2.2.2. Bioaccumulation in Fetuses

Fetuses assimilate PCB only through vertical transfer via the placenta. It is assumed that all PCB that is eliminated by a mother through placental transfer is assimilated by her fetus, which has no capacity to eliminate it. The toxicokinetic equation is a mass balance where the rate of change of PCB in the fetus equals the rate at which PCB is eliminated from the mother through placental transfer. The rate of change of the total amount of PCB in a fetus with a mother of age class i during the gestation period ($j = g$) is:

$$\frac{\mathrm{d}}{\mathrm{d}\tau}\left(W_0 c_{0,i}^g\right) = k_{P,i} W_i c_{i,i}^g \quad , \qquad i = 2, \dots, 46 \, , \qquad 0 \le \tau \le \Delta\tau_g \tag{34}$$

Here, $c_{0,i}^g$ and $c_{i,i}^g$ are PCB concentrations in fetus and mother, W_0 and W_i are body weights of fetus and mother, whereas $k_{P,i}$ is the placental transfer rate constant. The toxicokinetic equation (Eq. (34)) can be rewritten as:

$$\frac{\mathrm{d}c_{0,i}^g}{\mathrm{d}\tau} + k_{G,0} c_{0,i}^g = k_{P,i} \omega_{0,i} c_{i,i}^g \quad , \qquad i = 2, \dots, 46 \tag{35}$$

The fetus grows exponentially according to Eq. (1). Hence, the growth dilution rate $k_{G,0}$ equals the exponential growth rate:

$$k_{G,0} = \frac{1}{W_0} \frac{\mathrm{d}W_0}{\mathrm{d}\tau} = g_0 \tag{36}$$

The body weight ratio between female and fetus ($\omega_{0,i}$) is approximated as a mean value over the gestation period:

$$\omega_{0,i} = W_i / W_0 \approx \bar{W}_i^g / \bar{W}_0 \quad , \qquad i = 2, \dots, 46 \tag{37}$$

The PCB concentration in the mother ($c_{i,i}^g$) during the gestation period ($j = g$) is obtained from Eq. (26) and inserted into the toxicokinetic equation (Eq. (35)), which yields the following first order ODE:

$$\frac{\mathrm{d}c_{0,i}^g}{\mathrm{d}\tau} + k_{G,0} c_{0,i}^g = \tilde{k}_{P0,i}\left(C_i^g e^{-\tilde{k}_{E,i}^g \tau} + \kappa_{D,i}^g \bar{c}_{D,i}\right) \quad , \qquad \tilde{k}_{P0,i} = k_{P,i}\omega_{0,i} \;\; , \quad i = 2, \dots, 46 \tag{38}$$

The solution to Eq. (38) is:

$$c_{0,i}^g(\tau) = \kappa_{P0,i}\bar{c}_{D,i} + \tilde{\kappa}_{P0,i} C_i^g e^{-\tilde{k}_{E,i}^g \tau} + C_{0,i}^g e^{-k_{G,0}\tau} \quad , \qquad \begin{cases} \tilde{\kappa}_{P0,i} = \tilde{k}_{P0,i}/(k_{G,0} - \tilde{k}_{E,i}^g) \\ \kappa_{P0,i} = \kappa_{D,i}^g \tilde{k}_{P0,i}/k_{G,0} \\ C_{0,i}^g = (\kappa_{D,i}^g \, \tilde{\kappa}_{P0,i} - \kappa_{P0,i})\bar{c}_{D,i} - \tilde{\kappa}_{P0,i} c_{i,i}^g(0) \end{cases} \tag{39}$$

Equation (39) expresses the PCB concentration in a fetus with a mother of age class i at time τ during the gestation period ($j = g$). The first term accounts for dietary uptake in the mother, transferred to the fetus. The second term accounts for reduction of transferred PCB due to elimination in the mother (exponential decrease, characterized by elimination rate $\tilde{k}_{E,i}^g$). The last term accounts for growth dilution in the fetus (exponential decrease, characterized by growth dilution rate $k_{G,0}$). From Eq. (39), with $\tau = \Delta\tau_g$, the PCB concentration in fetuses with mothers of age class i at the end of the gestation period year $t + 1$ is obtained as:

$$\hat{c}_{0,i}^g(t+1) = \tilde{b}_{i,i}^0 \hat{c}_i^d(t+1) + \tilde{\phi}_i^0 \bar{c}_{D,i}(t+1) \quad , \qquad i = 2, \dots, 46 \qquad \begin{cases} \tilde{b}_{i,i}^0 = \tilde{\kappa}_{P0,i}\left(e^{-\tilde{k}_{E,i}^g \Delta\tau_g} - e^{-k_{G,0}\Delta\tau_g}\right) \\ \tilde{\phi}_i^0 = \kappa_{P0,i}(1 - e^{-k_{G,0}\Delta\tau_g}) - \kappa_{D,i}^g \tilde{b}_{i,i}^0 \end{cases} \tag{40}$$

Equation (40) is used to calculate PCB concentrations in fetuses at year ends, based on known PCB levels in their mothers at the end of the delay period.

2.2.3. Bioaccumulation in Pups

Bioaccumulation of PCB in pups (age class $i = 1$) is described by a toxicokinetic equation, similar to Eq. (13) for females, but with no placental transfer and a positive contribution from vertical transfer through lactation. The rate of change of the total amount of PCB in a pup with a mother of age class i is:

$$\frac{\mathrm{d}}{\mathrm{d}\tau}\left(W_1 c_{1,i}^j\right) = k_{L1,i}^j W_i c_{i,i}^j + k_{D,1}^j W_1 c_{D,1} - k_{E,1}^j W_1 c_{1,i}^j \quad , \quad i = 2, \dots, 46 \ , \ j = l, d, g \ , \quad 0 \le \tau \le \Delta\tau_j \tag{41}$$

Here, $c_{1,i}^j$ and $c_{i,i}^j$ are PCB concentrations in pups and mothers, $c_{D,1}$ is the PCB concentration in the diet, whereas W_1 and W_i are body weights of pups and mothers. The lactational transfer rate $k_{L1,i}^j$ for pups with mothers of age class i, accounting for PCB uptake through breast milk during lactation, is:

$$k_{L1,i}^j = \begin{cases} \varphi_L k_{L,i} & , \quad j = l \\ 0 & , \quad j = d, g \end{cases} \tag{42}$$

Here, $k_{L,i}$ is the lactational transfer rate constant for females of age class i. The fraction of transferred PCB that is assimilated by the pup is assumed to equal the lactation assimilation efficiency φ_L, the fraction of consumed milk that is assimilated into energy by a pup during lactation. The dietary uptake rate for pups $k_{D,1}^j$ is calculated analogously to females (Eq. (14)), with prey consumption rate $\dot{W}_{D,1}$ specific to pups. The elimination rate, accounting for all pup elimination routes, is:

$$k_{E,1}^j = \begin{cases} 0 & , \quad j = l \\ k_{R,1} & , \quad j = d, g \end{cases} \tag{43}$$

The removal rate constant for pups ($k_{R,1}$) is defined analogously to females (Eq. (18)). The toxicokinetic equation (Eq. (41)) can be rewritten as the following differential equation for the PCB concentration $c_{1,i}(\tau)$ at time τ in a pup with a mother of age class i during time period j:

$$\frac{\mathrm{d}c_{1,i}^j}{\mathrm{d}\tau} + \left(k_{E,1}^j + k_{G,1}^j\right) c_{1,i}^j = k_{L1,i}^j \omega_{1,i} c_{i,i}^j + k_{D,1}^j c_{D,1} \quad , \quad i = 2, \dots, 46 \tag{44}$$

The body weight ratio between mother and pup is approximated as a mean value over the lactation period:

$$\omega_{1,i} = W_i / W_1 \approx \bar{W}_i^l / \bar{W}_1^l \quad , \quad i = 2, \dots, 46 \tag{45}$$

Here, $\bar{W}_i^l$ ($i = 1, \dots, 46$) are the mean body weights during the lactation period. The growth dilution rate $k_{G,1}^j$ is defined analogously to females (Eq. (20)) and is approximated as the mean growth dilution rate during a time period j ($\tau_0^j \le \tau \le \tau_0^j + \Delta\tau_j$) according to Eq. (21). The effective elimination rate $\tilde{k}_{E,1}^j$ for pups during time period j is defined analogously to females (Eq. (24)). With constant mean PCB concentration in the diet throughout each year ($c_{D,1} = \bar{c}_{D,1}$), the toxicokinetic equation (Eq. (44)) can be approximated as the following differential equation:

$$\frac{\mathrm{d}c_{1,i}^j}{\mathrm{d}\tau} + \tilde{k}_{E,1}^j c_{1,i}^j = \tilde{k}_{L1,i}^j c_{i,i}^j + k_{D,1}^j \bar{c}_{D,1} \quad , \quad \tilde{k}_{L1,i}^j = k_{L1,i}^j \omega_{1,i} \quad , \quad i = 2, \dots, 46 \quad , \quad j = l, d, g \tag{46}$$

The PCB concentration in the mother ($c_{i,i}^j$) during the lactation period ($j = l$) is obtained from Eq. (26) and inserted into Eq. (46), which yields the following first order ODE:

$$\frac{\mathrm{d}c_{1,i}^j}{\mathrm{d}\tau} + \tilde{k}_{E,1}^j c_{1,i}^j = \tilde{k}_{L1,i}^j c_{i,i}^j(0) e^{-\tilde{k}_{E,i}^j \tau} + k_{D,1}^j \bar{c}_{D,1} \quad , \quad i = 2, \dots, 46 \tag{47}$$

The solution to Eq. (47) for a time period $0 \le \tau \le \Delta\tau_j$ within a year is:

$$c_{1,i}^{j}(\tau) = \kappa_{D,1}^{j}\bar{c}_{D,1} + \tilde{\kappa}_{L1,i}^{j} c_{i,i}^{j}(0) e^{-\tilde{k}_{E,i}^{j}\tau} + C_{1,i}^{j} e^{-\tilde{k}_{E,1}^{j}\tau} \quad , \quad \begin{cases} \tilde{\kappa}_{L1,i}^{j} = \tilde{k}_{L1,i}^{j}/(\tilde{k}_{E,1}^{j} - \tilde{k}_{E,i}^{j}) \\ \kappa_{D,1}^{j} = k_{D,1}^{j}/\tilde{k}_{E,1}^{j} \\ C_{1,i}^{j} = c_{1,i}(0) - \tilde{\kappa}_{L1,i}^{j} c_i(0) - \kappa_{D,1}^{j}\bar{c}_{D,1} \end{cases} \tag{48}$$

Equation (48) expresses the PCB concentration in a pup with a mother of age class i at time τ during time period j. The first term accounts for dietary uptake in the pup (proportional to dietary PCB concentration, characterized by constant $\kappa_{D,1}^{j}$). The second term accounts for lactational transfer from mother to pup, reduced by elimination in the mother (exponential decrease, characterized by elimination rate $\tilde{k}_{E,i}^{j}$). The last term accounts for elimination in the pup (exponential decrease, characterized by elimination rate $\tilde{k}_{E,1}^{j}$). Equation (48)is used to obtain the PCB concentration in pups at the end of different time periods j, based on initial PCB concentrations $c_{i,i}^{j}(0)$ at the start of the periods. The concentrations obtained at the end of a period is used as initial value for the next.

The initial PCB concentration in pups with mothers of age class $i+1$ (at the start of the lactation period year $t+1$) is assumed to equal the PCB concentration $\hat{c}_{0,i}^{g}(t)$ in fetuses with mothers of age class i at the end of previous year (t). The PCB concentration in pups with mothers of age class $i+1$ at the end of the **lactation period** $(j = l, \tau = \Delta\tau_l)$ year $t+1$ is obtained from Eq. (48) as:

$$\hat{c}_{1,i+1}^{l}(t+1) = p\hat{c}_{0,i}^{g}(t) + \tilde{b}_{i+1,i}^{l}\hat{c}_{i}^{g}(t) \quad , \quad i = 1, \dots, 45 \quad , \quad \begin{cases} p = e^{-k_{G,1}^{l}\Delta\tau_l} \\ \tilde{b}_{i+1,\mathrm{i}}^{l} = \tilde{\kappa}_{L1,i+1}^{l}\left(e^{-\tilde{k}_{E,i+1}^{l}\Delta\tau_l} - e^{-k_{G,1}^{l}\Delta\tau_l}\right) \end{cases} \tag{49}$$

The PCB concentration in pups with mothers of age class i at the end of the **delay period** $(j = d, \tau = \Delta\tau_d)$ year $t+1$ is obtained from Eq. (48) as:

$$\hat{c}_{1,i}^{d}(t+1) = \tilde{b}^{d}\hat{c}_{1,\mathrm{i}}^{l}(t+1) + \tilde{\phi}^{d}\bar{c}_{D,1}(t+1) \quad , \quad i = 2, \dots, 46 \quad , \quad \begin{cases} \tilde{b}^{d} = e^{-\tilde{k}_{E,1}^{d}\Delta\tau_d} \\ \tilde{\phi}^{d} = \kappa_{D,1}^{d}(1 - \tilde{b}^{d}) \end{cases} \tag{50}$$

The PCB concentration in pups with mothers of age class i at the end of the **gestation period** $(j = g, \tau = \Delta\tau_g)$ year $t+1$ is obtained from Eq. (48) as:

$$\hat{c}_{1,i}^{g}(t+1) = \tilde{b}^{g}\hat{c}_{1,i}^{d}(t+1) + \tilde{\phi}^{g}\bar{c}_{D,1}(t+1) \quad , \quad i = 2, \dots, 46 \quad , \quad \begin{cases} \tilde{b}^{g} = e^{-\tilde{k}_{E,1}^{g}\Delta\tau_g} \\ \tilde{\phi}^{g} = \kappa_{D,1}^{g}(1 - \tilde{b}^{g}) \end{cases} \tag{51}$$

Equations (49), (50) and (51) are used to successively calculate PCB concentrations in pups at the end of the lactation period, the delay period and the gestation period for one year at a time.

Females that enter age class 2 are assumed to have an initial PCB concentration that equals the mean PCB concentration in one year old pups at the end of previous year (just before the occurrence of deaths and births). The mean PCB concentration in pups at the end of year $t+1$ is calculated as:

$$\hat{c}_{1}^{g}(t+1) = \sum_{i=1}^{45}\eta_{1,i+1}(t+1)\hat{c}_{1,i+1}^{g}(t+1) \quad , \quad \eta_{1,i+1}(t+1) = P_i(t)F_i(t)N_i(t-1)/N_1(t) \tag{52}$$

Here, $P_i(t)$ and $F_i(t)$ is the survival rate and fertility of females in age class i at the end of year t, whereas $N_i(t-1)$ is the number of females in age class i at the end of year $t-1$ (after the occurrence of deaths). Hence, $P_i(t)F_i(t)N_i(t-1)$ is the number of pups born by mothers of age class i at the end of year t, i.e. the number of pups with mothers of age class $i+1$ during year $t+1$.

3. Toxicodynamic Model

The toxicodynamic model describes how PCB body loads of fetuses, pups and females cause cumulation of damage, that results in hazard and reproductive stress and how these are translated into reduced survival and fertility (Fig. 6). The toxicodynamics are based on the threshold damage model (TDM), introduced by Ashauer et al. (2007). TDM is in turn based on DEBtox theory but adds cumulative effects and capacity to recover.

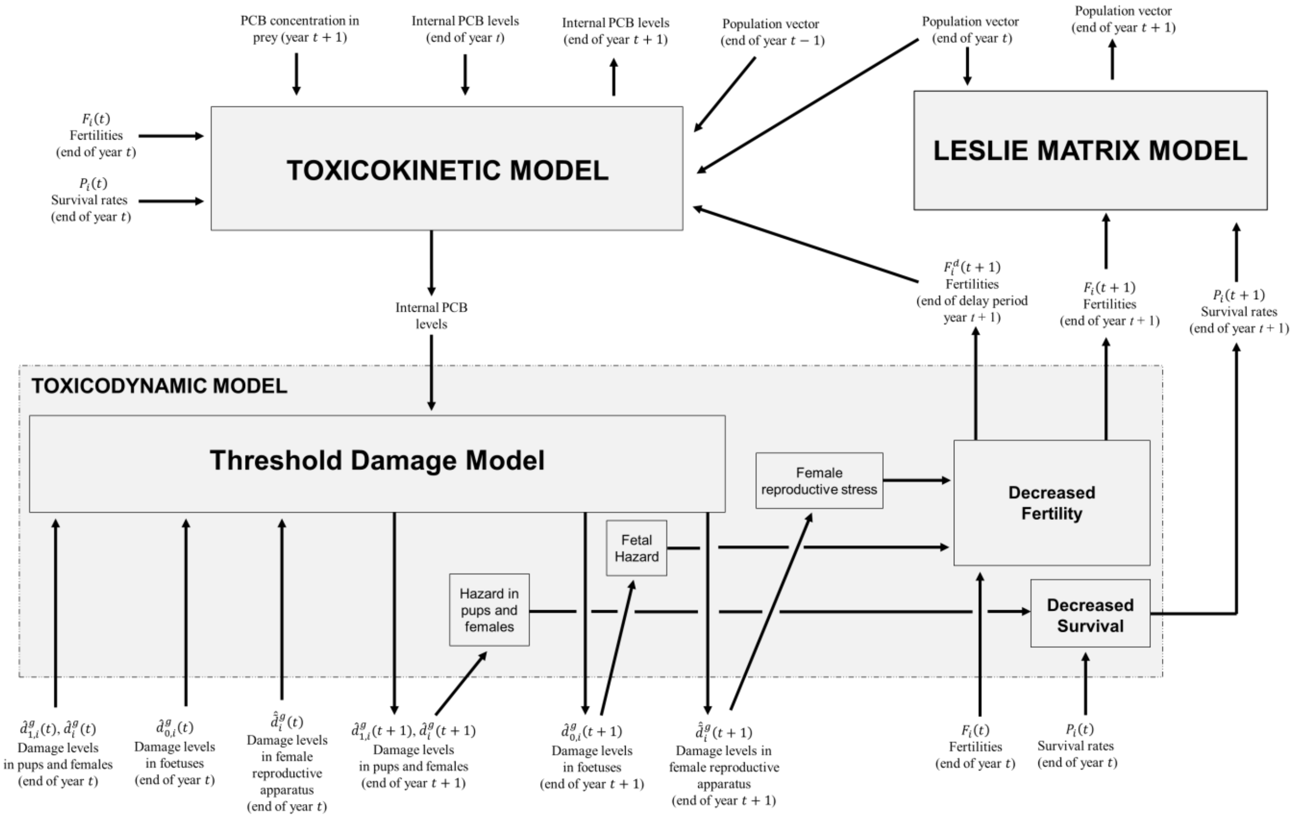

Fig. 6 The toxicodynamic model and its connection to other sub models. Text boxes represent sub modules. Free texts are inputs and outputs, with arrows showing relations to sub modules. The flowchart illustrates computations performed for year $t+1$, based on inputs from the previous year t.

DEBtox assumes several physiological modes of action (pMoA) for toxicants in terms of which DEB parameters the toxicant stress affects (e.g. effects on growth, maturation and reproduction). Two PMoAs act directly on reproduction; *reproductions costs* (costs for *maturation* of females) and *embryonic hazard* (increased costs for *somatic maintenance* of embryos). *Reproduction costs* represent reduced efficiency in the utilization of energy for maturation of reproductive organs and offspring production, whereas *embryonic hazard* causes increased offspring mortality (an embryo dies if its somatic maintenance costs cannot be paid).

The current TD model follows the general principles of DEBtox in assuming that the toxic effects of PCBs on grey seals are considered through the lens of particular pMoAs affecting fundamental physiological processes like somatic maintenance. While our model is not based on DEB theory in the description of energetics and lifehistory traits, we interpret internal PCB concentration effects using a hazard model to account for mortality of adults, pups and fetuses, and damage to reproductive organs. In this context, we assume that decreased survival results from increased somatic maintenance costs (e.g., repair of lesions) due to cumulative damage causing hazard in pups and females. Decreased reproduction results from increased fetal mortality (fetal hazard) and reduced offspring production (reproductive stress). Instead of adopting a costs model, where negative reproductive effects of damaged reproductive organs result from increased costs for offspring production, we

adopted a hazard model and considered reduced offspring production as a result of increased costs for maintenance of the reproductive apparatus due to cumulated damage (representing reproductive organs lesions etc.). In the hazard model, no offspring is produced if the maintenance costs of the reproductive apparatus cannot be paid. The costs model (Billoir, et al., 2007) was also explored to account for damaged reproductive organs, but found to be less consistent with empirical findings and thus not presented herein. Indirect effects on reproduction, such as decreased feeding rate and increased growth costs were neglected. In total, three different kinds of damage were considered: 1) damage to pups and females (causing hazard that decreases survival); 2) damage to reproductive apparatus (causing stress that reduces fertility); and 3) damage to fetuses (causing hazard that kills them and reduce the fertility of their mothers). Toxic effects and corresponding PMoAs are illustrated in Fig. 7.

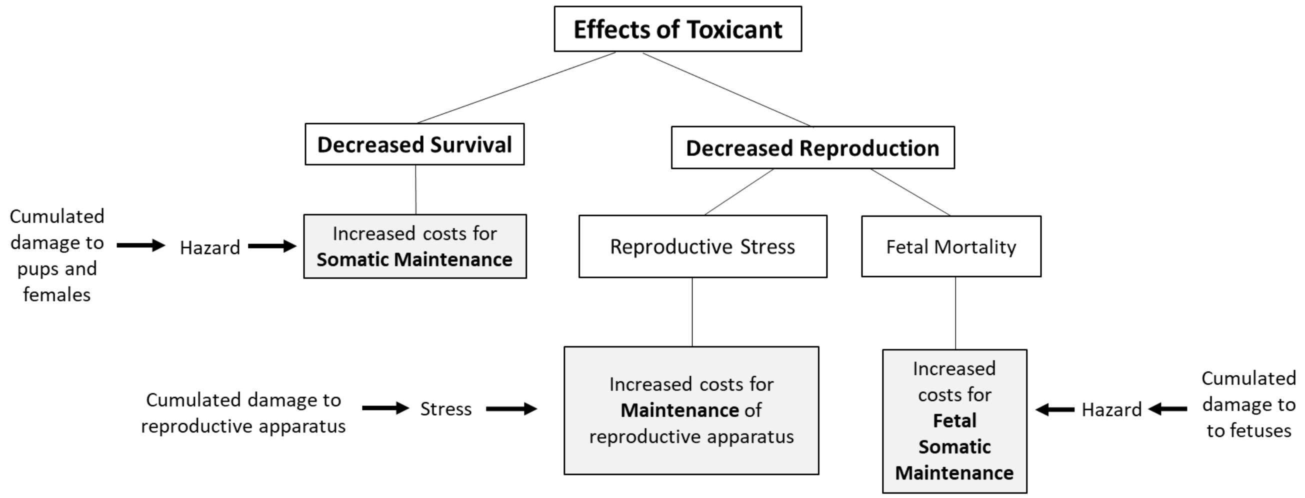

Fig. 7 Effects of toxicant on survival and reproduction considered in the toxicodynamic model and their relation to physical processes (maintenance and maturation).

The *hazard rate*, describing the instantaneous probability to die, is defined as (Jager et al. 2011):

$$\frac{\mathrm{d}h}{\mathrm{d}\tau} = -\frac{1}{P}\frac{\mathrm{d}P}{\mathrm{d}\tau} \tag{53}$$

Here, $h(\tau)$ is the hazard (the probability to die at time τ) and $P(\tau)$ is probability to survive until time τ. Hazard rate multiplied by a small time increment gives increase of *hazard* (probability to die during a time interval). The survival probability (the probability of the organism to survive until time τ) is obtained from Eq. (53) as $P(\tau) = P^0 e^{-h(\tau)}$. The *background survival* (survival probability of animals with no exposure to toxicants) is $P^0 = e^{-m_0}$, where m_0 is the background mortality rate (mortality rate $m(\tau)$ in absence of toxicant exposure). The probability of surviving from one age class $(i-1)$ to the next (i) is given as:

$$P_i = e^{-m(i)} = P^0 e^{-h(i)} \tag{54}$$

The *hazard model* (Billoir et al. 2007) accounts for *fetal hazard* by interpreting effects on reproduction rate as fetal mortality during gestation. Reproductive stress $s(\tau)$, accounting for damaged reproductive apparatus at exposure time τ, is assumed to affect reproduction rate $R(\tau)$ in a similar way (applying the hazard model):

$$R(\tau) = R^0 e^{-s(\tau)} \tag{55}$$

The fertility of age class i is the number of offspring that reach age class 1 per female of age class i:

$$F_i = \int_{i-1}^{i} R(\tau)\,\mathrm{d}\tau \tag{56}$$

3.1 Cumulation of Damage, Hazard and Stress

In order to calculate hazard and stress, a TDM similar to that established by Ashauer et al. (2007) was applied. The cumulation of all three types of damage (damage to pups/females, fetuses and reproductive organs) are described by the same governing equations, which only differ in values of model parameters. According to TDM, the damage rate increases linearly with the internal PCB concentration, whereas the recovery rate is proportional the current damage:

$$\frac{\mathrm{d}d^j_{k,i}}{\mathrm{d}\tau} = \sigma_k c^j_{k,i}(\tau) - r_k d^j_{k,i}(\tau) \quad , \quad \begin{cases} k = 0, 1, i \quad , \quad i = 2, \dots, 46 \\ j = l, d, g \quad , \quad 0 \le \tau \le \Delta\tau_j \end{cases} \tag{57}$$

Here, $d^j_{k,i}(\tau)$ is the damage at time τ (during time period j), $c^j_{k,i}(\tau)$ is the internal PCB concentration (mg/kg) at time τ, σ_k is the *killing* or *stress rate constant* (kg/mg·yr^{-1}) of age class k and r_k is the *recovery rate constant* (yr^{-1}) of age class k. Notice that $k = 0, 1, i$ represents fetuses, pups and females, respectively, whereas $i = 2, \dots, 46$ represents different age classes of females. Hazard (or stress) occurs if the damage exceeds a critical threshold level:

$$h^j_{k,i}(\tau) = \left[d^j_{k,i}(\tau) - d_{T,k}\right]_+ \quad , \quad k = 0, 1, i \, , \quad i = 2, \dots, 46 \, , \quad j = l, d, g \tag{58}$$

Here, $h^j_{k,i}(\tau)$ is the hazard at time τ (during time period j) and $d_{T,k}$ is the *damage threshold level*. The operator $[x]_+ = \max(0, x)$ is applied to ensure positive hazards. Equation (58) is a simplified way of expressing hazard compared to standard TDM (Ashauer et al. 2007) which applies a differential equation. In our model, both damage and hazard are non-dimensional variables. Equations (57) and (58) are expressed in notations used for damage that causes decreased survival of females, pups and fetuses, but analogous equations are applied to describe reproductive damage $\tilde{d}^j_{k,i}$ and reproductive stress $s^j_{k,i}$, only differing in model parameters ($\tilde{\sigma}_k$, $\tilde{r}_k$ and $\tilde{d}_{T,k}$). In DEB theory, embryos and juveniles invest no energy in reproduction. In line with this, fetuses and pups are assumed to be unaffected by stress to the reproductive apparatus ($\tilde{\sigma}_0 = \tilde{\sigma}_1 = 0$), which yields $\tilde{d}^g_{0,i} = \tilde{d}^j_{1,i} = 0$ and $s^g_{0,i} = s^{gj}_{1,i} = 0$. Damage to reproductive organs starts to cumulate when seals enter age class 2. The assumption is probably realistic since PCBs are hormone-like substances and should mainly affect females, with higher oestrogen levels than fetuses and pups.

For a general formulation of the toxicodynamic model, it is useful to summarize equations (26), (39) and (48) as one single equation, expressing the PCB concentrations in fetuses, pups or females at time τ during any time period (enabled by the double index notation in female PCB concentrations):

$$c^j_{k,i}(\tau) = \tilde{C}^j_{k,i} e^{-\tilde{k}^j_{E,k}\tau} + \widetilde{K}^j_{k,i} e^{-\tilde{k}^j_{E,i}\tau} + \overline{K}^j_{k,i} \quad , \quad \begin{cases} k = 0, 1, i \quad , \quad i = 2, \dots, 46 \\ j = l, d, g \quad , \quad 0 \le \tau \le \Delta\tau_j \end{cases} \tag{59}$$

The following definitions were introduced:

$$\tilde{C}^j_{k,i} = \begin{cases} (\kappa^g_{D,i}\, \tilde{\kappa}_{P0,i} - \kappa_{P0,i})\bar{c}_{D,i} - \tilde{\kappa}_{P0,i} c^g_{i,i}(0) & , \ k = 0 \\ c_{1,i}(0) - \tilde{\kappa}^j_{L1,i} c_i(0) - \kappa^j_{D,1}\bar{c}_{D,1} & , \ k = 1 \\ c^j_{i,i}(0) - \kappa^j_{D,i}\bar{c}_{D,i} & , \ k = i \end{cases} \quad , \quad \tilde{k}^g_{E,0} = k_{G,0}$$

$$\widetilde{K}^j_{k,i} = \begin{cases} \tilde{\kappa}_{P0,i}\left(c^g_{i,i}(0) - \kappa^g_{D,i}\bar{c}_{D,i}\right) & , \ k = 0 \\ \tilde{\kappa}^j_{L1,i} c^j_{i,i}(0) & , \ k = 1 \\ 0 & , \ k = i \end{cases} \quad , \quad \overline{K}^j_{k,i} = \begin{cases} \kappa_{P0,i}\bar{c}_{D,i} & , \ k = 0 \\ \kappa^j_{D,1}\bar{c}_{D,1} & , \ k = 1 \\ \kappa^j_{D,i}\bar{c}_{D,i} & , \ k = i \end{cases} \tag{60}$$

Insertion of Eq. (59) into the toxicodynamic equation for damage rate (Eq. (57)) yields the following first order ODE:

$$\frac{\mathrm{d}d^j_{k,i}}{\mathrm{d}\tau} + r_k d^j_k(\tau) = \sigma_k \left(\tilde{C}^j_{k,i} e^{-\tilde{k}^j_{E,k}\tau} + \widetilde{K}^j_{k,i} e^{-\tilde{k}^j_{E,i}\tau} + \overline{K}^j_{k,i}\right) \tag{61}$$

The solution to Eq. (61) is:

$$d_{k,i}^{j}(\tau) = \left(d_{k,i}^{j}(0) - D_{k,i}^{j}\right)e^{-r_k\tau} + E_{k,i}^{j}e^{-\tilde{k}_{E,k}^{j}\tau} + F_{k,i}^{j}e^{-\tilde{k}_{E,i}^{j}\tau} + B_{k,i}^{j} \quad , \quad \begin{cases} k = 0, 1, i \ , \ i = 2, \dots, 46 \\ j = l, d, g \ , \ 0 \le \tau \le \Delta\tau_j \end{cases} \tag{62}$$

The following definitions were introduced:

$$B_{k,i}^{j} = \frac{\sigma_k}{r_k}\bar{K}_{k,i}^{j} \quad , \quad E_{k,i}^{j} = \frac{\sigma_k}{r_k - \hat{k}_{E,k}^{j}}\tilde{C}_{k,i}^{j} \quad , \quad F_{k,i}^{j} = \frac{\sigma_k}{r_k - \hat{k}_{E,i}^{j}}\tilde{K}_{k,i}^{j} \quad , \quad D_{k,i}^{j} = B_{k,i}^{j} + E_{k,i}^{j} + F_{k,i}^{j} \tag{63}$$

Equation (62) can be used to calculate the cumulated damage $d_{k,i}^{j}(\tau)$ in fetuses ($k = 0$), pups ($k = 1$) or females ($k = i$) at a time τ during a time period j within a year when the initial damage $d_{k,i}^{j}(0)$ at the start of the period is known. We used Eq. (62) to successively calculate cumulated damage at the end of each time period ($j = l, d, g$) one year at a time. These included damages in pups and females ($\hat{d}_{1,i}^{j}(t)$ and $\hat{d}_{i}^{j}(t)$), fetal damages $\hat{d}_{0,i}^{g}(t)$ and female reproductive damages $\hat{\hat{d}}_{i}^{j}(t)$.

Obtained damage levels where used as initial values in the next calculation step, similar to how PCB concentrations were successively calculated. The damage level in females of age class $i + 1$ at the start of year $t + 1$ thus equals the damage level in females of age class i at the end of previous year t. The damage level in new-born pups with mothers of age class $i + 1$ equals the damage level in fetuses with mothers of age class i at the end of previous year. The initial damage in fetuses is zero. The initial damage in females of age class 2 at the start of a year was put to the mean damage in pups at the end of previous year, calculated similar to how concentrations were treated in Eq. (52):

$$\hat{d}_{1}^{g}(t) = \sum_{i=1}^{45} \eta_{1,i+1}(t)\hat{d}_{1,i+1}^{g}(t) \tag{64}$$

Here, $\hat{d}_{1,i+1}^{g}(t)$ is the cumulated damage level in pups with mothers of age class $i + 1$ at the end of the gestation period ($j = g$) year t (the end of year t). Notice that calculations for a certain time period require knowledge of damage levels as well as PCB concentrations at the end of previous period.

Based on obtained damage levels, Eq. (58) was used to calculate hazards in pups and females $\hat{h}_{i}^{g}(t)$ at the end of each year t, fetal hazards $\hat{h}_{0,i}^{g}(t)$ at the end of each year t and female reproductive stresses $\hat{s}_{i}^{d}(t)$ at the end of the delay period each year t.

3.2 Fertilities and Survival Rates

Fertilities are affected by PCB exposure in two different ways; reduction due to reproductive stress and reduction due to fetal hazard. It was assumed that the state of females at the end of the delay period (just before gestation starts) determines the fertility reduction due to reproductive stress and that the state of fetuses at the end of the gestation period determines the fertility reduction due to fetal hazard.

The fertility of females in age class i at the end of the delay period year t was obtained from a hazard model (Eqs. (55) and (56)), accounting for decreased pregnancy rates due to reproductive organ lesions:

$$F_{i}^{d}(t) = F_{i}^{0}e^{-\hat{s}_{i}^{d}(t)} \ , \qquad i = 2, \dots, 46 \tag{65}$$

The fertility of females in age class i at the end of year t was obtained by multiplying the fertility at the end of the delay period with a reduction factor accounting for fetal hazard $\hat{h}_{0,i}^{g}$:

$$F_{i}(t) = F_{i}^{d}(t)e^{-\hat{h}_{0,i}^{g}(t)} \ , \qquad i = 2, \dots, 46 \tag{66}$$

Survival rates of pups and females are decreased by hazards $\hat{h}_{i}$. The survival rate for seals of age class i at the end of year t was thus obtained from Eq. (54) as:

$$P_{i}(t) = P_{i}^{0}e^{-\hat{h}_{i}^{g}(t)} \ , \qquad i = 1, \dots, 46 \tag{67}$$

The ideal fertilities and survival rates (F_{i}^{0} and P_{i}^{0}) are vital rates for a population with maximal possible growth rate (in absence of toxicant exposure).

4. Population Model

4.1 Leslie matrix model

Like Harding, et al. (2007), a full age-structured Leslie matrix model for Baltic grey seals was adopted, using annual time steps and 46 age classes. Since an insufficient number of males is unlikely to restrict growth of a seal population (Harding et al. 2007), only the size of the female population was considered. Individuals of age class $i = 1$ (with an age of 0-1 year) are referred to as *pups*, whereas individuals of age class $i = 2, \dots, 46$ are referred to as *females*. Unlike Harding, et al. (2007), the Leslie matrix elements are not constants. Fertilities (F_i) and survival rates (P_i) are linked to damage levels that have cumulated as a result of the history of internal PCB concentrations, which in turn depend on the history of PCB concentrations in the prey. Hence, the elements of the Leslie matrix are changing over time. It was assumed that all deaths occur at the end of a year and immediately after, all pups are born. Since only females that survive to the end of a year give birth to new pups, the first row of the Leslie matrix contains products of survival rates and fertilities, also a difference from Harding, et al. (2007). The Leslie matrix model is formulated as:

$$\mathbf{n}(t+1) = \mathbf{A}(t+1)\mathbf{n}(t) \tag{68}$$

$$\mathbf{A}(t) = \begin{bmatrix} P_1(t)F_1(t) & P_2(t)F_2(t) & \cdots & P_{45}(t)F_{45}(t) & 0 \\ P_1(t) & 0 & \cdots & 0 & 0 \\ 0 & P_2(t) & \cdots & 0 & 0 \\ \vdots & \vdots & \ddots & \vdots & \vdots \\ 0 & 0 & \cdots & P_{45}(t) & 0 \end{bmatrix} \tag{69}$$

The population vector $\mathbf{n}(t)$ includes the number of individuals in different age classes at the end of year t. The Leslie matrix $\mathbf{A}(t)$ includes fertilities and survival rates at the end of year t. Equation (68) is used to calculate the population vector at the end of a year, based on the population vector at the end of previous year. Since the population size is accounted for just after breeding, the model may be characterized as a *post-breeding model*. Ideal fertilities (F_i^0) and survival rates (P_i^0), corresponding to a grey seal population with maximal possible growth rate (in absence of toxicant exposure), were obtained from Harding, et al. (2007).

PCBs concentrations, damage levels and hazard/stress levels in fetuses, pups and females at the end of the different time periods within a year are successively calculated. Fertilities and survival rates are updated based on these computations and a new population vector is calculated, including the number of individuals in different age classes at the end of a year. Outputs from calculations for one year are used as inputs to calculations for the next year. The procedure is repeated for one year at a time and may be continued for as many years as desired.

4.2 Density dependence

Reduced fertilities due to density dependence are calculated in accordance with Caswell (2001) as:

$$F_i^{\text{red}}(t+1) = \frac{F_i(t+1)}{1 + \mathbf{f}_i \bullet \mathbf{n}(t)} \tag{70}$$

Here, $\mathbf{f}_i$ is the *i*th row of $\mathbf{f}$, where f_{ij} is the effect of an individual in age class *j* on the fertility of an individual in age class *i*.

Reduced survival rates due to density dependence are similarly calculated as:

$$P_i^{\text{red}}(t+1) = \frac{P_i(t+1)}{1 + \mathbf{p}_i \bullet \mathbf{n}(t)} \tag{71}$$

Here, $\mathbf{p}_i$ is the *i*th row of $\mathbf{p}$, where p_{ij} is the effect of an individual in age class *j* on the survival of an individual in age class *i*. To include density dependence in the TKTD population model, fertilities and survival rates in the Leslie matrix (Eq. (69)) are substituted by reduced values according to Eqs. (70) and (71). Unless otherwise stated, all performed analyses included density dependence.

5. Model Variables and Parameters

The TKTD population model simulates the temporal dynamics of a number of state variables for Baltic grey seals over their full lifespan (Table 1). We used the model to run simulations for the time period 1966-2015, where data on PCB levels in prey species were available. Segerstedt (2019) compiled data from previous studies of sum-PCB concentrations in the main prey items (cod, sprat and herring) of Baltic grey seals from all regions of the Baltic Sea during 1966-2015. The data are provided as lipid weight concentrations clustered into time intervals of 5 years (Fig. 8). The prey data illustrate that PCB levels peaked in the 1970s for all three fish species. We used the time clustered prey PCB data to linearly interpolate over each five-year period to obtain yearly PCB concentrations in prey, used as input to the TK model.

A five year long pre-simulation period (1961-1965) was included to initiate realistic values of seal PCB and damage levels. Since PCB levels in Baltic fish were low in the early 1960s (Bignert et al. 1998), all PCB and damage levels were put to zero at the start of year 1961. Prey concentrations were assumed to increase linearly from zero (in 1961) to reported levels in 1966. The total population size year 1961 has been estimated to 17 639 seals, including males and females (Harding and Härkönen 1999). The initial population size in the simulation (accounting for females exclusively) was put to $N_0 = 17\,639/2 \approx 8820$.

Table 1. State variables and initial conditions of the TKTD population model. Initial values correspond to year 1961 in simulations.

variable	definition	initial value	unit
$N_i(t)$	Number of individuals in age class i at the end of year t	8820	-
$P_i(t)$	Survival rate of individuals in age class i at the end of year t	-	-
$F_i(t)$	Fertility of females in age class i at the end of year t	-	-
$F_i^d(t)$	Fertility (reduced due to reproductive stress) of females in age class i at the end of the delay period year t	-	-
$\hat{c}_{0,\mathrm{i}}^g(t)$	PCB concentration in fetuses with mothers of age class i at the end of the gestation period year t	0	mg/kg
$\hat{c}_{1,\mathrm{i}}^j(t)$	PCB concentration in pups with mothers of age class i at the end of time period j year t	0	mg/kg
$\hat{c}_i^j(t)$	PCB concentration in females of age class i at the end of time period j year t	0	mg/kg
$\hat{d}_{0,i}^g(t)$	Damage in fetuses with mothers of age class i at the end of year t	0	-
$\hat{d}_{1,i}^j(t)$	Damage in pups with mothers of age class i at the end of time period j year t	0	-
$\hat{d}_i^j(t)$	Damage in females of age class i at the end of time period j year t	0	-
$\hat{\hat{d}}_i^j(t)$	Reproductive damage in females of age class i at the end of time period j year t	0	-
$\hat{h}_{0,i}^g(t)$	Hazard in fetuses with mothers of age class i at the end of year t	-	-
$\hat{h}_{1,i}^j(t)$	Hazard in pups with mothers of age class i at the end of time period j year t	-	-
$\hat{h}_i^j(t)$	Hazard in females of age class i at the end of time period j year t	-	-
$\hat{s}_i^j(t)$	Reproductive stress in females of age class i at the end of time period j year t	-	-

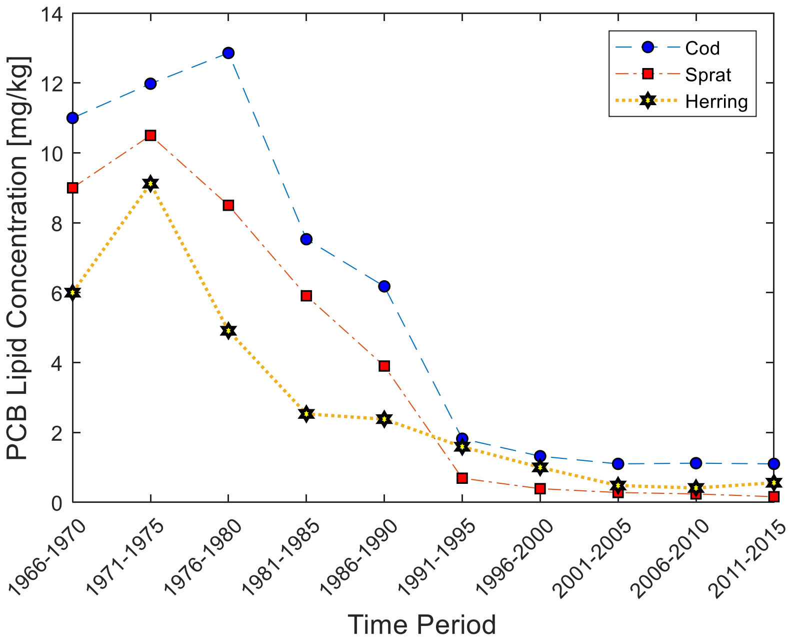

Fig. 8 Temporal sample mean sum-PCB lipid concentrations in three species of fish from the Baltic Sea during 1966-2015, according to data compiled by Segerstedt (2019). The species are the primary prey of Baltic grey seals; cod (*Gadus morhua*), sprat (*Sprattus sprattus*) and herring (*Clupea harengus*).

5.1 Baltic grey seal biology and ecology

The TK model of temporal PCB dynamics incorporates aspects of grey seal life history, feeding habits and growth, as these have important effects on tissue PCB concentrations. The parameters defining the biology and ecology of grey seals in the model are detailed in Tables 2-5.

5.1.1. Grey seal growth and life history parameters

Adult grey seal males have an average weight of 233 kg and mature females have an average weight of 155 kg (Härkönen 2016). Females mature at an age of 3–5 years whereas males mature at around 6 years, although not socially mature until an age of 8 years (Jefferson et al. 2008). The maximum life span for Baltic grey seals is about 35-45 years, though only a minority of the seals become older than 24 years (HELCOM 2018). Baltic grey seals breed and mate in February to March (Jüssi et al. 2008). The fertilized egg is implanted in the uterus after a *delay period* of about 100 days. The succeeding *gestation period* of fetal growth to parturition lasts for about 240 days. Mature females give birth to at most one pup a year (Bergman 2007). The new-born pup has a weight of about 12 kg (Härkönen 2016). The pup is nursed for 15-18 days and weaning is abrupt (Härkönen 2016). During the lactation period, pups daily consume about 4 kg of breast milk (Iverson 1993). The milk contains about 52 % fat. At weaning, the pup has increased its weight to 37-48 kg, whereas the mother's weight loss is massive (Härkönen 2016). Grey seals breed on pack ice formations if they are available and otherwise on land (Jüssi et al. 2008). During two weeks in May and June, hundreds of seals gather at common sites to moult, without feeding (Hansson et al. 2017). Body growth parameters were estimated based on body size data for Baltic grey seals (primarily obtained from Härkönen (2016)) combined with calculations according to section 2.1. Life history and body growth parameters are summarised in Table 2.

Table 2. Life history and body growth parameters

parameter	definition	value	unit	source
$\Delta\tau_l$	Lactation period length	18	d	Härkönen (2016)
$\Delta\tau_d$	Delay period length	100	d	Bergman (2007)
$\Delta\tau_g$	Gestation period length	247	d	Calculation
τ_{SA}	Sub adult age	1	yr	Harding, et al (2007)
τ_{mat}	Sexual maturation age	5	yr	Jefferson, et al.(2008)
τ_{max}	Full-grown age	6	yr	Härkönen (2016)
W_{00}	Initial fetal weight	3.0 x10^{-7}	kg	Calculation (Eq. (2))
W_{birth}	Birth weight	12	kg	Härkönen (2016)
W_{wean}	Weaning weight	43	kg	Härkönen (2016)
W_{mat}	Mature body weight	153	kg	Estimation (Härkönen 2016)
W_{max}	Maximum body weight	160	kg	Härkönen (2016)
W_∞	Asymptotic adult body weight	180	kg	Calculation (Eq. (5))
$W_{lac,6}$	Primiparous post-lactation body weight	105	kg	Estimation (Härkönen 2016)
$W_{lac,7}$	Multiparous post-lactation body weight	110	kg	Estimation (Härkönen 2016)
g_0	Exponential growth rate constant for fetuses and pups	26	yr^{-1}	Calculation (Eq. (4))
g_L	Lactation body weight decline rate constant	7.6	yr^{-1}	Calculation (Eq. (8))
k_6	Primiparous body weight regain rate constant	201	kg/yr	Calculation (Eq. (10))
k_7	Multiparous body weight regain rate constant	183	kg/yr	Calculation (Eq. (10))
γ	von Bertalanffy growth rate constant	0.51	yr^{-1}	Calculation (Eq. (5))

5.1.2. Grey seal diet variables and parameters

The prey consumption factor α_D, the normalisation constant of the allometric relation that describes prey consumption rate $\dot{W}_D$ in terms of body weight W (Eq. (15)), was estimated from empirical data. Hansson, et al. (2017), estimated the daily mean prey consumption of Baltic grey seals to 4.5-5.0 kg, which corresponds to an annual per capita consumption of 1750 kg. It was here assumed that the largest females (with a weight of $W_{\max} = 160$ kg) have a yearly fish consumption of $\overline{W}_{\max}^{\text{fish}} = 1800$ kg. The prey consumption rate $\dot{W}_{D,max}$ for the largest females (with body weight W_{max}) was estimated as the empirically obtained maximum yearly fish consumption of large grey seal females ($\overline{W}_{\max}^{\text{fish}}$) divided by the length of the total feeding period during a year ($\Delta\tau_d + \Delta\tau_g$). The prey consumption factor was thus calculated as:

$$\alpha_D = \frac{\dot{W}_D}{W^{2/3}} \approx \frac{\overline{W}_{\max}^{\text{fish}} / \left(\Delta\tau_d + \Delta\tau_g\right)}{W_{max}^{2/3}} \approx 66\ \text{kg}^{1/3} \cdot \text{yr}^{-1} \qquad (72)$$

According to Hansson, et al. (2017), 80 % of the Baltic fish biomass is constituted by sprat (*Sprattus sprattus*), herring (*Clupea harengus*) and cod (*Gadus morhua*). Our model assumes that Baltic grey seals feed exclusively on these three species. Prey preference indices ϕ_{ij}, describing the preference of prey j by age class i, were calculated from published mean fractions of total prey biomass (Φ_{ij}) found in analyses of gut content in Baltic grey seals (Table 3):

$$\phi_{ij} = \Phi_{ij} / \sum \Phi_{ij} \qquad i = 1, \dots, 46 \quad , \quad j = 1, 2, 3 \qquad (73)$$

The mean body lipid indices $\bar{\rho}_{P,j}$ for different prey species j (Table 3) were obtained based on their fat content according to Fiskbasen (1996).

Table 3. Dietary parameters for seals of different age classes. Estimated fraction of total prey biomass for different species consumed by Baltic grey seals and corresponding prey preference indices. Estimations of total consumed prey biomass are based on gut analyses of Baltic grey seal males and females (Lundström et al. 2010) and females (Tverin et al. 2019). Φ_{ij}: Fraction of total prey biomass. ϕ_{ij}: Prey preference index. $\bar{\rho}_{P,j}$: Mean body lipid for prey species (Fiskbasen 1996).

**prey**	**prey index** (j)	**pups** (i = 1)		**subadults** (i = 2-5)		**adults** ($i \geq 6$)		$\bar{\rho}_{P,j}$
		Φ_{ij}	ϕ_{ij}	Φ_{ij}	ϕ_{ij}	Φ_{ij}	ϕ_{ij}	
herring	1	59 %	0.72	54 %	0.78	42 %	0.98	7.5 %
sprat	2	20 %	0.24	11 %	0.16	0 %	0	15 %
cod	3	3 %	0.04	4 %	0.06	1 %	0.02	1.0 %

The mean sum-PCB concentration in the diet of a seal in age class i was calculated as:

$$\bar{c}_{D,i}(t) = \sum_{j} \phi_{ij} \bar{c}_{P,j}(t) \tag{74}$$

The mean PCB concentration $\bar{c}_{P,j}(t)$ in prey j during year t was obtained from the interpolated data of sum-PCB concentrations in the three prey species, collected by Segerstedt (2019) (Fig. 8).

5.1.3. Conversion of PCB concentrations

Empirical data of PCB concentrations in fish and seals are usually presented on a lipid weight basis, whereas the TKTD population model expresses PCBs in total body concentrations. To make comparisons, it is necessary to convert total body concentrations to lipid concentrations. The body fat index for seals of age class i during time period j is expressed as:

$$\bar{\rho}_i^j = \bar{W}_i^{\mathrm{lip},j} / \bar{W}_i^j \tag{75}$$

Here, $\bar{W}_i^j$ is the mean body weight (kg) of age class i during time period j and $\bar{W}_i^{\mathrm{lip},j}$ is the corresponding body lipid content (kg). As a simplification it was assumed that all PCB in the body of a seal is bounded in fat tissues. The PCB lipid concentration $c_{i,i}^{lip,j}$ can then be expressed in terms of the total body PCB concentration $c_{i,i}^j$ as:

$$c_{i,i}^{lip,j} = c_{i,i}^j / \bar{\rho}_i^j \tag{76}$$

Eq. (76) was used to convert total body PCB concentrations to PCB lipid concentrations. For grey seals, most samples are collected in the autumn, at the middle of the gestation period when seals of all age classes are quite fat (Kauhala et al. 2017). As a simplification, it was assumed that mean body fat indices during sampling are the same for all age classes and remain constant throughout the gestation period; $\bar{\rho}_i^j = \hat{\rho}$. Hence, the PCB lipid concentrations at the end of year t were calculated as:

$$\hat{c}_i^{lip,g}(t) = \hat{c}_i^g(t) / \hat{\rho} \tag{77}$$

Analogously, the total body PCB concentration $\bar{c}_{P,j}(t)$ of prey j during year t was calculated from the PCB lipid concentration $\bar{c}_{P,j}^{\mathrm{lip}}(t)$ as:

$$\bar{c}_{P,j}(t) = \bar{\rho}_{P,j} \bar{c}_{P,j}^{\mathrm{lip}}(t) \tag{78}$$

Here, $\bar{\rho}_{P,j}$ is the mean body lipid index for prey j (Table 3). It was assumed that the mean body lipid index for Baltic grey seals during the gestation period is $\hat{\rho} = 30$ % for all age classes (Iverson 2002).

5.1.4. Grey seal population parameters

Ideal vital rates (F_i^0 and P_i^0) for a population with maximal possible reproduction were used as background values, representing fertilities and survival rates in absence of PCB exposure. These were obtained from Harding et al (2007), who reviewed life history data for grey seals from different parts of the world, derived vital rates for Baltic grey seals and established a Leslie matrix model, generating the observed population growth rate of $\lambda = 1.075$. They also derived optimal vital rates and calculated a maximum possible population growth rate of $\lambda = 1.10$. Values of vital rates are presented in Table 4. Notice that females younger than six years ($i = 1 - 5$) do not reproduce and that the youngest mature females ($i = 6$) have a fertility half that of adult females ($i = 7, \dots, 45$). In the ideal population, adult females obtain almost one pup a year, half of them female pups.

Table 4. Age-specific fertilities and survival rates for Baltic grey seals. Realistic values (F_i, P_i) are values adopted in a population assessment by Harding, et al. (2007). Ideal values (F_i^0, P_i^0) correspond to a population with maximal possible growth rate (Harding et al. 2007). The formers are used as background values in the TKTD population model.

age class (i)	fertility (F_i)	survival (P_i)	fertility (F_i^0)	survival (P_i^0)
1	0	0.700	0	0.700
2-4	0	0.932	0	0.932
5	0	0.950	0	0.950
6	$0.1875 \cdot P_6$	0.950	0.24	0.950
7-45	$0.375 \cdot P_i$	0.950	0.48	0.950
46	0	0	0	0

Since seals are long-lived animals with high adult survival and low fecundity, populations typically consist of a large proportion of adults and the growth rate is low. This makes seal populations relatively resistant to short-term changes in the environment and the population dynamics are primarily governed by competition, predation, disease and availability of prey (Svensson et al. 2010). For long-lived K-strategic species, such as seals, density dependence is an important factor for long-term population dynamics (Kauhala et al. 2014). Seal populations show an age-specific response to increased population density with a rapid decrease in juvenile survival, decreased fecundity and a relatively constant adult survival, changing the age composition towards higher proportions of adults (Svensson et al. 2010). When a population approaches carrying capacity, the mortality is increased foremost among pups and sub-adults of 1-3 years. Hence, the most variable vital parameter in a seal population is pup survival. Pregnancy rates are lowest in the youngest females (4-5 years old), has a maximum during 6-24 years and then start to decline. However, females of 40 years may reproduce (Kauhala et al. 2014). The effects of population density on survival and fertility of grey seals is modelled using age-specific survival and fertility density effect factors (Table 5). The carrying capacity was put to 100 000, since the Baltic grey seal population likely approached that size in the early 1900s (Harding and Härkönen 1999). It was assumed that adult survival and fertility are only dependent on the number of adults, not on the number of pups ($p_{i,1} = f_{i,1} = 0\ ,\ i = 2, \dots, 46$). Here, $p_{i,j}$ and $f_{i,j}$ are effects of an individual in age class j on survival and fertility of an individual in age class i. Since pups are more sensitive to harsh conditions than older animals, it was assumed that pup survival is affected by population density more than other transition rates ($p_{1,j} > p_{i,j}\ ,\ p_{1,j} > f_{i,j}\ ,\ i, j = 2, \dots, 46$). It was also assumed that $p_{1,j} = p_{1,1}$ ($j = 2, \dots, 46$), $p_{1,1} = 2p_{2,2}$, $p_{i,j} = f_{i,j} = p_{2,2}$ ($i, j = 2, \dots, 46$). The adult density factor $p_{2,2}$ was adjusted such that the total population (females and males) approaches carrying capacity after long time under ideal conditions (no adverse effects from contaminants), yielding $p_{2,2} = 2 \cdot 10^{-6}$.

Table 5. Density-dependence parameters. p_{ij}: Survival density effect (effect of an individual in age class j on the survival of an individual in age class i). f_{ij}: Fertility density effect (effect of an individual in age class j on the fertility of an individual in age class i).

parameter	definition	value
$p_{1,1-46}$	Survival density effect of all seals on pups	4 x10^{-6}
$p_{2-46,1}$	Survival density effect of pups on subadults and adults	0
$p_{2-46,2-46}$	Survival density effect of subadults and adults on subadults and adults	2 x10^{-6}
$f_{1-46,1}$	Fertility density effect of pups on all seals	0
$f_{1-5,1-46}$	Fertility density effect of all seals on pups and subadults	0
$f_{6-46,2-46}$	Fertility density effect of subadults and adults on adults	2 x10^{-6}

5.2 Toxicokinetics

Model parameters used in the TK model to describe temporal dynamics of PCB concentrations in seals throughout their lifespan are presented in Table 6, including rate constants that regulate PCB accumulation and depuration in grey seals, covering vertical transfer processes (placental and lactational transfer), dietary uptake, metabolic transformation, fecal egestion, and growth dilution. The derived parameter values are described below.

5.2.1. Assimilation efficiencies

In a bioaccumulation model for arctic ringed seals, Hickie, et al. (2005) adopted an assimilation efficiency of 90 %, both for uptake through diet and lactation. We adopted the same values for Baltic grey seals:

$$\varphi_D = \varphi_L = 0.90$$

Here, φ_D are the diet assimilation efficiency and φ_L is the lactation assimilation efficiency.

5.2.2 Vertical transfer rate constants

To estimate lactational transfer rate constants ($k_{L,i}$) and placental transfer rate constants ($k_{P,i}$), published data on milk transfer in lactating Canadian grey seals (Lang et al. 2011) was combined with published data on PCB transfer through breast milk in Scottish grey seals (Berghe et al. 2012). Lang, et al. (2011) presented body weight, body composition (fractions of water, protein and fat), daily milk output and milk composition for females and their pups at early and late lactation. A distinction was made between *primiparous females* (first-time breeders) and *multiparous females* (experienced breeders). Primiparous females had lower daily milk production, shorter lactation periods and smaller pups at weaning, compared to multiparous females. Berghe, et al. (2012) presented data on PCB concentrations on a lipid-weight basis for female-pup pairs at early and late lactation, including PCB levels in maternal outer and inner blubber, maternal blood, breast milk and pup blood. PCB concentrations increased significantly in all analysed tissues and milk between early and late lactation. In order to estimate lactational transfer rate constants from mentioned publications, it was assumed that the total PCB body burden in females decreases exponentially during lactation:

$$W_i^{PCB}(\tau) \approx W_i^{PCB}(0)e^{-k_{L,i}\tau}, \qquad i = 6, \dots, 46 \tag{79}$$

Here, $W_i^{PCB}(\tau)$ is the total PCB body burden of females at time τ during the lactation period ($0 \leq \tau \leq \Delta\tau_l$). Lactational transfer rate constants were estimated as:

$$k_{L,i} \approx \frac{\ln\left[W_i^{PCB}(\tau_{early})\right] - \ln\left[W_i^{PCB}(\tau_{late})\right]}{\tau_{late} - \tau_{early}}, \qquad i = 6, \dots, 46 \tag{80}$$

In order to estimate total PCB body burdens $W_i^{PCB}(\tau)$ at early lactation ($\tau = \tau_{early}$) and late lactation ($\tau = \tau_{late}$) from

published data, the following assumptions were made: 1) all fat in a female body is either *outer blubber* or *inner blubber* and both layers are reduced during lactation; 2) the total amount of PCB in the outer blubber layer is constant throughout lactation and PCB concentration change is exclusively an effect of reduced blubber mass; 3) the inner blubber layer reduces linearly with time throughout lactation; 4) all inner blubber is consumed at the end of lactation; 5) the total PCB body burden of a female is reduced by the same amount as is transferred to her pup through breast milk during lactation; 6) milk PCB level increases linearly with time throughout lactation; 7) lost female body weight that is not blubber contains the PCB required to obtain the total PCB body burden according to assumption 5. The blood volume of grey seals is about 213 ml/kg and mammals have a blood density of 1060 kg/m^3 (Castellini and Mellish 2016). We assumed that about 10 % of a seal body is constituted by bone and estimated a blood weight fraction of 22.6 %. Assumptions 1-4 were used in calculations of outer/inner blubber ratios and assumptions 5-7 in calculations of total PCB body burdens.

The obtained lactational transfer rate constant for primiparous females $k_{L,6} = 4.36/\text{yr}$ corresponds to a 19 % reduction of the total PCB body burden during lactation (18 days), whereas the obtained lactational transfer rate constant for multiparous females $k_{L,i} = 5.37/\text{yr}$ corresponds to a 23 % reduction. As a comparison, Addison and Brodie (1977) proposed that female grey seals lose about 15 % of their total PCB body burden during the nursing period.

In order to estimate placental transfer rate constants from mentioned publications, it was assumed that that the total PCB body burden in females during gestation decreases exponentially under constant body weight:

$$c_i(\Delta\tau) \approx c_i(0)e^{-k_{P,i}\Delta\tau}, \qquad i = 6, \dots, 46 \tag{81}$$

Here, c_i is total body PCB concentration in females at time $\Delta\tau$ during the gestation period ($0 \leq \Delta\tau \leq \Delta\tau_g$). The placental transfer rate constant was estimated as:

$$k_{P,i} \approx \left(\ln[c_i(0)] - \ln\left[c_i(\Delta\tau_g)\right]\right)/\Delta\tau_g \quad , \qquad i = 6, \dots, 46 \tag{82}$$

In order to estimate PCB concentrations c_i before gestation ($\Delta\tau = 0$) and after gestation ($\Delta\tau = \Delta\tau_g$) from published data, the following assumptions were made: 1) blood PCB levels increase linearly in females and pups during lactation; 2) pup body and blubber weights increase exponentially from birth to late lactation; 3) the female outer blubber layer has the same weight at parturition and early lactation; 4) the blood/blubber PCB concentration ratio is the same for females and pups at parturition; 5) all PCB in females and pups at parturition is located in blubber; 6) the reduction of PCB body burden in females during gestation equals the total PCB body burden in pups at birth (dietary uptake and removal during gestation are neglected).

The obtained placental transfer rate constant $k_{P,i} = 0.07/\text{yr}$ ($i = 6, \dots, 46$) corresponds to a 4 % loss of the total PCB body burden in females during gestation (247 days), which can be compared to the 19-23 % loss during lactation (18 days). This agrees with the general view that vertical PCB transfer in marine mammals is dominated by lactation, with only a minor contribution from placental transfer (Subramanian et al. 1988).

5.2.3. Removal rate constants

The removal rate constants $k_{R,i} = k_{M,i} + k_{F,i}$ account for metabolic transformation and fecal egestion. According to Klanjscek et al. (2007), metabolic transformation of PCB in marine mammals is negligible, but we assume that a small fraction may be removed through metabolic transformation. In their bioaccumulation model for artic ringed seals, Hickie, et al. (2005) estimated elimination rate constants for 20 different PCB congeners. Linear regression was used to fit model outputs with observed contaminant concentrations in the seals. The estimated elimination rate constants ranged from 0.03/yr (PCB 153) to 2.5/yr (PCB 18), with 0.17/yr for sum-PCB. We considered these as removal rate constants, accounting for both metabolic transformation and fecal egestion. It was assumed that removal rate is proportional to metabolic rate (rate of energy spent per unit time) and that the former scales allometrically within and between seal species according to Kleiber's law. Metabolic rate R thus increases with body mass W according to a power law (Kleiber 1962):

$$R = aW^{3/4} \tag{83}$$

The normalisation constant a is assumed to be similar for similar species of mammals. Larger animals thus metabolize PCB at a higher total rate than smaller animals, but smaller animals metabolize PCB faster per unit of body weight. The specific

PCB removal rate is assumed to be proportional to the specific metabolic rate; $\dot{W}_{PCB}/\ W_{PCB} \propto R/W \propto W^{-1/4}$. It then follows that the removal rate constant (k_R) scales with body weight (W) as $k_R \propto W^{-1/4}$.

Adult ringed seals have a body weight of 50-70 kg (Jefferson et al. 2008). By assuming a mean body weight of $W_{RS} = 60$ kg, removal rate constants for ringed seals ($k_{R,RS}$) can be transformed to removal rate constants for grey seals ($k_{R,i}$) of different age classes i:

$$k_{R,i} = \left(\bar{W}_i^j / W_{RS}\right)^{-1/4} \cdot k_{R,RS} \quad , \quad i = 0, \dots, 46 \tag{84}$$

Here, $\bar{W}_i^j$ is the mean the body weight for age class i during time period j (where i = 0 represents fetuses). The removal rate constant for sum-PCB in arctic ringed seals $k_{R,RS} = 0.17/\text{yr}$ (Hickie et al. 2005) was adopted to calculate values of removal rate constants for Baltic grey seals of different age classes, ranging from 0.1/yr (pregnant females) to 0.5/yr (fetuses).

Table 6. TK model parameters for Baltic grey seals.

parameter	definition	value	unit
α_D	prey consumption factor	66	$\text{kg}^{1/3} \cdot \text{yr}^{-1}$
$\hat{\rho}$	body lipid index	30	%
φ_D	diet assimilation efficiency	0.90	-
φ_L	lactation assimilation efficiency	0.90	-
$k_{L,i}$	PCB lactational transfer rate constants	i = 6: 4.36<br>i > 6: 5.37	yr^{-1}
$k_{P,i}$	PCB placental transfer rate constant	0.07	yr^{-1}
$k_{R,i}$	PCB removal rate constant	0.1-0.5 (Eq. (84))	yr^{-1}

5.3 Toxicodynamics

Model parameters used in the TD model to describe temporal dynamics of PCB effects in seals throughout their lifespan are presented in Table 7. The TD parameters define the rate constants, threshold levels, and tolerance levels that regulate the impact of PCB concentrations on hazard and stress in grey seals of different age classes. The derived parameter values are described below.

The toxicodynamic model includes three sets of parameters; *reproductive stress* parameters, *fetal hazard* parameters and hazard parameters related to *survival* of pups and adults. Each parameter set includes three type of constants; *stress/killing rate constants*, *damage threshold levels* and *recovery rate constants*. With respect to the many empirical observations of reproductive organ lesions in Baltic grey seals during the 1970s and the 1980s, stress to the reproductive apparatus is probably the most crucial path in which PCB affects vital rates. The corresponding reproductive stress parameters were estimated by a calibration procedure where pregnancy rates predicted by the TKTD population model were compared to reported pregnancy rates (Roos et al. 2012). For the other (less crucial) toxicodynamic parameters, values were chosen based on the reproductive stress parameters and on DEBtox parameters from minks (Desforges et al. 2017).

5.3.1 Calibration of Reproductive Stress Parameters

There are a number of publications, reporting historical data on pregnancy rates and prevalence of reproductive organ lesions associated with sterility (uterine occlusions, uterine stenosis and uterine leiomyoma) in Baltic grey seal females (Bergman 1999, Bäcklin et al. 2003, Bergman 2007, Bredhult et al. 2008, Roos et al. 2012, Bäcklin and Moraeus 2013). Roos, et al. (2012) analysed the reproductive outcome of Baltic grey seals in relation to organochlorine concentrations during the time period 1966-2015. Frequency of uterine obstructions (stenosis or occlusions) and uterine tumours were investigated in 277 adult females, whereas pregnancy rates were investigated in 144 adult females (five years or older), collected between 1973 and 2010. Blubber from 292 juvenile grey seals (0-3 years), collected between 1968 and 2010, where used to measure sum-PCB levels on a lipid weight basis. To illustrate general patterns, Roos, et al. (2012) fitted

curves of different forms to temporal trends for reproduction indices and sum-PCB blubber concentrations. They used non-linear regression to fit the following logistic function to the temporal trend of mean pregnancy rates in Baltic grey seal females (age class $i = 6, \dots\ 46$) at year t:

$$\mathrm{PR}(t) = \frac{\exp[-3.83 + 0.24(t - 1975)]}{1 + \exp[-3.83 + 0.24(t - 1975)]} \tag{85}$$

We compared this curve to pregnancy rates predicted by the TKTD population model, using temporal data on PCB concentrations in prey (Segerstedt 2019) as model input. It was assumed that reduced pregnancy is an effect of reproductive stress, caused by damage of the reproductive apparatus. In order to obtain the mean pregnancy rate (PR) from the TKTD population model, the mean fertility for age class 6-46 at the end of the delay period were multiplied by 2 (to include offspring of both sexes):

$$PR(t) = 2\bar{F}^{d}_{6-46}(t) \tag{86}$$

The fertility rate at the end of the year was not used in the calculation, since this fertility also account for reproductive failure due to fetal hazard in pregnant females. The mean fertility of females in age class 6-46 at the end of the delay period year $t + 1$ is obtained as:

$$\bar{F}^{d}_{6-46}(t+1) = \frac{\sum_{i=6}^{46} N_i(t) F_i^d(t+1)}{\sum_{i=6}^{46} N_i(t)} \tag{87}$$

Here, $N_i(t)$ is the number of females in age class i at the end of year t. Pregnancy rates, derived from the TKTD population model, were compared to observed pregnancy rates by visual inspection of graphs (Fig. 9). Reproductive stress parameters $(\tilde{\sigma}_i, \tilde{r}_i, \tilde{d}_{T,i})$ were adjusted to fit model and empirical curve (Table 7).

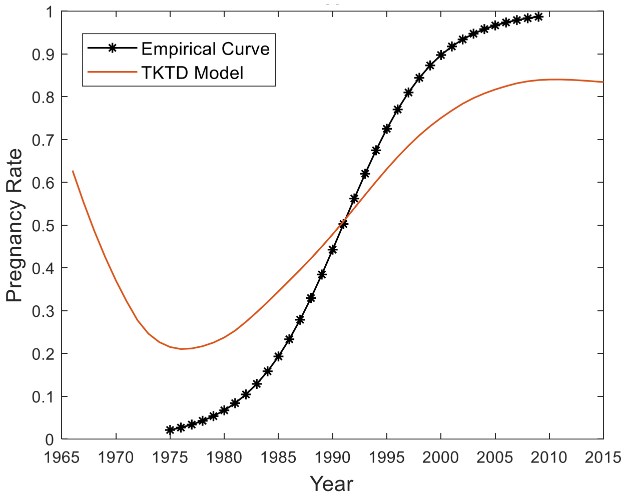

Fig. 9 Pregnancy rates for the Baltic grey seal population between 1966 and 2015 according to empirical curve from Roos, et al. (2012) and mean pregnancy rates for females of ages 5-46 years according to the TKTD population model. Reproductive stress parameters were adjusted to fit model and empirical curve.

5.3.2. Estimation of Toxicodynamic Model Parameters Related to Survival

The stress rate constant with respect to fetal hazard σ_0 was translated from DEBtox parameters for embryonal survival of minks (Desforges et al. 2017), see below (section 5.3.3). The corresponding damage threshold level $d_{T,0}$ was chosen arbitrarily as ten times smaller than the damage threshold level for reproductive stress in females since younger age classes are known to be more sensitive. The recovery rate constant for fetal hazard r_0 was then scaled from the reproductive stress constants; $r_0 = (\sigma_0/\tilde{\sigma}_{2-46})\,\tilde{r}_{2-46}$.

The killing rate constant with respect to pup survival σ_1 was translated from DEBtox parameters for kit survival of minks (Desforges et al. 2017), see below (section 5.3.3). The same killing rate constant was chosen for adults; $\sigma_{2-46} = \sigma_1$. The damage threshold level $d_{T,2-46}$ was adjusted to obtain good agreement between model and historical data for population size. The corresponding damage threshold level for pups was set to half of this value; $d_{T,1} = 0.5 d_{T,2-46}$. The recovery rate constants with respect to pup and female survival were scaled from reproductive stress constants; $r_i = (\sigma_i/\tilde{\sigma}_{2-46})\tilde{r}_{2-46}$.

Table 7. Toxicodynamic parameters. Mink TDM parameters were converted from DEBtox parameters (Desforges et al. 2017). Effects on *reproduction* include *fetal survival* and *reproductive stress*. Effects on *survival* refer to *kit/pup survival*.

parameter	definition	value	unit
σ_i	Killing rate constant (pups and adults)	0.0073	$(\mathrm{kg/mg}) \cdot \mathrm{yr}^{-1}$
r_i	Recovery rate constant (survival)	0.12	yr^{-1}
$d_{T,i}$	Damage threshold level (survival)	$i = 1$: 0.10<br>$i > 1$: 0.20	-
$\tilde{\sigma}_i$	Reproductive stress rate constant	$i = 1$: 0<br>$i > 1$: 1	$(\mathrm{kg/mg}) \cdot \mathrm{yr}^{-1}$
$\tilde{r}_i$	Recovery rate constant (reproductive stress)	16	yr^{-1}
$\tilde{d}_{T,i}$	Damage threshold level (reproductive stress)	0.010	-
σ_0	Killing rate constant (fetus)	0.023	$(\mathrm{kg/mg}) \cdot \mathrm{yr}^{-1}$
r_0	Recovery rate constant (fetus)	0.0010	yr^{-1}
$d_{T,0}$	Damage threshold level (fetus)	0.37	-
NEC_S	DEBtox no effect concentration for stress on fetal survival and reproductive stress in mink	0.017-0.65	mg/kg
c_T	DEBtox stress tolerance for fetal survival and reproductive stress in mink	0.69-2.25	mg/kg
NEC_H	DEBtox no effect concentrations for hazard related to kit survival in mink	2.94-3.71	mg/kg
c_{Th}	DEBtox hazard tolerance for kit survival in mink	9.12×10^{-6} – 1.08×10^{-5}	mg/kg
$\tilde{d}_T$	Damage threshold level (reproductive stress)	mink: 0.0076-0.94<br>seal: 0.018–2.2	-
$\tilde{\sigma}$	Reproductive stress rate constant	mink: 0.011-0.035<br>seal: 0.011-0.035	$(\mathrm{kg/mg}) \cdot \mathrm{yr}^{-1}$
$\tilde{r}_{max}$	Recovery rate constant limit (reproductive stress)	mink: 0.025-3.15<br>seal: 0.0011-1.3	yr^{-1}
d_T	Damage threshold level (survival)	mink: $2.5\cdot10^{-6}$ - $3.7\cdot10^{-6}$<br>seal: 5.8×10^{-6} – 8.7×10^{-6}	-
σ	Killing rate constant (survival)	mink: 0.0064 – 0.0081<br>seal:0.0064-0.0081	$(\mathrm{kg/mg}) \cdot \mathrm{yr}^{-1}$
r_{max}	Recovery rate constant limit (survival)	mink: 6482 – 9686<br>seal: 2741-4097	yr^{-1}

5.3.3. Transformation of DEBtox parameters

The toxicodynamic model is a threshold damage model (TDM), including stress rate constants, recovery rate constants and damage threshold levels. It is not a DEBtox model and does not use stress tolerance and NEC (no effect concentration) as parameters. Here, a rough and simple way to transform DEBtox parameters for one mammal (mink) into TDM parameters for another mammal (grey seal) is suggested. The method can be used as a way to obtain preliminary values before a more accurate parametrization is performed. As a demonstration, DEBtox parameters for mink are transformed into TDM parameters for grey seal. The obtained killing rate constant was used in the TKTD population model Baltic grey seals.

Desforges, et al. (2017) developed a DEBtox model for mink, parameterized from published data on growth and reproduction in captive minks fed with PCB-contaminated fish. Embryos were exposed to PCB through the placenta throughout the gestation period ($\Delta\tau_g^{mink} = 42$ days). Physical modes of action (PMoAs) were identified by comparing dose-response-curves from the model and reported data. Tolerance parameters and no effect concentrations were adjusted to fit model outputs with data. The model accurately predicted PCB accumulation and negative effects on embryo survival, kit survival, growth and development. DEBtox parameters for mink according to Table 7 were estimated (Desforges et al. 2017). Now, the transformation of these parameters is considered.

First, DEBtox parameters are transformed into TDM parameters (for the same species). Assuming no recovery ($r = 0$), the damage rate according to TDM (Eq. (57)) is:

$$\frac{\mathrm{d}d}{\mathrm{d}\tau} = \sigma c(\tau) \tag{88}$$

Assuming no initial damage and constant internal concentration c, the cumulated damage d after exposure time $\Delta\tau$ is:

$$d = \sigma c \Delta\tau \tag{89}$$

The hazard h is obtained from Eq. (58) as:

$$h = [d - d_\mathrm{T}]_+ = [\sigma c \Delta\tau - d_\mathrm{T}]_+ \quad , \quad [x]_+ = \max(0, x) \tag{90}$$

The hazard according to DEBtox theory only depends on the current toxicant concentration and is independent of exposure time:

$$h = [c - \mathrm{NEC}]_+ / c_\mathrm{T} \tag{91}$$

Comparing Eqs. (90) and (91) yields expressions for TDM parameters expressed in DEBtox parameters. The killing rate constant σ is expressed in terms of the tolerance concentration c_T and the exposure time $\Delta\tau$ as:

$$\sigma \approx 1/(c_\mathrm{T}\Delta\tau) \tag{92}$$

The damage threshold level d_T is expressed in terms of the no effect concentration NEC and the tolerance concentration c_T as:

$$d_\mathrm{T} \approx \mathrm{NEC}/c_\mathrm{T} \tag{93}$$

An upper limit for the recovery rate constant r may be estimated by assuming zero damage rate:

$$\frac{\mathrm{d}d}{\mathrm{d}\tau} = \sigma c(\tau) - r_{max} d(\tau) = 0 \tag{94}$$

The maximum recovery rate constant is thus:

$$r_{max} = \sigma c / d \approx \sigma c_T / d_\mathrm{T} = 1/(d_\mathrm{T}\Delta\tau) \tag{95}$$

Since TDM includes cumulative effects, not considered in a DEBtox model, translation of DEBtox parameters into TDM parameters, requires knowledge of experimental exposure time $\Delta\tau$. Mink DEBtox parameters were converted to TDM parameters by applying Eqs. (93) and (95) with exposure time $\Delta\tau_g^{mink} = 42$ days and are presented in Table 7. The TDM parameters for mink were then converted to TDM parameters for grey seal. According to Baas and Kooijman (2015) there is a strong correlation between specific metabolic rate (equated with specific somatic maintenance rate) and sensitivity to a

toxicant (defined as having low NEC). This makes it possible to scale NEC between different animals. From analyses with different combinations of toxicants and animals, Baas & Kooijman (2015) found a strong negative correlation between specific somatic maintenance (p_m) and no effect concentration (NEC):

$$\log(p_m) = m - b \cdot \log(\mathrm{NEC}) \tag{96}$$

Here, m and b are toxicant-specific constants. If p_m and NEC are known for two animals, exposed to a specified toxicant, m and b can be calculated. If p_m is known for a third species, NEC can be estimated also for this one. The specific somatic maintenance for American mink (*Neovison vison*) and grey seal (*Halichoerus grypus*) are found in the Add-My-Pet database (Kooijman et al. 2019):

$$p_m^{mink} = 88.71\ \mathrm{J/(day \cdot cm^3)} \quad , \quad p_m^{seal} = 37.52\ \mathrm{J/(day \cdot cm^3)} \tag{97}$$

With known value on the PCB-specific parameter b, DEBtox data for mink can be converted to DEBtox data for grey seal:

$$\frac{\mathrm{NEC}^{seal}}{\mathrm{NEC}^{mink}} = \left[\frac{p_m^{mink}}{p_m^{seal}}\right]^{1/b} \tag{98}$$

It was here assumed that $b = 1$ and that damage threshold levels can be converted in the same way as no effect concentrations:

$$\frac{d_T^{seal}}{d_T^{mink}} = \left[\frac{p_m^{mink}}{p_m^{seal}}\right]^{1/b} \approx 2.36 \tag{99}$$

It now follows from Eq. (93) that tolerance parameters are the same for different mammals exposed to a specified toxicant ($c_T^{mink} = c_T^{seal}$). By assuming equal exposure times in Eq. (92), also killing rate constants are obtained the same:

$$\sigma^{seal} = \sigma^{mink} \tag{100}$$

TDM parameters for mink were converted to TDM parameters for grey seal by applying Eqs. (95), (99) and (100).

6. Model Simulations

6.1 Steady state

If prey PCB concentrations are constant over time, a stable state will finally be reached where PCB, damage, hazard and stress levels are stationary. If density dependence is neglected, also fertilities and survival rates stabilize at constant values and the population grows (or declines) at constant rate. Under these conditions, biomagnification factors and stable population growth rate can be defined. The mean biomagnification factor for all age classes is:

$$\overline{\mathrm{BMF}} = \lim_{t\to\infty}\left[\bar{c}^{\mathrm{lip}}(t)/\bar{c}_D^{\mathrm{lip}}\right] \quad (101)$$

Here $\bar{c}_i^{\mathrm{lip}}(t)$ is the mean PCB lipid concentration in seals of all age classes and $\bar{c}_D^{\mathrm{lip}}$ is the mean PCB lipid concentration in the diet of all seals. The stable population growth rate λ is calculated as the dominant eigenvalue of the Leslie matrix:

$$\lambda = \lim_{t\to\infty}\left[\max(|\lambda_i(t)|)\right] \quad (102)$$

Here, $\lambda_i(t)$ are the eigenvalues of the Leslie matrix. Different levels of constant PCB lipid concentration in prey (0-10 mg/kg) were used as inputs to the TKTD population model and 100 years were simulated to investigate temporal changes in mean PCB lipid concentrations and population size for Baltic grey seals.

6.2 Sensitivity analysis

A *sensitivity analysis* investigates how uncertainty in input parameters causes uncertainty in population growth and can be used to identify parameters that are critical for population viability (Lacy et al. 2018). Assuming steady state conditions without density dependence (when growth rate is constant and nonzero), sensitivity analyses were performed for three toxicokinetic parameters (lactational transfer rate constants $k_{L,i}$, placental transfer rate constants $k_{P,i}$ and removal rate constants $k_{R,i}$) as well as for all toxicodynamic parameters (killing/stress rate constants, damage threshold levels and recovery rate constants).

A constant PCB lipid concentration in prey, generating positive stable growth rate over time, was used as input and the investigated parameters were varied one at time, whereas other parameters were held constant. The mean prey lipid concentration was set to 2 mg/kg, corresponding to Baltic levels of the early nineties. The relative value of a parameter (p_{rel}) is defined as the perturbed value (p_{pert}) divided by the value adopted in the model (p_{mod}); $p_{rel} = p_{pert}/p_{mod}$. Stable population growth rate was plotted versus perturbed relative value of investigated model parameter.

References

Addison RF, Brodie PF (1977) Organochlorine residues in maternal blubber, milk, and pup blubber from grey seals (Halichoerus grypus) from Sable Island, Nova Scotia. J. Fish Res. Board Can. 34:937–941

Ashauer R, Boxall AB, Brown CD (2007) New Ecotoxicological Model To Simulate Survival of Aquatic Invertebrates after Exposure to Fluctuating and Sequential Pulses of Pesticides. Environmental Science & Technology 41:1480-1486. https://doi.org/10.1021/es061727b

Baas J, Kooijman SALM (2015) Sensitivity of animals to chemical compounds links to metabolic rate. Ecotoxicology 24:657–663. https://doi.org/10.1007/s10646-014-1413-5

Berghe MV, Weijs L, Habran S, Das K, Bugli C, Rees J-F (2012) Selective transfer of persistent organic pollutants and their metabolites in grey seals during lactation. Environment International 46:6–15. https://doi.org/10.1016/j.envint.2012.04.011

Bergman A (1999) Health condition of the Baltic grey seal (Halichoerus grypus) during two decades - Gynaecological health improvement but increased prevalence of colonic ulcers. APMIS 107:270-282. https://doi.org/10.1111/j.1699-0463.1999.tb01554.x

Bergman A (2007) Pathological Changes in Seals in Swedish Waters: The Relation to Environmental Pollution - Tendencies during a 25-year Period. Swedish University of Agricultural Sciences, Uppsala, Sweden, PHD thesis

Bignert A, Olsson M, Persson W, Jensen S, Zakrisson S, Litzfn K, Eriksson U, Häggberg L, Alsberg T (1998) Temporal trends of organochlorines in Northern Europe, 1967-1995. Relation to global fractionation, leakage from sediments and international mesures. Environmental Pollution 99:177-198. https://doi.org/10.1016/S0269-7491(97)00191-7

Billoir E, Péry ARR, Charlesa S (2007) Integrating the lethal and sublethal effects of toxic compounds into the population dynamics of Daphnia magna: A combination of the DEBtox and matrix population models. Ecological Modelling 203:204-214. https://doi.org/10.1016/j.ecolmodel.2006.11.021

Bodiguel X, Maury O, Mellon-Duval C, Roupsard F (2009) A dynamic and mechanistic model of PCB bioaccumulation in the European hake (Merluccius merluccius). Journal of Sea Research 62:124–134. https://doi.org/10.1016/j.seares.2009.02.006

Boyd IL (1984) The relationship between body condition and the timing of implantation in pregnant Grey seals (Halichoerus grypus). J. Zool. 203:113–123

Bredhult C, Bäcklin B-M, Bignert A, Olovsson M (2008) Study of the relation between the incidence of uterine leiomyomas and the concentrations of PCB and DDT in Baltic gray seals. Reproductive Toxicology 25:247–255. https://doi.org/10.1016/j.reprotox.2007.11.008

Bäcklin B-M, Eriksson L, Olovsson M (2003) Histology of Uterine leiomyoma and occurrence in relation to reproductive activity in the Baltic gray seal (Halichoerus grypus). Veterinary Pathology 40:175-180. https://doi.org/10.1354/vp.40-2-175

Bäcklin B-M, Moraeus CK, K. Isomursu, M. (2013) Pregnancy rates of the marine mammals - Particular emphasis on Baltic grey and ringed seals. HELCOM

Castellini MA, Mellish J-A (2016) Marine Mammal Physiology: Requisites for Ocean Living. CRC Press, Boca Raton, US

Caswell H (2001) Matrix population models: Construction, analysis, and interpretation. Sinauer Associates, Inc. Publishers, Sunderland, Massachusetts, USA

Desforges JP, Sonne C, Dietz R (2017) Using energy budgets to combine ecology and toxicology in a mammalian sentinel species. Scientific Reports 7:46267. https://doi.org/10.1038/srep46267

Fiskbasen (1996) Näringsvärden i fisk. Fiskbasen

Hansson S, Bergström U, Bonsdorff E, Härkönen T, Jepsen N, Kautsky L, Lundström K, Lunneryd S-G, Ovegård M, Salmi J, Sendek D, Vetemaa M (2017) Competition for the fish – fish extraction from the Baltic Sea by humans, aquatic mammals, and birds. ICES Journal of Marine Science. https://doi.org/10.1093/icesjms/fsx207

Harding K, Härkönen T (1999) Development in the Baltic grey seal (Halichoerus grypus) and ringed seal (Phoca hispida) populations during the 20th century. Ambio 28:619-627

Harding K, Härkönen T, Helander B, Karlsson O (2007) Status of Baltic grey seals: Population assessment and extinction risk. NAMMCO Scientific Publications 6:33-56. https://doi.org/10.7557/3.2720

HELCOM (2018) Nutritional status of seals. HELCOM core indicator report. HELCOM

Hickie BE, Muir DCG, Addison RF, Hoekstra PF (2005) Development and application of bioaccumulation models to assess persistent organic pollutant temporal trends in arctic ringed seal (Phoca hispida) populations. Science of the Total Environment 351–352:413–426. https://doi.org/10.1016/j.scitotenv.2004.12.085

Härkönen T (2016) Halichoerus grypus (Baltic Sea subpopulation). The IUCN Red List of Threatened Species 2016 (e.T74491261A74491289)

Iverson S (2002) Blubber. Pages 107–112 *in* B. G. W. W. F. Perrin, J. G. M. Thewissen, editor. Encyclopedia of Marine Mammals. Academic Press, San Diego, California, USA

Iverson SJ (1993) Milk secretion in marine mammals in relation to foraging: can milk fatty acids predict diet? Symp Zool Soc Lond 66:263-291

Jager T, Albert C, Preuss TG, Ashauer R (2011) General Unified Threshold Model of Survival - a Toxicokinetic-Toxicodynamic Framework for Ecotoxicology. Environmental Science & Technology 45:2529–2540

Jefferson TA, Webber MA, Pitman RL (2008) Marine mammals of the world: A comprehensive guide to their identification. Academic Press, London

Jüssi M, Härkönen T, Jüssi I, Helle E (2008) Decreasing ice coverage will reduce the breeding success of Baltic grey seal (Halichoerus grypus) females. Ambio 37:80–85. https://doi.org/10.1579/0044-7447

Kauhala K, Ahola MP, Kunnasranta M (2014) Decline in the pregnancy rate of Baltic grey seal females during the 2000s. Annales Zoologici Fennici 51:313–324. https://doi.org/10.5735/086.051.0303

Kauhala K, Backlin BM, Raitaniemi J, Harding KC (2017) The effect of prey quality and ice conditions on the nutritional status of Baltic gray seals of different age groups. Mammal Research 62:351-362. https://doi.org/10.1007/s13364-017-0329-x

Klanjscek T, Nisbet RM, Caswell H, Neubert MG (2007) A model for energetics and bioaccumulation in marine mammals with applications to the right whale. Ecological Applications 17:2233-2250. https://doi.org/10.1890/06-0426.1

Kleiber M (1962) The Fire of Life. John Wiley & Sons, New York

Kooijman B, Lika D, Augustine S (2019) Add-my-Pet.

Kooijman SALM (2000) Dynamic Energy and Mass Budgets in Biological Systems. Cambridge University Press, Cambridge, UK

Lacy RC, Miller PS, Traylor-Holzer K (2018) Vortex 10 - A Stochastic Simulation of the Extinction Process - User's Manual. IUCN SSC Conservation Breeding Group & Chicago Zoological Society, Chicago

Lang SLC, Iverson SJ, Bowen WD (2011) The Influence of Reproductive Experience on Milk Energy Output and Lactation Performance in the Grey Seal (Halichoerus grypus). PLoS ONE 6:e19487. https://doi.org/10.1371/journal.pone.0019487

Lundström K, Hjerne O, Lunneryd S-G, Karlsson O (2010) Understanding the diet composition of marine mammals: grey seals (Halichoerus grypus) in the Baltic Sea. ICES Journal of Marine Science 67:1230–1239. https://doi.org/10.1093/icesjms/fsq022

Peters RH (1983) The ecological implications of body size. Cambridge University Press

Roos AM, Bäcklin B-MVM, Helander BO, Rigét FF, Eriksson UC (2012) Improved reproductive success in otters (Lutra lutra), grey seals (Halichoerus grypus) and sea eagles (Haliaeetus albicilla) from Sweden in relation to concentrations of organochlorine contaminants. Environmental Pollution 170:268-275. https://doi.org/10.1016/j.envpol.2012.07.017

Segerstedt R (2019) PCB's Bioaccumulation Levels in the Food Chain of Marine Top Predators in the Baltic Sea. University of Gothenburg, Gothenburg, Sweden, Degree project for Master of Science in Conservation Biology

Subramanian AN, Tanabe S, Tatsukawa R (1988) Use of organochlorines as chemical tracers in determining some reproductive parameters in Dalli-type Dall's porpoise Phocoenoides dalli. Marine Environmental Research 25:61–74

Svensson CJ, Eriksson A, Harkonen T, Harding KC (2010) Detecting Density Dependence in Recovering Seal Populations. Ambio

Tverin M, Esparza-Salas R, Stromberg A, Tang P, Kokkonen I, Herrero A, Kauhala K, Karlsson O, Tiilikainen R, Sinisalo T, Kakela R, Lundstrom K (2019) Complementary methods assessing short and long-term prey of a marine top predator – Application to the grey seal-fishery conflict in the Baltic Sea. PLoS ONE 14:1-26. https://doi.org/10.1371/journal.pone.0208694
